# Total Syntheses of Marrubiin and Related Labdane Diterpene Lactones

**DOI:** 10.3390/molecules25071610

**Published:** 2020-04-01

**Authors:** Yukari Sakagami, Naoki Kondo, Yuki Sawayama, Hiroyuki Yamakoshi, Seiichi Nakamura

**Affiliations:** Graduate School of Pharmaceutical Sciences, Nagoya City University, 3-1 Tanabe-dori, Mizuho-ku, Nagoya 467-8603, Japan; yukarinnuu@gmail.com (Y.S.); c202709@ed.nagoya-cu.ac.jp (N.K.); ys020205@gmail.com (Y.S.); yamakoshi@phar.nagoya-cu.ac.jp (H.Y.)

**Keywords:** marrubiin, total synthesis, labdane, terpenoid, chiral building block, Pauson-Khand reaction

## Abstract

Total syntheses of the labdane diterpene lactones marrubiin, marrulibacetal, desertine, marrulibacetal A, marrubasch F, cyllenine C, marrulanic acid, and marrulactone are described. The *trans*-decalin moiety of these molecules was constructed in a stereoselective manner by a Pauson-Khand reaction, and the resultant cyclopentenone was oxidatively cleaved for formation of the lactone ring. Elongation of the side chain at C9 was achieved by an epoxide-opening reaction with a variety of nucleophiles, and the functional group manipulations completed the syntheses of these natural products. Stereochemistries of desertine could be established by the transformations.

## 1. Introduction

**Figure 1:**
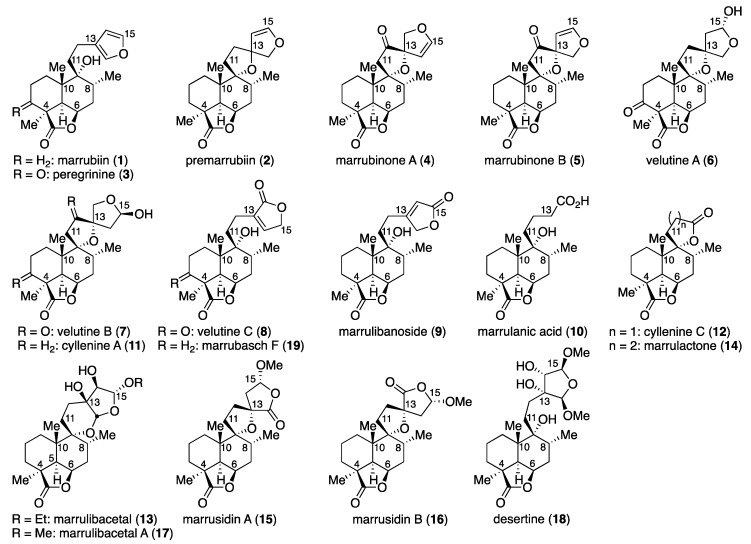
Structures of marrubiin and related natural products.

Flowering plants of the genus *Marrubium* (Lamiaceae) are distributed in the Mediterranean and temperate regions of the Eurasian zone, and most of the plants are used in folk medicine [1]. The therapeutic properties of these herbs, including anti-inflammatory, hypoglycemic, analgesic, antispasmodic, vasorelaxant, and anti-diabetic effects, are attributed in part to marrubiin (**1**), which was first isolated in 1842 from *Marrubium vulgare* and is a prominent member of the labdane diterpene lactones [2] (Figure 1). This furanoid natural product, formed from premarrubiin (**2**), was also reported to inhibit KCl-induced contraction of the rat aorta in a concentration-dependent manner [3]. The important pharmacological action of this family of mints prompted phytochemical analysis, leading to the isolation and characterization of a number of labdane diterpene lactones [4]: peregrinine (**3**) [5], marrubinones A (**4**) and B (polyodonine, **5**) [6,7], velutines A (**6**), B (**7**) and C (**8**) [8], marrulibanoside (**9**) [9], marrulanic acid (**10**) [10], cyllenines A (**11**) and C (**12**) [11], marrulibacetal (**13**), marrulactone (**14**) [12], marrusidins A (**15**) and B (**16**) [13], marrulibacetal A (**17**), desertine (**18**) [14], and marrubasch F (**19**) [15] have been reported to date [16]. These natural products are biosynthesized from (*E*,*E*,*E*)-geranylgeranyl diphosphate (GGPP) through peregrinol diphosphate synthase (CPS1)-catalyzed bicyclization, followed by 9,13-epoxylabd-14-ene synthase (ELS)-catalyzed formation of tetrahydropyran and regiospecific oxygenations with P450s [17,18]. Despite their remarkable biological activities, only a total synthesis of marrubiin (**1**) in a racemic form [19] and semi-syntheses of premarrubiin (**2**), marrulibanoside (**9**), marrubasch F (**19**) and (13*R*)-9α,13α-epoxylabda-6β(19),16 (15)-diol dilactone from marrubiin (**1**) have been reported by Mangoni and co-workers [20].

**Figure 2:**
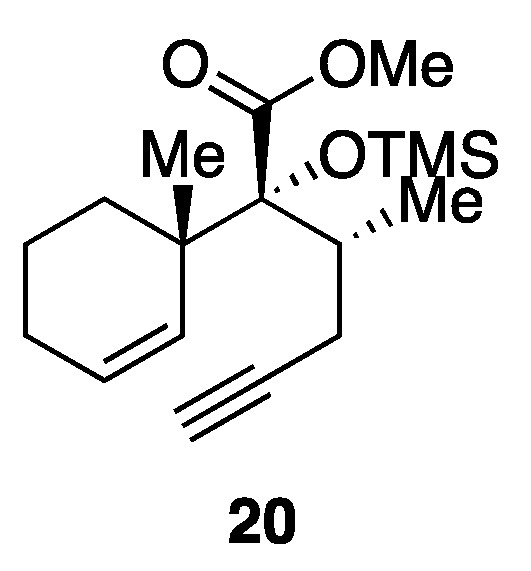
Structure of building block **20**.

Recently, we have developed a method for stereocontrolled preparation of enyne **20** (Figure 2, TMS = trimethylsilyl) by exploiting a ring-contractive coupling between an α-bromo-δ-valerolactone and a secondary alcohol, and subsequent Ireland−Claisen rearrangement [21,22]. Since compound **20** embodies the C8−C10 stereotriad of C9-oxygenated labdane diterpenoids, compound **20** can serve as a useful chiral building block for the synthesis of pharmacologically interesting, marrubiin-related natural products. In this article, we describe the total syntheses of members of the marrubiin family including marrubiin (**1**), marrulanic acid (**10**), cyllenine C (**12**), marrulibacetal (**13**), marrulactone (**14**), marrulibacetal A (**17**), desertine (**18**), and marrubasch F (**19**) [23].

## 2. Results and Discussion

### 2.1. Retrosynthetic Analysis

**Scheme 1:**
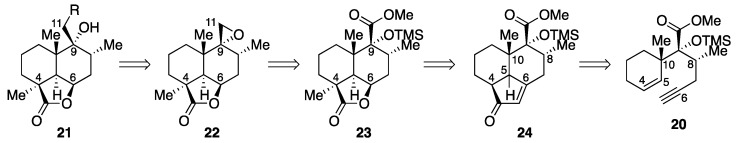
Retrosynthetic analysis.

Our retrosynthetic analysis of marrubiin-related natural products is depicted in Scheme 1. Since the difference between the target molecules lies in the variation in the side chain at C9, we planned to install the full C9 side chain late in the synthesis by a nucleophilic ring-opening reaction of advanced epoxide intermediate **22**, which could be derived from ester **23** through a chemoselective reduction of the ester functionality in the presence of the lactone moiety. While the methyl group at C4 would be introduced from the less-hindered diastereoface by an enolate alkylation, C5−C6 bond formation, carboxylation at C4, and oxidative cleavage of the C−C bond at C6 were required for the conversion of enyne **20** to lactone **23**. In light of these requirements, we chose to use the intramolecular Pauson−Khand reaction (PKR) [24,25] to fashion the six-membered ring and introduce the carbonyl group at C4. Although some concern arose over the formation of *cis*-fused isomer *cis*-**24** from enyne **20** considering that [RhCl(CO)_2_]_2_-catalyzed PKRs of substituted 5-(pent-4-ynyl)cyclohexa-1,3-dienes are precedented to afford *cis*-decalin derivatives stereoselectively [26], we expected that the stereocenter at C5 would be epimerized after oxidative cleavage of the double bond at C6.

### 2.2. Synthesis of Advanced Intermediate **22**

**Scheme 2:**
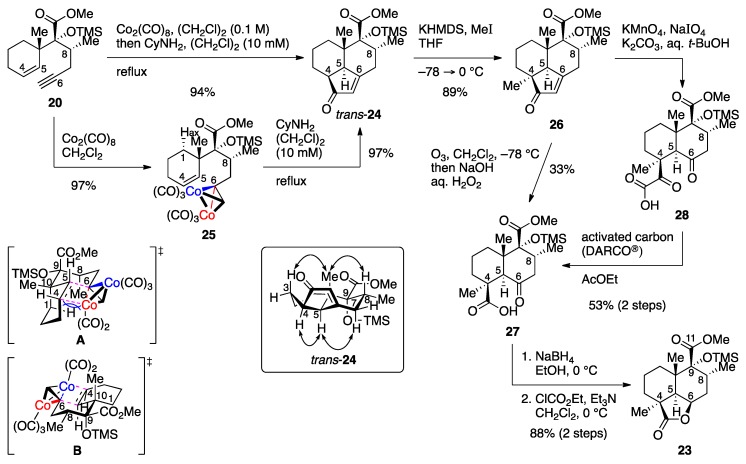
Construction of the tricyclic lactone. KHMDS = potassium bis(trimethylsilyl)amide, THF = tetrahydrofuran.

The synthesis of advanced intermediate **22** commenced with the key PKR of enyne **20** (Scheme 2). Initial attempts to cyclize dicobalt complex **25**, prepared by treatment of enyne **20** with Co_2_(CO)_8_ in CH_2_Cl_2_ (97% yield), upon heating in refluxing acetonitrile failed to produce any of the PKR products, leading to decomplexation. After considerable experimentation, we found that the desired tricyclic compound **24** could be produced with the aid of a promoter, with CyNH_2_ (Cy = cyclohexyl) [27] being optimal for this purpose. Gratifyingly, the product, obtained in (CH_2_Cl)_2_ at a substrate concentration of 10 mM under optimized conditions in 97% yield, proved to be the desired stereoisomer *trans*-**24** as confirmed by the absence of a cross-peak between C10-C*H*_3_ and C5-*H* in the nuclear Overhauser effect spectroscopy (NOESY) spectrum. It has been suggested on the basis of quantum mechanical studies that the stereochemistry of PKR is determined by the irreversible olefin insertion step [28]. Since the chair−chairlike transition state (TS) **A** suffers from steric repulsion between C8-C*H*_3_ and C1-*H*_ax_, the reaction proceeded through chair−boatlike TS **B**, leading to the exclusive formation of *trans*-decalin *trans*-**24**.

To enhance the synthetic utility of PKR, a number of catalytic methods have been reported, and a variety of metal complexes have been employed for this purpose [29]. However, our attempts to carry out the catalytic reaction with enyne **20** met with failure: the use of Krafft conditions [30] resulted in recovery of enyne **20** in 49% yield, whereas a complicated mixture was obtained when using 2-naphthaldehyde as a CO donor in the [RhCl(cod)]_2_-catalyzed reaction [31]. In contrast to these unsuccessful results, the transformation of enyne **20** to enone *trans*-**24** could be refined to a one-pot procedure, wherein the cobalt complex was formed in (CH_2_Cl)_2_ and heated under reflux after addition of CyNH_2_ and 10-fold dilution with (CH_2_Cl)_2_. As anticipated, alkylation of the potassium enolate generated from *trans*-**24** with MeI in THF took place exclusively from the less-hindered α-face to provide enone **26** in 89% yield.

With tricyclic compound **26** in hand, efforts were next focused on the transformation of the cyclopentenone moiety to the corresponding γ-butyrolactone. With regard to the oxidative cleavage of the cyclopentenone ring, it was found that desired γ-ketocarboxylic acid **27** could be obtained upon exposure of enone **26** to ozone in CH_2_Cl_2_ at −78 °C followed by either reductive (Me_2_S) or oxidative (H_2_O_2_, NaOH) workup [32], but the reaction suffered from low yield (33%) and reproducibility issues. While α-ketocarboxylic acid **28**, detected as a byproduct, could be converged to γ-ketocarboxylic acid **27** upon treatment with H_2_O_2_ in aqueous NaOH/THF, no improvement in overall yield was observed. We then investigated a stepwise approach via α-ketocarboxylic acid **28**. After an extensive screening of oxidants, the KMnO_4_/NaIO_4_ system proved to be effective for the conversion of enone **26** to **28**. To our surprise, submission of crude α-ketocarboxylic acid **28** to activated carbon for removal of the residual Mn salt to avoid decomposition of H_2_O_2_ effected desired decarbonylation. As a consequence, γ-ketocarboxylic acid **27** could be obtained in 53% yield over two steps. The reason for the decarbonylation is unclear at present, but the possibility of involvement of contaminated Mn salt was excluded due to the fact that the reaction occurred from α-ketocarboxylic acid **28** produced by ozonolysis [33]. To the best of our knowledge, this is the first example of activated carbon-mediated decarbonylation of α-ketocarboxylic acid. Lactone formation from γ-ketocarboxylic acid **27** was achieved following the precedents of Wheeler [34] and Mangoni [19]: selective reduction of the carbonyl group at C6 with NaBH_4_ in EtOH at 0 °C was followed by lactonization through a mixed anhydride upon treatment with ClCO_2_Et in the presence of Et_3_N in CH_2_Cl_2_ at 0 °C to give lactone **23** in 88% yield over two steps [35].

**Scheme 3:**
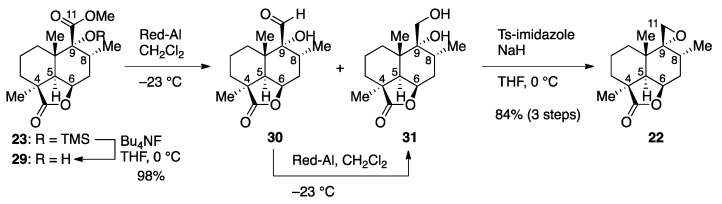
Completion of the synthesis of advanced intermediate **22**. Ts = *p*-toluenesulfonyl.

The remaining operation necessary for the synthesis of advanced intermediate **22** involved chemoselective reduction of the ester functionality in the presence of the lactone moiety (Scheme 3). As a prelude to the conversion, TMS ether **23** was converted to α-hydroxyester **29** by exposure to Bu_4_NF in THF at 0 °C (98% yield). With regard to the reduction, the use of NaBH_4_ or LiBH_4_ resulted in no reaction even at the reflux temperature, whereas the lactone moiety in **29** was selectively reduced with diisobutylaluminum hydride (DIBALH) in CH_2_Cl_2_ at −78 °C. We were gratified to find that the desired chemoselective reduction could be achieved by the use of sodium bis(2-methoxyethoxy)aluminum hydride (Red-Al^®^) as a reducing agent. It should be noted that the hydroxyl-directed reduction was accompanied by some lactone reduction when performed using Et_2_O or THF as a solvent or when performed at temperatures above −20 °C. The use of CH_2_Cl_2_ proved optimal in terms of chemoselectivity and solubility of substrate **29**, but the collapse of the five-membered aluminate intermediate was retarded under the optimal conditions, resulting in the formation of a mixture of aldehyde **30** and 1,2-diol **31** after aqueous workup. Thus, the mixture needed to be subjected again to Red-Al^®^ in CH_2_Cl_2_ at −23 °C for the full reduction to 1,2-diol **31**. The synthesis of advanced intermediate **22** was completed upon treatment of 1,2-diol **31** with *p*-toluenesulfonylimidazole and NaH in THF at 0 °C (84% yield over three steps).

### 2.3. Total Syntheses of Marrubiin and Related Labdane Diterpene Lactones

**Scheme 4:**
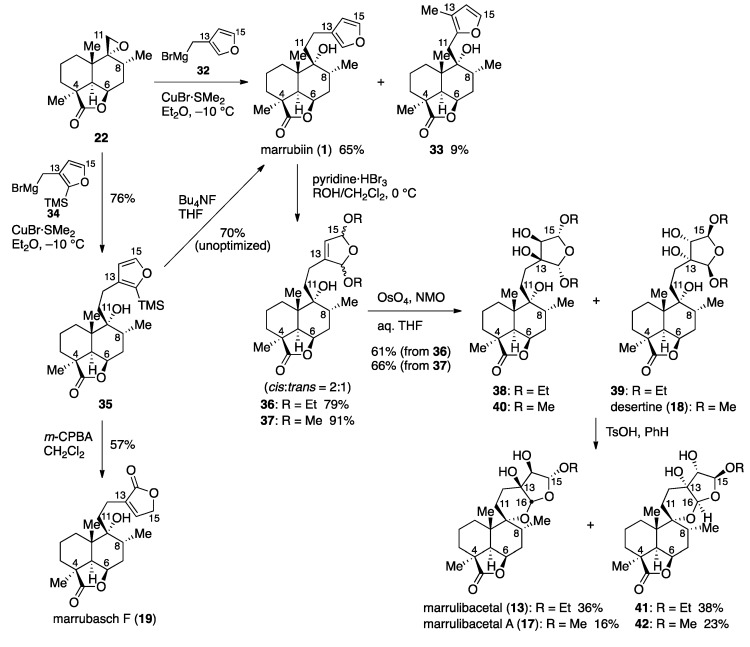
Total syntheses of marrubiin (**1**), marrulibacetal (**13**), marrulibacetal A (**17**), and marrubasch F (**19**) from epoxide **22**.

Having established a route to epoxide **22**, the stage was now set for elongation of the side chain for total syntheses. In this regard, Welch and co-workers reported the synthesis of isomarrubiin (C9-*epi*-marrubiin) by a CuI-catalyzed epoxide ring-opening reaction of the C9-epimer of epoxide **22** in Et_2_O at room temperature (40% yield) [36]. With this reaction serving as a reference, we first examined Cu(I)-catalyzed epoxide-opening reaction with Grignard reagent **32** [37] (Scheme 4). After some experimentation, we found that the use of a stoichiometric amount of CuBr·SMe_2_ was more effective, providing marrubiin (**1**), [α${]}_{D}^{21}$ +34.4 (*c* 1.04, CHCl_3_) [lit. [38], [α${]}_{D}^{20}$ +35.8 (*c* 3.1, CHCl_3_)] in 65% yield. However, Grignard reagent **32** is prone to isomerization due to the high acidity of the furan 2-position, resulting in the formation of isomer **33** in 9% yield [39]. This problem was circumvented by the use of Grignard reagent **34** [40], the TMS group of which was uneventfully removed with Bu_4_NF in THF [41] after the epoxide-opening reaction.

An inspection of the structures of marrulibacetal (**13**) and marrulibacetal A (**17**) revealed that the highly oxidized tetrahydrofuran ring of these molecules could be formed by oxidation of the furan moiety in marrubiin (**1**) followed by internal acetalization. While unprecedented in the transformation of the marrubiin class labdane diterpenoids, successive oxidations of a furan ring have been documented in semi-synthesis of the neoclerodane diterpene natural products salvinicins A and B by Prisinzano and co-workers [42]. Furthermore, Frontana-Uribe and co-workers reported construction of the [6,6,5,5]-tetracyclic framework, in which 4,5,6,7a-tetrahydro-2*H*-furo[2,3-*b*]pyran is spirolinked to a *trans*-decalin ring system, by an electrochemical oxidation of hispanolone [43]. When marrubiin (**1**) was exposed to pyridine tribromide in EtOH/CH_2_Cl_2_ at 0 °C, oxidative acetalization occurred to give bisacetal **36** in 79% yield with a *cis*/*trans* ratio of 2:1, albeit with no sign of internal acetalization in contrast to Frontana-Uribe’s work. This result is attributed to the conformational constraint imposed by the lactone ring. The chemical yield was improved to 84% in the presence of K_2_CO_3_ as an acid scavenger. As expected from the precedent [44], the reaction rate of bisacetal **36** with OsO_4_/4-methylmorpholine *N*-oxide (NMO) in aqueous THF was influenced by the substrate structure: the reaction of a 1:1 mixture of diastereomers with the *cis* relative configuration at room temperature proceeded to completion within 1 h to give diols **38** and **39** in a stereoselective manner, whereas the *trans*-isomers were almost recovered unchanged under these conditions. Although the remaining *trans*-isomers could be consumed at an elevated temperature after prolonged reaction times, the lower π-facial selectivity (desired:undesired = 1:1.2), together with the fact that recovered *trans*-isomers could be isomerized to *cis*-isomers upon exposure to pyridinium *p*-toluenesulfonate (PPTS) in EtOH for 1 h, prompted us to perform the reaction at room temperature. Submission of an inseparable mixture of diols **38** and **39** to TsOH in benzene effected internal transacetalization, affording marrulibacetal (**13**), [α${]}_{D}^{27}$−21.7 (*c* 1.16, CHCl_3_) [lit. [12], [α${]}_{D}^{25}$−13.1 (*c* 0.29, CHCl_3_)], in 36% yield along with isomer **41** (38% yield) and other diastereomers (1:1, 18% combined yield). It is noteworthy that the dehydration of commercially available benzene with 3 Å molecular sieves prior to use affected the transacetalization, leading to the decomposition of substrates, although the reason is unclear at present.

Following the same reaction sequence using MeOH instead of EtOH, marrulibacetal A (**17**), [α${]}_{D}^{21}$−14.0 (*c* 1.69, CHCl_3_) [lit. [14], [α${]}_{D}^{25}$−10.77 (the solvent and concentration were not reported)], could be synthesized from marrubiin (**1**). It should be mentioned that one of two diastereomers, obtained as an inseparable mixture in a ratio of 1:1 by dihydroxylation of bisacetal **37**, matched by ^1^H-NMR with desertine (**18**), although the chemical correlation to establish the stereochemistry will be presented later (vide infra). The low chemical yield (16%) of **17** was due to the formation of two isomers with the *trans* H15/H16 stereochemistry in 38% yield.

Kuwajima and Urabe reported that 2-(trimethylsilyl)furans could be oxidized regioselectively with peracetic acid [45], and the method was successfully applied for the synthesis of substituted Δ^2^-butenolides by Goldsmith, Liotta, and co-workers [46]. On the basis of these precedents, we next examined the oxidation of 2-(trimethylsilyl)furan **35** for the conversion to marrubasch F (**19**). Screening of peracids revealed that the use of *m*-chloroperoxybenzoic acid (*m*-CPBA) (57% yield) and peracetic acid (52% yield) afforded **19**, mp 195−196 °C, [α${]}_{D}^{21}$ + 41.1 (*c* 0.53, CHCl_3_) [lit. [20], mp 191−193 °C, [α${]}_{D}$ +41.5 (*c* 1.00, CHCl_3_)], with *m*-CPBA being the optimal oxidizing agent, whereas only desilylation occurred to give marrubiin (**1**) with either performic acid or magnesium monoperoxyphthalate (MMPP).

**Scheme 5:**
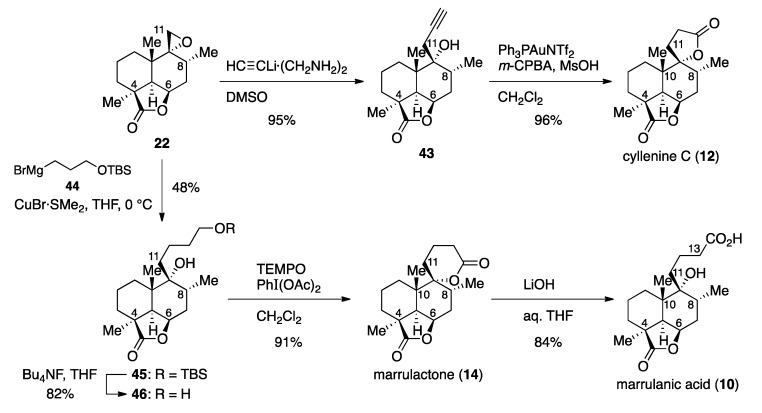
Total syntheses of cyllenine C (**12**), marrulactone (**14**), and marrulanic acid (**10**) from epoxide **22**. DMSO = dimethyl sulfoxide, Tf = trifluoromethanesulfonyl, Ms = methanesulfonyl, TBS = *tert*-butyldimethylsilyl.

Having synthesized marrubiin (**1**) and natural products possessing the same carbon framework in a higher oxidation state, we next addressed the conversion of epoxide **22** to natural products **12** and **14**, which required two- and three-carbon nucleophiles, respectively (Scheme 5). Fortunately, epoxide **22** underwent nucleophilic ring-opening reaction with commercially available lithium acetylide ethylenediamine complex, affording alcohol **43** in 95% yield. Oxidative lactonization of homopropargyl alcohol **43** could be attained by the gold(I)-catalyzed cycloisomerization/oxidation sequence under Ye conditions [47], completing the synthesis of cyllenine C (**12**). On the other hand, the use of Grignard reagent **44** [48] as a three-carbon nucleophile under conditions identical to those with **32** gave alcohol **45** (48% yield), which was desilylated with Bu_4_NF in THF to furnish 1,5-diol **46** in 82% yield. δ-Lactone could be formed upon oxidation of 1,5-diol **46** with 2,2,6,6-tetramethylpiperidin-1-oxyl (TEMPO)/PhI(OAc)_2_ in CH_2_Cl_2_ according to the Forsyth protocol [49] to give marrulactone (**14**) in 91% yield. Since the γ-lactone in **14** proved more reluctant to hydrolyze, regioselective saponification could be realized with LiOH in THF, providing marrulanic acid (**10**) in 84% yield. Although synthetic compounds **12**, **14**, and **10** would be identical to natural products as judged by ^1^H and ^13^C-NMR analysis (see Supplementary Materials), their specific rotations [[α${]}_{D}^{24}$ + 22.1 (*c* 0.69, CH_2_Cl_2_), [α${]}_{D}^{21}$ − 11.6 (*c* 0.61, CHCl_3_), and [α${]}_{D}^{27}$ + 25.4 (*c* 0.53, CHCl_3_), respectively] were inconsistent with those observed for natural cyllenine C (**12**), marrulactone (**14**), and marrulanic acid (**10**) [[α${]}_{D}^{20}$ + 11.82 (*c* 0.33, CH_2_Cl_2_) [11], [α${]}_{D}^{25}$ − 23.80 (*c* 0.22, CHCl_3_) [12], and [α${]}_{D}^{25}$ − 10.8 (*c* 1.2, CHCl_3_) [12], respectively]. This result is attributed to contamination of the impurity in these natural products, as detected by NMR spectroscopy.

**Scheme 6:**
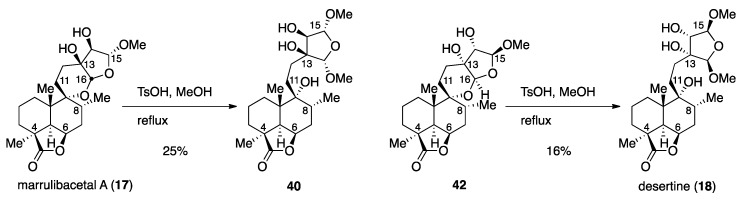
Conversion of marrulibacetal A (**17**) and its isomer **42** to triol **40** and desertine (**18**).

Of the eight natural products synthesized, desertine (**18**) could not be purified due to the difficulty of separation from its diastereomer **40**. With regard to this natural product, stereochemistries were determined by an NOESY experiment by Dijoux-Franca and co-workers, who indicated two structures differing in the configuration at C15 [14]. Furthermore, evidence that supports the stereochemical relationship between the decalin and tetrahydrofuran moieties, separated by the C11−C12 two-carbon bridge, was not provided in their report. Therefore, we felt compelled to perform experiments to determine the stereochemistries of **18**, and we found that exposure of marrulibacetal A (**17**) and its diastereomer **42** to TsOH in refluxing MeOH to effect transacetalization resulted in the formation of triol **40** and desertine (**18**), respectively, albeit in low yields (Scheme 6). These results suggest that the stereocenters at C13 and C14 of **18** have configurations opposite to those of **17**. Together with our previous observation that desertine was produced from *cis*-**37**, the stereochemistries of this natural product can be established as shown for **18**.

## 3. Materials and Methods

### Chemistry

#### 3.1.1. General Information

Optical rotations were recorded on a digital polarimeter with a sodium lamp (589 nm). Infrared (IR) spectra were recorded on an FT-IR spectrophotometer and absorbance bands are reported in wavenumber (cm^−1^). Proton nuclear magnetic resonance (^1^H-NMR) spectra were recorded with tetramethylsilane (δ_H_ 0.00), CHCl_3_ (δ_H_ 7.26), or CH_2_Cl_2_ (δ_H_ 5.32) as an internal standard. Coupling constants (*J*) are reported in hertz (Hz). Abbreviations of multiplicity are as follows: s, singlet; d, doublet; t, triplet; q, quartet; m, multiplet; br, broad. Data are presented as follows: chemical shift, multiplicity, coupling constants, integration, and assignment. Marrulibacatal numbering is used for proton assignments of all intermediates. Carbon nuclear magnetic resonance (^13^C-NMR) spectra were recorded with CDCl_3_ (δ_C_ 77.0), CD_2_Cl_2_ (δ_C_ 53.84), or acetone-*d*_6_ (δ_C_ 29.84) as an internal standard. High-resolution mass spectra (HRMS) were recorded either by electrospray ionization (ESI) using a time-of-flight (TOF) analyzer, or by electron impact (EI) using a magnetic sector analyzer. Column chromatography was carried out on silica gel 60 N (63−210 μm or 40−50 μm). Analytical thin layer chromatography (TLC) was carried out with 0.25 mm silica gel plates. Visualization was accomplished with ultraviolet light and anisaldehyde stain, followed by heating. Reagents and solvents were purified by standard means or used as received unless otherwise noted. Dehydrated dichloromethane (CH_2_Cl_2_) and tetrahydrofuran (THF, stabilizer free) were purchased. Cyclohexylamine was distilled from calcium hydride. 4 Å molecular sieves was finely ground in mortar and heated in vacuo at 180 °C for 4 h. *p*-Toluenesulfonylimidazole [50], (3-furylmethyl)magnesium bromide [37], CuBr·SMe_2_ [51], [2-(trimethylsilyl)furan-3-yl]methanol [52], and 3-(*tert*-butyldimethylsilyl)oxypropylmagnesium bromide (**44**) [48] were prepared according to literature procedures. All reactions were conducted under an argon atmosphere unless otherwise noted.

#### 3.1.2. Experimental Procedures and Compound Data

*Methyl [2R,2(1S),3R]-3-methyl-2-(1-methylcyclohex-2-en-1-yl)-2-(trimethylsilyl)oxyhex-5-ynoate dicobalt hexacarbonyl complex* (**25**). Co_2_(CO)_8_ (663 mg, 1.94 mmol) was added to an ice-cooled (0 °C) solution of enyne **20** (522 mg, 1.62 mmol) in CH_2_Cl_2_ (16 mL). After 1.5 h of stirring at room temperature, the reaction mixture was concentrated in vacuo, and the reddish brown residue was chromatographed twice (silica gel 38 g, 20:1 *n*-hexane/AcOEt) to give dicobalt complex **25** (958 mg, 97%) as a dark red oil. *R_f_* 0.51 (20:1 *n*-hexane/AcOEt); IR (neat) 2951, 2091, 2048, 2016, 1748, 1250, 1175, 1138, 841 cm^−1^; ^1^H-NMR (500 MHz, CDCl_3_) δ 0.19 (s, 9H, Si(C*H*_3_)_3_), 0.87 (d, *J* = 6.6 Hz, 3H, C8-C*H*_3_), 1.00 (s, 3H, C10-C*H*_3_), 1.42 (br d, *J* = 13.8 Hz, 1H, one of C1-*H*_2_), 1.63 (m, 1H, one of C2-*H*_2_), 1.71 (m, 1H, one of C2-*H*_2_), 1.89−2.03 (m, 3H, one of C1-*H*_2_, C3-*H*_2_), 2.28 (ddq, *J* = 2.7, 11.4, 6.6 Hz, 1H, C8-*H*), 2.60 (ddd, *J* = 1.0, 11.4, 14.7 Hz, 1H, one of C7-*H*_2_), 3.49 (br d, *J* = 14.7 Hz, 1H, one of C7-*H*_2_), 3.71 (s, 3H, CO_2_C*H*_3_), 5.71 (ddd, *J* = 1.7, 5.2, 10.2 Hz, 1H, C4-*H*), 5.82 (br d, *J* = 10.2 Hz, 1H, C5-*H*), 6.01 (br t, *J* = 1.0 Hz, 1H, C6-C*H*); ^13^C-NMR (125.7 MHz, CDCl_3_) δ 2.8 (CH_3_), 16.1 (CH_3_), 20.0 (CH_2_), 24.4 (CH_2_), 25.1 (CH_3_), 31.5 (CH_2_), 36.9 (CH_2_), 41.3 (CH), 42.4 (C), 51.5 (CH_3_), 74.2 (C), 88.5 (C), 95.8 (CH), 126.8 (CH), 132.8 (CH), 175.1 (C), 200.0 (C).

*Methyl (1S,6R,7R,8S,12R)-6,8-dimethyl-2-oxo-7-(trimethylsilyl)oxytricyclo[6.3.1.0^4,12^]dodec-3-ene-7-carboxylate* (*trans*-**24**). A solution of dicobalt complex **25** (150 mg, 0.247 mmol) in 1,2-dichloroethane (5 mL plus 2 × 2.5 mL rinse) was added to a refluxing solution of cyclohexylamine (0.17 mL, 1.49 mmol) in 1,2-dichloroethane (15 mL), and the resulting mixture was refluxed for 1.5 h. After cooling, the mixture was filtered through a Celite pad, and the filtrate was concentrated in vacuo. Purification of the residue (503 mg) by column chromatography (silica gel 30 g, 3:1 *n*-hexane/AcOEt) gave enone *trans*-**24** (84.0 mg, 97%) as a white solid. *R_f_* 0.42 (3:1 *n*-hexane/AcOEt); mp 75−76 °C (colorless plates from *n*-hexane); [α${]}_{D}^{25}$ + 62.1 (*c* 1.34, CHCl_3_); IR (KBr) 2945, 1736, 1694, 1620, 1458, 1260, 1184, 1096, 1034, 982, 843 cm^−1^; ^1^H-NMR (500 MHz, CD_2_Cl_2_) δ 0.20 (s, 9H, Si(C*H*_3_)_3_), 0.62 (s, 3H, C10-C*H*_3_), 0.91 (d, *J* = 6.2 Hz, 3H, C8-C*H*_3_), 1.01 (dt, *J* = 13.4, 7.4 Hz, 1H, one of C1-*H*_2_), 1.51−1.63 (m, 2H, one of C2-*H*_2_, one of C3-*H*_2_), 1.69 (m, 1H, one of C2-*H*_2_), 1.80 (m, 1H, one of C3-*H*_2_), 1.89 (ddd, *J* = 4.5, 8.9, 13.4 Hz, 1H, one of C1-*H*_2_), 2.16 (t, *J* = 13.9 Hz, 1H, one of C7-*H*_2_), 2.47 (ddd, *J* = 7.3, 8.5, 9.5 Hz, 1H, C4-*H*), 2.56−2.61 (m, 2H, one of C7-*H*_2_, C8-*H*), 3.16 (d, *J* = 7.3 Hz, 1H, C5-*H*), 3.70 (s, 3H, CO_2_C*H*_3_), 5.80 (s, 1H, =C*H*CO); ^13^C-NMR (125.7 MHz, CD_2_Cl_2_) δ 2.6 (CH_3_), 18.3 (CH_3_), 19.7 (CH_2_), 19.9 (CH_2_), 21.1 (CH_3_), 28.8 (CH_2_), 34.9 (CH_2_), 37.0 (CH), 43.7 (CH), 45.0 (CH), 45.8 (C), 51.8 (CH_3_), 86.1 (C), 126.0 (CH), 172.9 (CH), 179.4 (C), 211.9 (C); HRMS (ESI) *m*/*z* [M + Na]^+^ calcd for C_19_H_30_O_4_SiNa 373.1806; found 373.1823.

*One-pot reaction*. Co_2_(CO)_8_ (4.08 g, 11.9 mmol) was added to an ice-cooled (0 °C) solution of enyne **20** (3.20 g, 9.93 mmol) in 1,2-dichloroethane (100 mL), and the mixture was stirred at room temperature for 3 h. The resulting dark red suspension was diluted with 1,2-dichloroethane (900 mL), and cyclohexylamine (8.0 mL, 70.7 mmol) was added. After 5 h of heating at reflux, the reaction mixture was cooled to room temperature, and the volatile elements were removed in vacuo. The residue was suspended in 4:1 *n*-hexane/AcOEt and filtered through a Celite pad. Evaporation of the filtrate in vacuo furnished the crude product (5.75 g), which was purified by column chromatography (silica gel 200 g, 25:1 → 10:1 → 4:1 → 1:1 *n*-hexane/AcOEt) to give enone *trans*-**24** (3.26 g, 94%) as a white solid.

*Methyl (1S,6R,7R,8S,12R)-1,6,8-trimethyl-2-oxo-7-(trimethylsilyl)oxytricyclo[6.3.1.0^4,12^]dodec-3-ene-7-carboxylate* (**26**). KHMDS in toluene (0.5 M, 6.2 mL, 3.10 mmol) was added to a cooled (−78 °C) solution of enone *trans*-**24** (988 mg, 2.82 mmol) in THF (30 mL). After 40 min of stirring, iodomethane (0.23 mL, 3.69 mmol) was added, and the resulting mixture was stirred at 0 °C for 40 min. The reaction was quenched with saturated aqueous NH_4_Cl (30 mL), and the mixture was extracted with AcOEt (3 × 60 mL). The combined organic extracts were washed with brine (2 × 30 mL), and dried over anhydrous Na_2_SO_4_. Filtration and evaporation in vacuo furnished the crude product (1.16 g), which was purified by column chromatography (silica gel 30 g, 6:1 *n*-hexane/AcOEt) to give methylated product **26** (915 mg, 89%) as a yellow oil. *R_f_* 0.40 (4:1 *n*-hexane/AcOEt); [α${]}_{D}^{22}$ +72.2 (*c* 0.95, CHCl_3_); IR (neat) 3433, 2953, 1738, 1703, 1624, 1458, 1252, 1186, 1103, 1042, 841 cm^−^^1^; ^1^H-NMR (500 MHz, CDCl_3_) δ 0.20 (s, 9H, Si(C*H*_3_)_3_), 0.71 (s, 3H, C10-C*H*_3_), 0.91 (d, *J* = 6.3 Hz, 3H, C8-C*H*_3_), 1.02 (ddd, *J* = 7.4, 9.2, 13.5 Hz, 1H, one of C1-*H*_2_), 1.11 (s, 3H, C4-C*H*_3_), 1.43 (m, 1H, one of (C*H*_2_)_2_), 1.60 (m, 1H, one of (C*H*_2_)_2_), 1.64−1.75 (m, 2H, two of (C*H*_2_)_2_), 1.92 (ddd, *J* = 2.8, 10.2, 13.5 Hz, 1H, one of C1-*H*_2_), 2.12 (m, 1H, one of C7-*H*_2_), 2.54−2.62 (m, 2H, one of C7-*H*_2_, C8-*H*), 2.67 (br s, 1H, C5-*H*), 3.73 (s, 3H, CO_2_C*H*_3_), 5.78 (t, *J* = 1.4 Hz, 1H, =C*H*CO); ^13^C-NMR (125.7 MHz, CDCl_3_) δ 2.5 (CH_3_), 17.7 (CH_2_), 18.0 (CH_3_), 21.1 (CH_3_), 26.6 (CH_2_), 27.3 (CH_3_), 28.0 (CH_2_), 34.8 (CH_2_), 36.4 (CH), 45.80 (C), 45.84 (C), 51.5 (CH_3_), 52.3 (CH), 85.7 (C), 123.8 (CH), 172.6 (C), 178.5 (C), 215.8 (C); HRMS (ESI) *m*/*z* [M + Na]^+^ calcd for C_20_H_32_O_4_SiNa 387.1962; found 387.1950.

*(1S,4aS,5R,6R,8aR)-5-(Methoxycarbonyl)-1,4a,6-trimethyl-8-oxo-5-(trimethylsilyl)oxydecahydronaphthalene-1-carboxylic acid* (**27**). KMnO_4_ (4.6 mg, 29.1 μmol) was added to a solution of NaIO_4_ (270 mg, 1.26 mmol) in H_2_O (7.0 mL), and the mixture was stirred for 30 min. To the mixture was added K_2_CO_3_ (23.0 mg, 0.166 mmol), followed by a solution of enone **26** (50.3 mg, 0.138 mmol) in *t*-BuOH (1.4 mL plus 2 × 1 mL rinse). After 24 h of stirring, the reaction was quenched with NaHSO_3_ (507 mg, 4.87 mmol), and the resulting mixture was extracted with AcOEt (3 × 20 mL). The combined organic extracts were washed with brine (2 × 15 mL), and dried over anhydrous Na_2_SO_4_. Filtration and evaporation in vacuo furnished the crude product (70.9 mg), which was used without further purification.

Activated carbon (708 mg) was added to an ice-cooled (0 °C) solution of the crude α-ketocarboxylic acid **28** (70.9 mg) in AcOEt (14 mL). After 63 h of stirring at room temperature, the resulting suspension was filtered through a Celite pad, and the filtrate was evaporated in vacuo. Purification of the crude product (74.7 mg) by column chromatography (silica gel 5.2 g, 7:1 *n*-hexane/AcOEt) gave γ-ketocarboxylic acid **27** (28.3 mg, 53%) as an amorphous solid. *R_f_* 0.60 (1:1 *n*-hexane/AcOEt); [α${]}_{D}^{21}$ + 62.5 (*c* 1.01, CHCl_3_); IR (neat) 2953, 1732, 1717, 1674, 1456, 1252, 1182, 1153, 1090, 843 cm^−1^; ^1^H-NMR (500 MHz, CDCl_3_) δ 0.24 (s, 9H, Si(C*H*_3_)_3_), 0.82 (dt, *J* = 3.5, 13.4 Hz, 1H, one of C3-*H*_2_), 0.88 (d, *J* = 6.4 Hz, 3H, C8-C*H*_3_), 0.97 (s, 3H, C10-C*H*_3_), 1.04 (d, *J* = 13.2 Hz, 1H, one of C1-*H*_2_), 1.29 (s, 3H, C4-C*H*_3_), 1.51 (m, 1H, one of C2-*H*_2_), 1.85 (m, 1H, one of C2-*H*_2_), 1.92 (dt, *J* = 4.3, 13.2 Hz, 1H, one of C1-*H*_2_), 2.26 (d, *J* = 13.4 Hz, 1H, one of C3-*H*_2_), 2.30 (dd, *J* = 4.3, 13.0 Hz, 1H, one of C7-*H*_2_), 2.51 (t, *J* = 13.0 Hz, 1H, one of C7-*H*_2_), 2.72 (ddq, *J* = 4.3, 13.0, 6.4 Hz, 1H, C8-*H*), 3.13 (s, 1H, C5-*H*), 3.75 (s, 3H, CO_2_C*H*_3_), 12.62 (br s, 1H, CO_2_*H*); ^13^C-NMR (125.7 MHz, CDCl_3_) δ 2.8 (CH_3_), 17.3 (CH_3_), 17.5 (CH_3_), 18.4 (CH_2_), 28.0 (CH_3_), 32.7 (CH_2_), 37.0 (CH), 38.7 (CH_2_), 43.6 (C), 46.4 (CH_2_), 49.6 (C), 52.0 (CH_3_), 60.3 (CH), 86.1 (C), 171.9 (C), 176.0 (C), 218.8 (C); HRMS (ESI) *m*/*z* [M + Na]^+^ calcd for C_19_H_32_O_6_SiNa 407.1860; found 407.1847.

*Methyl (1S,4R,6R,7R,8S,12R)-1,6,8-trimethyl-2-oxo-7-(trimethylsilyl)oxy-3-oxatricyclo[6.3.1.0^4,12^]dodecane-7-carboxylate* (**23**). A solution of γ-ketocarboxylic acid **27** (38.6 mg, 0.10 mmol) in EtOH (0.25 mL plus 2 × 0.25 mL rinse) was added to an ice-cooled (0 °C) solution of NaBH_4_ (4.3 mg, 0.11 mmol) in EtOH (0.5 mL). After 30 min of stirring at 0 °C, NaBH_4_ (3.7 mg, 0.098 mmol) was added, and the mixture was stirred at 0 °C for another 30 min. The reaction was quenched with saturated aqueous NH_4_Cl (5 mL), and the resulting mixture was partitioned between AcOEt (10 mL) and H_2_O (3 mL). The aqueous layer was extracted with AcOEt (5 × 10 mL), and the combined organic extracts were washed with brine (10 mL), and dried over anhydrous Na_2_SO_4_. Filtration and evaporation in vacuo furnished the crude product (42.7 mg), which was used without further purification.

Ethyl chloroformate (50 μL, 0.523 mmol) was added to an ice-cooled (0 °C) mixture of crude γ-hydroxycarboxylic acid (42.7 mg) and Et_3_N (0.10 mL, 0.717 mmol) in CH_2_Cl_2_ (1.7 mL), and the mixture was stirred for 30 min. The reaction was quenched with saturated aqueous NaHCO_3_ (5 mL), and the mixture was partitioned between *n*-hexane/AcOEt (10:1, 22 mL) and H_2_O (5 mL). The aqueous layer was extracted with *n*-hexane/AcOEt (10:1, 22 mL), and the combined organic extracts were washed with brine (20 mL) and dried over anhydrous Na_2_SO_4_. Filtration and evaporation in vacuo followed by column chromatography (silica gel 2.5 g, 9:1 *n*-hexane/AcOEt) afforded lactone **23** (32.6 mg, 88% for two steps) as a colorless oil. *R_f_* 0.44 (4:1 *n*-hexane/AcOEt); [α${]}_{D}^{22}$ + 24.6 (*c* 1.07, CHCl_3_); IR (neat) 2953, 1771, 1732, 1456, 1250, 1180, 1142, 1105, 1053, 841 cm^−1^; ^1^H-NMR (500 MHz, CDCl_3_) δ 0.15 (s, 9H, Si(C*H*_3_)_3_), 0.79 (d, *J* = 6.4 Hz, 3H, C8-C*H*_3_), 0.85 (dt, *J* = 13.0, 8.5 Hz, 1H, one of C1-*H*_2_), 1.10 (s, 3H, C10-C*H*_3_), 1.30 (s, 3H, C4-C*H*_3_), 1.42−1.56 (m, 3H, one of C2-*H*_2_, one of C3-*H*_2_, one of C7-*H*_2_), 1.69 (m, 1H, one of C2-*H*_2_), 1.77 (dd, *J* = 11.5, 13.0 Hz, 1H, one of C1-*H*_2_), 2.10−2.16 (m, 2H, one of C3-*H*_2_, C5-*H*), 2.22 (dd, *J* = 6.0, 16.2 Hz, 1H, one of C7-*H*_2_), 2.56 (m, 1H, C8-*H*), 3.73 (s, 3H, CO_2_C*H*_3_), 4.68 (br t, *J* = 5.3 Hz, 1H, C6-*H*); ^13^C-NMR (125.7 MHz, acetone-*d*_6_) δ 2.6 (CH_3_), 18.38 (CH_3_), 18.40 (CH_2_), 22.3 (CH_3_), 23.4 (CH_3_), 29.0 (CH_2_), 29.4 (CH_2_), 30.6 (CH), 31.9 (CH_2_), 40.4 (C), 44.1 (C), 45.6 (CH), 52.0 (CH_3_), 76.0 (CH), 86.1 (C), 173.6 (C), 183.2 (C); HRMS (ESI) *m*/*z* [M + Na]^+^ calcd for C_19_H_32_O_5_SiNa 391.1911; found 391.1917.

*Methyl (1S,4R,6R,7R,8S,12R)-7-hydroxy-1,6,8-trimethyl-2-oxo-3-oxatricyclo[6.3.1.0^4,12^]dodecane-7-carboxylate* (**29**). Bu_4_NF in THF (1.0 M, 2.0 mL, 2.0 mmol) was added to an ice-cooled (0 °C) solution of TMS ether **23** (488 mg, 1.32 mmol) in THF (14 mL). After 1 h of stirring at 0 °C, the mixture was partitioned between AcOEt (40 mL) and H_2_O (15 mL), and the aqueous layer was extracted with AcOEt (40 mL). The combined organic extracts were washed with brine (2 × 20 mL) and dried over anhydrous Na_2_SO_4_. Filtration and evaporation in vacuo furnished the crude product (634 mg), which was purified by column chromatography (silica gel 10 g, 10:1 → 3:1 *n*-hexane/AcOEt) to give α-hydroxyester **29** (383 mg, 98%) as a white solid. *R_f_* 0.45 (2:1 *n*-hexane/AcOEt); mp 142−143 °C (colorless needles from *n*-hexane); [α${]}_{D}^{21}$ + 25.0 (*c* 1.04, CHCl_3_); IR (KBr) 3455, 2959, 1761, 1732, 1466, 1375, 1236, 1144, 1098, 1045, 932 cm^−1^; ^1^H-NMR (500 MHz, CDCl_3_) δ 0.78 (d, *J* = 6.4 Hz, 3H, C8-C*H*_3_), 0.87 (dt, *J* = 13.0, 8.5 Hz, 1H, one of C1-*H*_2_), 1.22 (s, 3H, C10-C*H*_3_), 1.30 (s, 3H, C4-C*H*_3_), 1.44−1.71 (m, 5H, one of C1-*H*_2_, C2-*H*_2_, one of C3-*H*_2_, one of C7-*H*_2_), 2.15 (dt, *J* = 4.5, 13.4 Hz, 1H, one of C3-*H*_2_), 2.28 (dd, *J* = 6.3, 16.4 Hz, 1H, one of C7-*H*_2_), 2.32 (d, *J* = 4.6 Hz, 1H, C5-*H*), 2.53 (ddq, *J* = 6.3, 11.7, 6.4 Hz, 1H, C8-*H*), 3.33 (s, 1H, C9-O*H*), 3.82 (s, 3H, CO_2_C*H*_3_), 4.74 (dd, *J* = 4.6, 6.8 Hz, 1H, C6-*H*); ^13^C-NMR (125.7 MHz, CDCl_3_) δ 16.4 (CH_3_), 17.6 (CH_2_), 21.6 (CH_3_), 23.0 (CH_3_), 27.9 (CH_2_), 28.1 (CH_2_), 29.0 (CH), 30.7 (CH_2_), 38.9 (C), 43.4 (C), 44.7 (CH), 52.8 (CH_3_), 75.9 (CH), 80.8 (C), 175.9 (C), 183.6 (C); HRMS (ESI) *m*/*z* [M + Na]^+^ calcd for C_16_H_24_O_5_Na 319.1516; found 319.1519; Anal. Calcd for C_16_H_24_O_5_: C, 64.84; H, 8.16. Found: C, 64.82; H, 8.06.

*(2′R,2aS,5aS,6R,7R,8aR)-2a,5a,7-Trimethyloctahydrospiro[6H-naphtho[1,8-bc]furan-6,2′-oxiran]-2(2aH)-one* (**22**). Sodium bis(2-methoxyethoxy)aluminum hydride in toluene (3.3 M, 1.1 mL, 3.6 mmol) was diluted with CH_2_Cl_2_ (5.6 mL), and the solution was cooled to −78 °C. A solution of α-hydroxyester **29** (255 mg, 0.86 mmol) in CH_2_Cl_2_ (2 mL plus 2 × 0.5 mL rinse) was added, and the mixture was stirred at −23 °C for 22 h. The reaction was quenched by addition of MeOH (3 mL) followed by 10% aqueous potassium sodium tartrate (12 mL). After 3 h of stirring, the resulting mixture was extracted with AcOEt (2 × 40 mL), and the combined organic extracts were washed with brine (2 × 20 mL) and dried over anhydrous Na_2_SO_4_. Filtration and evaporation in vacuo furnished the crude product (235 mg), which was used without further purification.

This sequence was repeated employing bis(2-methoxyethoxy)aluminum hydride in toluene (3.3 M, 0.16 mL, 0.53 mmol) and CH_2_Cl_2_ (4 mL) with the reaction time of 16 h at −23 °C. The crude product (216 mg) was used without further purification.

A solution of crude diol **31** (216 mg) in THF (2 mL plus 1 mL and 2 × 0.5 mL rinse) was added dropwise to an ice-cooled (0 °C) suspension of NaH (60% in oil, 240 mg, 6.01 mmol) in THF (4.6 mL). After 30 min of stirring at 0 °C, *p*-toluenesulfonyl imidazole (577 mg, 2.59 mmol) was added, and the mixture was stirred at 0 °C for 11 h. The reaction was quenched with saturated aqueous NH_4_Cl (10 mL), and the resulting mixture was extracted with AcOEt (2 × 40 mL). The combined organic extracts were washed with brine (2 × 20 mL), and dried over anhydrous Na_2_SO_4_. Filtration and evaporation in vacuo furnished the crude product (563 mg), which was purified by column chromatography (silica gel 15 g, 40:1 → 10:1 *n*-hexane/AcOEt) to give epoxide **22** (182 mg, 84% for three steps) as a white solid. *R_f_* 0.53 (2:1 *n*-hexane/AcOEt); mp 83−84 °C (colorless needles from 8:1 *n*-hexane/Et_2_O); [α${]}_{D}^{21}$ +13.7 (*c* 1.21, CHCl_3_); IR (KBr) 2941, 1761, 1464, 1393, 1354, 1209, 1105, 997 cm^−1^; ^1^H-NMR (500 MHz, CDCl_3_) δ 0.76 (d, *J* = 6.6 Hz, 3H, C8-C*H*_3_), 0.97 (dt, *J* = 12.4, 8.5 Hz, 1H, one of C1-*H*_2_), 1.24 (s, 3H, C10-C*H*_3_), 1.30 (s, 3H, C4-C*H*_3_), 1.35 (dd, *J* = 10.1, 12.4 Hz, 1H, one of C1-*H*_2_), 1.45−1.51 (m, 2H, one of C2-*H*_2_, one of C3-*H*_2_), 1.66 (ddd, *J* = 4.7, 11.8, 16.0 Hz, 1H, one of C7-*H*_2_), 1.72 (m, 1H, one of C2-*H*_2_), 1.90 (d, *J* = 4.7 Hz, 1H, C5-*H*), 2.14 (dt, *J* = 5.4, 15.5 Hz, 1H, one of C3-*H*_2_), 2.37 (dd, *J* = 6.1, 16.0 Hz, 1H, one of C7-*H*_2_), 2.55 (ddq, *J* = 6.1, 11.8, 6.6 Hz, 1H, C8-*H*), 2.63 (d, *J* = 3.9 Hz, 1H, one of C11-*H*_2_), 2.71 (d, *J* = 3.9 Hz, 1H, one of C11-*H*_2_), 4.79 (br t, *J* = 4.7 Hz, 1H, C6-*H*); ^13^C-NMR (125.7 MHz, CDCl_3_) δ 15.0 (CH_3_), 17.3 (CH_2_), 22.4 (CH_3_), 24.9 (CH), 25.0 (CH_3_), 26.8 (CH_2_), 28.1 (C), 32.5 (CH_2_), 34.6 (C), 44.1 (C), 45.9 (CH_2_), 47.5 (CH), 63.7 (C), 76.0 (CH), 183.3 (C); HRMS (ESI) *m*/*z* [M + Na]^+^ calcd for C_15_H_22_O_3_Na 273.1461; found 273.1469.

*Marrubiin* (**1**). To a cooled (−10 °C) suspension of CuBr·SMe_2_ (65.1 mg, 0.316 mmol) in Et_2_O (1.5 mL) was added a 0.14 M solution of (3-furylmethyl)magnesium bromide (**32**) in Et_2_O (3.35 mL, 0.69 mmol) [prepared from 3-(bromomethyl)furan (563 mg, 3.37 mmol) and magnesium (103 mg, 4.22 mmol) in Et_2_O (4 mL) at 0 °C], followed by addition of a solution of epoxide **22** (40.2 mg, 0.161 mmol) in Et_2_O (0.5 mL plus 2 × 0.5 mL rinse). After 2 h, the reaction was quenched with saturated aqueous NH_4_Cl (5 mL), and the resulting mixture was extracted with AcOEt (3 × 10 mL). The combined organic extracts were washed with brine (20 mL), and dried over anhydrous Na_2_SO_4_. Filtration and evaporation in vacuo furnished the crude product (200 mg), which was chromatographed twice (silica gel 5 g, 20:1 → 10:1 *n*-hexane/AcOEt) to give marrubiin (**1**, 34.7 mg, 65%) and isomer **33** (4.7 mg, 9%) as white solids. *R_f_* 0.59 (1:1 *n*-hexane/AcOEt); mp 160−161 °C (colorless needles from 4:1 *n*-hexane/AcOEt) (lit. [38], mp 160 °C); [α${]}_{D}^{21}$ +34.4 (*c* 1.04, CHCl_3_) [lit. [38], [α${]}_{D}^{20}$ +35.8 (*c* 3.1, CHCl_3_)]; IR (KBr) 3466, 2940, 2870, 1740, 1468, 1395, 1356, 1304, 1269, 1200, 1153, 1101, 1024, 984 cm^−1^; ^1^H-NMR (500 MHz, CDCl_3_) δ 0.97 (d, *J* = 6.4 Hz, 3H, C8-C*H*_3_), 1.07 (s, 3H, C10-C*H*_3_), 1.26 (s, 1H, C9-O*H*), 1.29 (s, 3H, C4-C*H*_3_), 1.32 (dt, *J* = 12.8, 8.5 Hz, 1H, one of C1-*H*_2_), 1.43−1.55 (m, 2H, one of C2-*H*_2_, one of C3-*H*_2_), 1.66−1.79 (m, 4H, one of C1-*H*_2_, one of C2-*H*_2_, one of C7-*H*_2_, one of C11-*H*_2_), 1.90 (ddd, *J* = 7.2, 10.1, 14.4 Hz, 1H, one of C11-*H*_2_), 2.09−2.18 (m, 3H, one of C3-*H*_2_, one of C7-*H*_2_, C8-*H*), 2.23 (d, *J* = 4.6 Hz, 1H, C5-*H*), 2.48−2.58 (m, 2H, C12-*H*_2_), 4.74 (br dd, *J* = 4.6, 6.5 Hz, 1H, C6-*H*), 6.27 (s, 1H, C14-*H*), 7.24 (s, 1H, C16-*H*), 7.37 (s, 1H, C15-*H*); ^13^C-NMR (125.7 MHz, CDCl_3_) δ 16.6 (CH_3_), 18.2 (CH_2_), 21.0 (CH_2_), 22.3 (CH_3_), 22.9 (CH_3_), 28.3 (CH_2_), 28.6 (CH_2_), 31.5 (CH_2_), 32.4 (CH), 35.1 (CH_2_), 39.7 (C), 43.8 (C), 44.8 (CH), 75.8 (C), 76.2 (CH), 110.7 (CH), 125.0 (C), 138.6 (CH), 143.1 (CH), 183.8 (C); HRMS (ESI) *m*/*z* [M + Na]^+^ calcd for C_20_H_28_O_4_Na 355.1880; found 355.1867.

Data for (1*S*,4*R*,6*R*,7*R*,8*S*,12*R*)-7-[(3-methylfuran-2-yl)methyl]-7-hydroxy-1,6,8-trimethyl-2-oxo-3-oxatricyclo[6.3.1.0^4,12^]dodecane (**33**): *R_f_* 0.27 (3:1 *n*-hexane/AcOEt); mp 173−174 °C (colorless needles from 3:1 *n*-hexane/AcOEt); [α${]}_{D}^{22}$ +12.0 (*c* 0.38, CHCl_3_); IR (KBr) 3453, 2955, 2928, 2874, 1738, 1456, 1393, 1352, 1300, 1279, 1198, 1152, 1138, 999, 982 cm^−1^; ^1^H-NMR (500 MHz, CDCl_3_) δ 0.85 (d, *J* = 6.4 Hz, 3H, C8-C*H*_3_), 1.07 (s, 3H, C10-C*H*_3_), 1.15 (m, 1H, one of C1-*H*_2_), 1.28 (s, 3H, C4-C*H*_3_), 1.41−1.52 (m, 2H, one of C1-*H*_2_, one of C2-*H*_2_), 1.66−1.74 (m, 3H, one of C2-*H*_2_, one of C3-*H*_2_, one of C7-*H*_2_), 1.98 (s, 3H, C13-C*H*_3_), 2.06−2.21 (m, 3H, one of C3-*H*_2_, one of C7-*H*_2_, C8-*H*), 2.28 (d, *J* = 4.7 Hz, 1H, C5-*H*), 2.33 (s, 1H, C9-O*H*), 2.78 (d, *J* = 15.5 Hz, 1H, one of C11-*H*_2_), 2.88 (d, *J* = 15.5 Hz, 1H, one of C11-*H*_2_), 4.73 (ddd, *J* = 1.4, 4.7, 6.1 Hz, 1H, C6-*H*), 6.19 (d, *J* = 1.8 Hz, 1H, C14-*H*), 7.28 (d, *J* = 1.8 Hz, 1H, C15-*H*); ^13^C-NMR (125.7 MHz, CDCl_3_) δ 10.3 (CH_3_), 16.3 (CH_3_), 18.3 (CH_2_), 22.2 (CH_3_), 22.9 (CH_3_), 28.23 (CH_2_), 28.24 (CH_2_), 30.6 (CH_2_), 31.93 (CH), 31.94 (CH_2_), 39.6 (C), 43.8 (C), 44.6 (CH), 76.2 (CH), 76.9 (C), 113.3 (CH), 116.4 (C), 140.5 (CH), 148.0 (C), 183.9 (C); HRMS (ESI) *m*/*z* [M + Na]^+^ calcd for C_20_H_28_O_4_Na 355.1880; found 355.1871.

*(1S,4R,6R,7R,8S,12R)-7-[2-[2-(Trimethylsilyl)furan-3-yl]ethyl]]-7-hydroxy-1,6,8-trimethyl-2-oxo-3-oxatricyclo[6.3.1.0^4,12^]dodecane* (**35**). Phosphorus tribromide (0.28 mL, 2.98 mmol) was added to an ice-cooled (0 °C) solution of [2-(trimethylsilyl)furan-3-yl]methanol (998 mg, 5.86 mmol) in Et_2_O (30 mL). After 30 min of stirring, the reaction was quenched with brine (30 mL), and the resulting mixture was extracted with Et_2_O (30 mL). The organic extract was dried over anhydrous Na_2_SO_4_. Filtration and evaporation in vacuo furnished the pale brown oil, which was purified by distillation to give 3-(bromomethyl)-2-(trimethylsilyl)furan (618 mg, 45%) as a pale brown oil.

A solution of 3-(bromomethyl)-2-(trimethylsilyl)furan (584 mg, 2.50 mmol) in Et_2_O (1 mL) was added to a cooled (−10 °C) suspension of magnesium tuning (102 mg, 4.20 mmol) in Et_2_O (1 mL), and the reaction mixture was stirred for 1.5 h. The 0.23 M solution of [2-(trimethylsilyl)furan-3-yl]methylmagnesium bromide (**34**) in Et_2_O (1.5 mL, 0.345 mmol) thus obtained was added to a cooled (−10 °C) suspension of CuBr·SMe_2_ (69.6 mg, 0.339 mmol) in Et_2_O (0.6 mL), followed by addition of a solution of epoxide **22** (42.6 mg, 0.170 mmol) in Et_2_O (1.0 mL). After 4 h of stirring, the reaction was quenched with saturated aqueous NH_4_Cl (3 mL), and the resulting mixture was extracted with AcOEt (3 × 10 mL). The combined organic extracts were washed with brine (10 mL), and dried over anhydrous Na_2_SO_4_. Filtration and evaporation in vacuo furnished the pale brown oil (206 mg), which was purified by flash column chromatography (silica gel 4 g, *n*-hexane → 40:1 → 20:1 → 5:1 *n*-hexane/AcOEt) to give silylated marrubiin **35** (52.0 mg, 76%) as a colorless solid. *R_f_* 0.32 (3:1 *n*-hexane/AcOEt); [α${]}_{D}^{24}$ +29.3 (*c* 2.05, acetone); IR (neat) 3479, 3417, 2954, 2870, 1749, 1633, 1568, 1464, 1387, 1248, 1197, 1149, 1089, 1045, 1020, 991, 914, 840 cm^−^^1^; ^1^H-NMR (500 MHz, CDCl_3_) δ 0.29 (s, 9H, Si(C*H*_3_)_3_), 0.99 (d, *J* = 6.5 Hz, 3H, C8-C*H_3_*), 1.06 (s, 3H, C10-C*H*_3_), 1.29 (s, 3H, C4-C*H*_3_), 1.30 (m, 1H, one of C1-*H*_2_), 1.45 (m, 1H, one of C3-*H*_2_), 1.52 (m, 1H, one of C2-*H*_2_), 1.68−1.75 (m, 4H, one of C1-*H*_2_, one of C2-*H*_2_, one of C7-*H*_2_, one of C11-*H*_2_), 1.87 (ddd, *J* = 7.1, 10.3, 14.5 Hz, 1H, one of C11-*H*_2_), 2.09−2.19 (m, 3H, one of C3-*H*_2_, one of C7-*H*_2_, C8-*H*), 2.23 (d, *J* = 4.6 Hz, 1H, C5-*H*), 2.56−2.75 (m, 2H, C12-*H*_2_), 4.74 (dd, *J* = 4.6, 6.3 Hz, 1H, C6-*H*), 6.27 (d, *J* = 1.2 Hz, 1H, C14-*H*), 7.55 (d, *J* = 1.2 Hz, 1H, C15-*H*); ^13^C-NMR (125.7 MHz, CDCl_3_) δ −0.9 (CH_3_), 16.6 (CH_3_), 18.2 (CH_2_), 21.7 (CH_2_), 22.3 (CH_3_), 22.9 (CH_3_), 28.3 (CH_2_), 28.7 (CH_2_), 31.5 (CH_2_), 32.4 (CH), 36.2 (CH_2_), 39.7 (C), 43.8 (C), 44.9 (CH), 75.8 (C), 76.2 (CH), 110.7 (CH), 135.0 (C), 146.4 (CH), 154.3 (C), 183.9 (C); HRMS (ESI) *m*/*z* [M + Na]^+^ calcd for C_23_H_36_O_4_SiNa 427.2275; found 427.2259.

*Marrubiin* (**1**). Bu_4_NF in THF (1.0 M, 20 μL, 20 μmol) was added to a solution of silylated marrubiin **35** (0.7 mg, 1.7 μmol) in THF (0.2 mL). After 4 h of stirring, the mixture was partitioned between AcOEt (8 mL) and H_2_O (8 mL), and the aqueous layer was extracted with AcOEt (8 mL). The combined organic extracts were washed with brine (8 mL) and dried over anhydrous Na_2_SO_4_. Filtration and evaporation in vacuo furnished the colorless oil (0.5 mg), which was purified by column chromatography (silica gel 1 g, 4:1 → 1:1 *n*-hexane/AcOEt) to give marrubiin (**1**, 0.4 mg, 70%) as a white solid.

*(1S,4R,6R,7R,8S,12R)-7-[2-(2,5-Diethoxy-2,5-dihydrofuran-3-yl)ethyl]-7-hydroxy-1,6,8-trimethyl-2-oxo-3-oxatricyclo[6.3.1.0^4,12^]dodecane* (**36**). Pyridinium tribromide (11.0 mg, 34 μmol) was added to an ice-cooled (0 °C) solution of marrubiin (**1**, 10.4 mg, 31 μmol) in CH_2_Cl_2_/EtOH (1:1, 0.6 mL). After 10 min of stirring, the reaction was quenched with a mixture of saturated aqueous NaHCO_3_ (3 mL) and 1 M aqueous Na_2_S_2_O_3_ (2 mL), and the resulting mixture was extracted with AcOEt (2 × 20 mL). The combined organic extracts were washed with brine (30 mL), and dried over anhydrous Na_2_SO_4_. Filtration and evaporation in vacuo furnished the crude product (14.7 mg), which was purified by column chromatography (silica gel 2.5 g, 3:1 → 1:1 *n*-hexane/AcOEt) to give bisacetals **36** (10.4 mg, 79%, dr = 2:2:1:1) as a colorless oil. *R_f_* 0.58 (2:3 *n*-hexane/AcOEt); [α${]}_{D}^{23}$ +33.9 (*c* 1.19, CHCl_3_); IR (neat) 3516, 2972, 2930, 1767, 1755, 1458, 1373, 1346, 1198, 1101, 1045, 1018, 984 cm^−1^; ^1^H-NMR (500 MHz, CDCl_3_) δ 0.92 (d, *J* = 6.3 Hz, 3H, C8-C*H*_3_), 1.05 (s, 3H, C10-C*H*_3_), 1.21−1.34 (m, 11H), 1.43−1.56 (m, 2H), 1.66−1.77 (m, 4H), 1.86 (m, 1H, one of C*H*_2_), 2.05−2.32 (m, 6H), 3.53−3.82 (m, 4H, 2 × OC*H*_2_CH_3_), 4.74 (m, 1H, C6-*H*), 5.48 (s, 0.65H, C15-*H*), 5.60 (s, 0.65H, C16-*H*), 5.67 (s, 1H, C14-*H*), 5.75 (br d, *J* = 3.6 Hz, 0.35H, C16-*H*), 5.85 (br d, *J* = 3.6 Hz, 0.35H, C15-*H*); ^13^C-NMR (125.7 MHz, CDCl_3_) δ 15.3 (CH_3_), 15.4 (CH_3_), 16.50 (CH_3_), 16.51 (CH_3_), 16.54 (CH_3_), 18.09 (CH_2_), 18.12 (CH_2_), 22.22 (CH_3_), 22.23 (CH_3_), 22.26 (CH_3_), 22.28 (CH_3_), 22.31 (CH_2_), 22.4 (CH_2_), 22.49 (CH_2_), 22.52 (CH_2_), 22.87 (CH_3_), 22.89 (CH_3_), 22.96 (CH_3_), 22.97 (CH_3_), 28.27 (CH_2_), 28.28 (CH_2_), 28.59 (CH_2_), 28.61 (CH_2_), 28.63 (CH_2_), 31.4 (CH_2_), 31.46 (CH_2_), 31.47 (CH_2_), 32.0 (CH_2_), 32.11 (CH_2_), 32.15 (CH), 32.22 (CH), 32.29 (CH_2_), 32.33 (CH_2_), 32.5 (CH), 39.69 (C), 39.70 (C), 39.71 (C), 39.73 (C), 43.71 (C), 43.72 (C), 43.76 (C), 44.7 (CH), 44.8 (CH), 62.3 (CH_2_), 62.61 (CH_2_), 62.62 (CH_2_), 62.8 (CH_2_), 63.1 (CH_2_), 63.41 (CH_2_), 63.42 (CH_2_), 63.5 (CH_2_), 75.37 (C), 75.388 (C), 75.394 (C), 76.06 (CH), 76.12 (CH), 76.13 (CH), 105.95 (CH), 105.97 (CH), 106.937 (CH), 106.943 (CH), 107.5 (CH), 107.6 (CH), 108.2 (CH), 108.3 (CH), 123.8 (CH), 124.0 (CH), 124.1 (CH), 124.3 (CH), 145.3 (C), 145.78 (C), 145.81 (C), 183.7 (C), 183.8 (C); HRMS (ESI) *m*/*z* [M + Na]^+^ calcd for C_24_H_38_O_6_Na 445.2561; found 445.2550.

*[1S,4R,6R,7R,7(2S,3S,4R,5R),8S,12R]-7-[2-(2,5-Diethoxy-3,4-dihydroxytetrahydrofuran-3-yl)ethyl]-7-hydroxy-1,6,8-trimethyl-2-oxo-3-oxatricyclo[6.3.1.0^4,12^]dodecane* (**38**) *and [1S,4R,6R,7R,7(2R,3R,4S,5S),8S, 12R]-7-[2-(2,5-diethoxy-3,4-dihydroxytetrahydrofuran-3-yl)ethyl]-7-hydroxy-1,6,8-trimethyl-2-oxo-3-oxatri cyclo[6.3.1.0^4,12^]dodecane* (**39**). A 0.157 M solution of OsO_4_ in t-BuOH (0.06 mL, 9.4 μmol) was added to a solution of bisacetals **36** (21.7 mg, 51 μmol) and NMO (4.8 M in H_2_O, 0.04 mL, 0.19 mmol) in THF/H_2_O (10:1, 0.55 mL). After 1 h of stirring, the reaction was quenched with 1 M aqueous Na_2_S_2_O_3_ (5 mL), and the resulting mixture was extracted with AcOEt (2 × 15 mL). The combined organic extracts were washed with brine (15 mL), and dried over anhydrous Na_2_SO_4_. Filtration and evaporation in vacuo furnished the crude product (29.1 mg), which was purified by flash column chromatography (silica gel 2.3 g, 1:1 *n*-hexane/AcOEt) to give a 1:1 mixture of triols **38** and **39** (14.3 mg, 61%) as a colorless oil, along with recovered bisacetals **36** (8.2 mg, 38%) as a colorless oil. *R_f_* 0.49 (1:4 *n*-hexane/AcOEt); [α${]}_{D}^{21}$ +29.1 (*c* 0.56, CHCl_3_); IR (neat) 3462, 2930, 1751, 1458, 1389, 1375, 1101, 978 cm^−1^; ^1^H-NMR (500 MHz, CDCl_3_) δ 0.91 (d, *J* = 6.5 Hz, 1.5H, C8-C*H*_3_), 0.92 (d, *J* = 6.4 Hz, 1.5H, C8-C*H*_3_), 1.04 (s, 3H, C10-C*H*_3_), 1.207 (t, *J* = 7.1 Hz, 1.5H, OCH_2_C*H*_3_), 1.214 (t, *J* = 7.1 Hz, 1.5H, OCH_2_C*H*_3_), 1.23 (t, *J* = 7.1 Hz, 3H, OCH_2_C*H*_3_), 1.29 (s, 3H, C4-C*H*_3_), 1.42−1.52 (m, 2H, C*H*_2_), 1.60−1.97 (m, 8H, 4 × C*H*_2_), 2.03−2.15 (m, 3.5H, C*H*_2_, C8-*H*, O*H*), 2.24 (d, *J* = 4.7 Hz, 1H, C5-*H*), 2.33 (br s, 0.5H, O*H*), 2.99 (d, *J* = 6.9 Hz, 0.5H, O*H*), 3.05 (m, 1H, O*H*), 3.44−3.57 (m, 2.5H, OC*H*_2_CH_3_, O*H*), 3.76−3.84 (m, 2H, OC*H*_2_CH_3_), 3.96 (m, 1H, C14-*H*), 4.73 (br s, 1H, C6-*H*), 4.85 (s, 0.5H, C16-*H*), 4.86 (s, 0.5H, C16-*H*), 4.97 (d, *J* = 4.3 Hz, 0.5H, C15-*H*), 4.98 (d, *J* = 4.3 Hz, 0.5H, C15-*H*); ^13^C-NMR (125.7 MHz, CDCl_3_) δ 14.88 (CH_3_), 14.93 (CH_3_), 15.2 (CH_3_), 16.5 (CH_3_), 16.8 (CH_3_), 18.1 (CH_2_), 22.2 (CH_3_), 22.3 (CH_3_), 22.85 (CH_3_), 22.93 (CH_3_), 27.4 (CH_2_), 28.1 (CH_2_), 28.27 (CH_2_), 28.28 (CH_2_), 28.31 (CH_2_), 28.4 (CH_2_), 28.5 (CH_2_), 28.6 (CH_2_), 31.5 (CH_2_), 31.6 (CH_2_), 32.47 (CH), 32.49 (CH), 39.97 (C), 40.03 (C), 43.88 (C), 43.91 (C), 44.90 (CH), 44.93 (CH), 63.07 (CH_2_), 63.10 (CH_2_), 64.55 (CH_2_), 64.57 (CH_2_), 75.30 (C), 75.33 (C), 76.3 (CH), 76.4 (CH), 80.3 (CH), 80.4 (CH), 81.2 (C), 81.3 (C), 106.5 (CH), 106.7 (CH), 109.17 (CH), 109.19 (CH), 184.10 (C), 184.12 (C); HRMS (ESI) *m*/*z* [M + Na]^+^ calcd for C_24_H_40_O_8_Na 479.2615; found 479.2631.

*Marrulibacetal* (**13**). TsOH (2.9 mg, 17 μmol) was added to a mixture of triols **38** and **39** (12.5 mg, 27 μmol) in benzene (1 mL), and the mixture was stirred for 1.5 h. The reaction was quenched with saturated aqueous NaHCO_3_ (5 mL), and the resulting mixture was extracted with AcOEt (2 × 15 mL). The combined organic extracts were washed with brine (15 mL), and dried over anhydrous Na_2_SO_4_. Filtration and evaporation in vacuo furnished the crude product (19.2 mg), which was purified by flash column chromatography (silica gel 2 g, 2:1 *n*-hexane/AcOEt) to give a mixture of marrulibacetal (**13**) and its diastereomers. The mixture was flash chromatographed (silica gel 4.5 g, 30:1 CHCl_3_/ acetone) to provide a mixture of marrulibacetal (**13**) and C13,14,15,16-epimer **41**, along with a 1:1 mixture of C15-epimer and C13,14,16-epimer (2.0 mg, 18%). Separation of marrulibacetal (**13**) and **41** by flash column chromatography (silica gel 4.5 g, 50:1 CHCl_3_/acetone) yielded marrulibacetal (**13**, 4.1 mg, 36%) and C13,14,15,16-epimer **41** (4.3 mg, 38%) as white solids. *R_f_* 0.44 (4:1 CH_2_Cl_2_/acetone); mp 177−179 °C (colorless needles from *n*-hexane/benzene); [α${]}_{D}^{27}$ −21.7 (*c* 1.16, CHCl_3_) [lit. [12], [α${]}_{D}^{25}$ −13.1 (*c* 0.29, CHCl_3_)]; IR (neat) 3435, 2961, 2928, 1773, 1740, 1458, 1389, 1244, 1200, 1111, 1053, 935 cm^−1^; ^1^H-NMR (500 MHz, CDCl_3_) δ 1.03 (s, 3H, C10-C*H*_3_), 1.11 (d, *J* = 6.8 Hz, 3H, C8-C*H*_3_), 1.19 (m, 1H, one of C1-*H*_2_), 1.22 (t, *J* = 7.1 Hz, 3H, OCH_2_C*H*_3_), 1.28 (s, 3H, C4-C*H*_3_), 1.42 (m, 1H, one of C3-*H*_2_), 1.49 (m, 1H, one of C2-*H*_2_), 1.71−1.78 (m, 2H, one of C2-*H*_2_, one of C11-*H*_2_), 1.80−1.90 (m, 3H, one of C7-*H*_2_, one of C11-*H*_2_, one of C12-*H*_2_), 1.98 (m, 1H, one of C1-*H*_2_), 2.05−2.20 (m, 4H, one of C3-*H*_2_, one of C7-*H*_2_, C8-*H*, one of C12-*H*_2_), 2.36 (d, *J* = 4.7 Hz, 1H, C5-*H*), 2.60 (s, 1H, C13-O*H*), 2.61 (d, *J* = 6.2 Hz, 1H, C14-O*H*), 3.51 (dq, *J* = 9.5, 7.1 Hz, 1H, one of OC*H*_2_CH_3_), 3.82 (dq, *J* = 9.5, 7.1 Hz, 1H, one of OC*H*_2_CH_3_), 3.95 (dd, *J* = 2.0, 6.2 Hz, 1H, C14-*H*), 4.77 (br dd, *J* = 4.7, 5.9 Hz, 1H, C6-*H*), 5.04 (d, *J* = 2.0 Hz, 1H, C15-*H*), 5.46 (s, 1H, C16-*H*); ^13^C-NMR (125.7 MHz, CDCl_3_) δ 15.0 (CH_3_), 17.9 (CH_2_), 19.5 (CH_3_), 21.1 (CH_2_), 22.1 (CH_3_), 23.1 (CH_3_), 27.8 (CH_2_), 28.2 (CH_2_), 29.6 (CH_2_), 32.3 (CH_2_), 33.6 (CH), 40.9 (C), 43.9 (C), 44.6 (CH), 63.9 (CH_2_), 75.6 (C), 76.5 (CH), 78.5 (CH), 80.4 (C), 105.3 (CH), 108.7 (CH), 184.0 (C); HRMS (EI) *m*/*z* [M^+^] calcd for C_22_H_34_O_7_ 410.2305; found 410.2300.

Data for (2*S*,2′a*S*,3*S*,3a*R*,5′a*S*,6*R*,7′*R*,7a*R*,8′a*R*,8′b*R*)-2-ethoxy-3,3a-dihydroxy-2′a,5′a,7′-trimethyltetradecahydrospiro[6*H*-furo[2,3-*b*]pyran-6,6′-[6*H*]naphtho[1,8-*bc*]furan]-2′(2′a*H*)-one (**41**). *R_f_* 0.56 (4:1 CH_2_Cl_2_/acetone); [α${]}_{D}^{28}$ +57.2 (*c* 1.26, CHCl_3_); IR (KBr) 3458, 2930, 1771, 1749, 1466, 1389, 1260, 1198, 1153, 1094, 1063, 1040, 989, 941 cm^−1^; ^1^H-NMR (500 MHz, CDCl_3_) δ 0.96 (d, *J* = 6.1 Hz, 3H, C8-C*H*_3_), 1.01 (s, 3H, C10-C*H*_3_), 1.21 (t, *J* = 7.1 Hz, 3H, OCH_2_C*H*_3_), 1.30 (s, 3H, C4-C*H*_3_), 1.39−1.54 (m, 4H, one of C1-*H*_2_, one of C2-*H*_2_, one of C3-*H*_2_, one of C11-*H*_2_), 1.70−1.83 (m, 4H, one of C1-*H*_2_, one of C2-*H*_2_, one of C7-*H*_2_, one of C12-*H*_2_), 1.95−2.11 (m, 4H, one of C3-*H*_2_, one of C7-*H*_2_, C8-*H*, one of C11-*H*_2_), 2.20 (m, 1H, one of C12-*H*_2_), 2.34 (d, *J* = 4.5 Hz, 1H, C5-*H*), 2.75 (d, *J* = 4.8 Hz, 1H, C14-O*H*), 2.79 (s, 1H, C13-O*H*), 3.46 (dq, *J* = 9.5, 7.1 Hz, 1H, one of OC*H*_2_CH_3_), 3.74 (d, *J* = 4.8 Hz, 1H, C14-*H*), 3.86 (dq, *J* = 9.5, 7.1 Hz, 1H, one of OC*H*_2_CH_3_), 4.78 (br dd, *J* = 4.5, 7.7 Hz, 1H, C6-*H*), 5.01 (s, 1H, C15-*H*), 5.50 (s, 1H, C16-*H*); ^13^C-NMR (125.7 MHz, CDCl_3_) δ 14.8 (CH_3_), 16.1 (CH_3_), 18.1 (CH_2_), 21.5 (CH_2_), 23.2 (CH_3_), 23.9 (CH_3_), 28.3 (CH_2_), 30.4 (CH_2_), 30.9 (CH_2_), 31.3 (CH_2_), 35.0 (CH), 40.8 (C), 43.9 (C), 45.5 (CH), 63.7 (CH_2_), 75.9 (C), 76.7 (CH), 80.0 (CH), 80.5 (C), 106.1 (CH), 108.2 (CH), 183.9 (C); HRMS (EI) *m*/*z* [M^+^] calcd for C_22_H_34_O_7_ 410.2305; found 410.2297.

*[1S,4R,6R,7R,7(2S,3S,4R,5R),8S,12R]-7-[2-(3,4-Dihydroxy-2,5-dimethoxytetrahydrofuran-3-yl)ethyl]-7-hydroxy-1,6,8-trimethyl-2-oxo-3-oxatricyclo[6.3.1.0^4,12^]dodecane* (**40**) *and desertine* (**18**). Pyridinium tribromide (32.9 mg, 0.102 mmol) was added to an ice-cooled (0 °C) solution of marrubiin (**1**, 30.5 mg, 91.7 μmol) in CH_2_Cl_2_/MeOH (1:1, 1.8 mL). After 20 min of stirring, the reaction was quenched with saturated aqueous NaHCO_3_ (5 mL), and the resulting mixture was extracted with AcOEt (2 × 15 mL). The combined organic extracts were washed with brine (2 × 10 mL), and dried over anhydrous Na_2_SO_4_. Filtration and evaporation in vacuo furnished the yellow oil (53.1 mg), which was purified by column chromatography (silica gel 3 g, 4:1 → 2:1 → 1:1 *n*-hexane/AcOEt) to give bisacetals **37** (33.1 mg, 91%, dr = 2:2:1:1) as a yellow oil.

A 0.157 M solution of OsO_4_ in *t*-BuOH (0.08 mL, 12.5 μmol) was added to a solution of bisacetals **37** (28.3 mg, 71.7 μmol) and NMO (4.8 M in H_2_O, 0.06 mL, 0.29 mmol) in THF/H_2_O (1:1, 0.8 mL). After 1 h of stirring, the reaction was quenched with 1 M aqueous Na_2_S_2_O_3_ (5 mL), and the resulting mixture was extracted with AcOEt (2 × 15 mL). The combined organic extracts were washed with brine (2 × 10 mL), and dried over anhydrous Na_2_SO_4_. Filtration and evaporation in vacuo furnished the brown oil (40.7 mg), which was purified by column chromatography (silica gel 3.1 g, 2:1 → 1:1 *n*-hexane/AcOEt) to give a 1:1 mixture of desertine (**18**) and its diastereomer **40** (20.2 mg, 66%) as a brown oil, along with recovered bisacetals **37** (8.8 mg, 29%) as a colorless oil. *R_f_* 0.30 (1:1 *n*-hexane/AcOEt); [α${]}_{D}^{20}$ +12.9 (*c* 1.02, CHCl_3_); IR (neat) 3464, 2951, 1748, 1454, 1391, 1258, 1198, 1148, 1101, 1043, 989 cm^−1^; ^1^H-NMR (500 MHz, CDCl_3_) δ 0.91 (d, *J* = 6.5 Hz, 3H, C8-C*H*_3_), 1.04 (s, 1.5H, C10-C*H*_3_), 1.05 (s, 1.5H, C10-C*H*_3_), 1.28 (m, 1H, one of C1-*H*_2_), 1.29 (s, 3H, C4-C*H*_3_), 1.45 (m, 1H, one of C3-*H*_2_), 1.51 (m, 1H, one of C2-*H*_2_), 1.65 (m, 1H, one of C7-*H*_2_), 1.60−1.98 (m, 6H, one of C1-*H*_2_, one of C2-*H*_2_, C11-*H*_2_, C12-*H*_2_), 2.07 (m, 1H, C8-*H*), 2.11 (m, 1H, one of C3-*H*_2_), 2.14 (m, 1H, one of C7-*H*_2_), 2.22 (d, *J* = 4.2 Hz, 0.5H, C5-*H*), 2.23 (d, *J* = 4.2 Hz, 0.5H, C5-*H*), 3.40 (s, 3H, C16-OC*H*_3_), 3.47 (s, 3H, C15-OC*H*_3_), 3.92 (d, *J* = 3.5 Hz, 0.5H, C14-*H*), 3.95 (d, *J* = 3.5 Hz, 0.5H, C14-*H*), 4.73 (m, 1H, C6-*H*), 4.76 (s, 0.5H, C16-*H*), 4.77 (s, 0.5H, C16-*H*), 4.89 (d, *J* = 3.5 Hz, 0.5H, C15-*H*), 4.90 (d, *J* = 3.5 Hz, 0.5H, C15-*H*); ^13^C-NMR (125.7 MHz, CDCl_3_) δ 16.4 (CH_3_), 16.8 (CH_3_), 18.1 (CH_2_), 22.2 (CH_3_), 22.3 (CH_3_), 22.86 (CH_3_), 22.94 (CH_3_), 27.4 (CH_2_), 28.0 (CH_2_), 28.2 (CH_2_), 28.26 (CH_2_), 28.28 (CH_2_), 28.41 (CH_2_), 28.43 (CH_2_), 28.5 (CH_2_), 31.5 (CH_2_), 31.6 (CH_2_), 32.5 (CH), 32.6 (CH), 40.0 (C), 40.1 (C), 43.87 (C), 43.91 (C), 44.9 (CH), 45.0 (CH), 55.08 (CH_3_), 55.11 (CH_3_), 56.40 (CH_3_), 56.42 (CH_3_), 75.3 (C), 75.4 (C), 76.2 (CH), 76.3 (CH), 80.2 (CH), 80.3 (CH), 81.2 (C), 81.4 (C), 108.3 (CH), 108.6 (CH), 110.7 (CH), 110.8 (CH), 183.95 (C), 183.99 (C); HRMS (ESI) *m*/*z* [M + Na]^+^ calcd for C_22_H_36_O_8_Na 451.2302; found 451.2308.

*Marrulibacetal A* (**17**). TsOH (8.6 mg, 50 μmol) was added to a mixture of desertine (**18**) and its diastereomer **40** (29.6 mg, 69.0 μmol) in benzene (1.4 mL). After 5 h of stirring, an additional portion of TsOH (1.6 mg, 9.3 μmol) was added, and the reaction mixture was stirred for 3.5 h. The reaction was quenched with saturated aqueous NaHCO_3_ (2 mL), and the resulting mixture was extracted with AcOEt (3 × 5 mL). The combined organic extracts were washed with brine (5 mL), and dried over anhydrous Na_2_SO_4_. Filtration and evaporation in vacuo furnished the brown oil (68.1 mg), which was purified by flash column chromatography (silica gel 5 g, 1:1 *n*-hexane/AcOEt) to give marrulibacetal A (**17**, 4.3 mg, 16%) and C13,14,15,16-isomer **42** (6.2 mg, 23%) as colorless amorphous solids. *R_f_* 0.73 (AcOEt); [α${]}_{D}^{21}$ −14.0 (*c* 1.69, CHCl_3_) (lit. [14], [α${]}_{D}^{25}$ −10.77); IR (neat) 2953, 2928, 1769, 1748, 1456, 1259, 1198, 1117, 1053, 1015, 989, 935 cm^−^^1^; ^1^H-NMR (500 MHz, CDCl_3_) δ 1.03 (s, 3H, C10-C*H*_3_), 1.11 (d, *J* = 7.0 Hz, 3H, C8-C*H*_3_), 1.19 (m, 1H, one of C1-*H*_2_), 1.28 (s, 3H, C4-C*H*_3_), 1.42 (m, 1H, one of C3-*H*_2_), 1.49 (m, 1H, one of C2-*H*_2_), 1.71−1.92 (m, 5H, one of C2-*H*_2_, one of C7-*H*_2_, C11-*H*_2_, one of C12-*H*_2_), 1.96 (t, *J* = 10.6 Hz, 1H, one of C1-*H*_2_), 2.05−2.15 (m, 3H, one of C3-*H*_2_, C8-*H*, one of C12-*H*_2_), 2.20 (dd, *J* = 5.5, 16.0 Hz, 1H, one of C7-*H*_2_), 2.38 (d, *J* = 4.5 Hz, 1H, C5-*H*), 3.42 (s, 3H, OC*H*_3_), 3.89 (d, *J* = 1.2 Hz, 1H, C14-*H*), 4.79 (dd, *J* = 4.5, 6.3 Hz, 1H, C6-*H*), 4.93 (d, *J* = 1.2 Hz, 1H, C15-*H*), 5.48 (s, 1H, C16-*H*); ^13^C-NMR (125.7 MHz, CDCl_3_) δ 17.9 (CH_2_), 19.4 (CH_3_), 20.7 (CH_2_), 22.0 (CH_3_), 23.1 (CH_3_), 27.8 (CH_2_), 28.2 (CH_2_), 30.0 (CH_2_), 32.2 (CH_2_), 33.5 (CH), 40.9 (C), 43.9 (C), 44.6 (CH), 55.5 (CH_3_), 75.8 (C), 76.5 (CH), 78.6 (CH), 80.3 (C), 105.7 (CH), 109.8 (CH), 184.1 (C); HRMS (ESI) *m*/*z* [M + Na]^+^ calcd for C_21_H_32_O_7_Na 419.2045; found 419.2037.

Data for (2*S*,2′a*S*,3*S*,3a*R*,5′a*S*,6*R*,7′*R*,7a*R*,8′a*R*,8′b*R*)-3,3a-dihydroxy-2-methoxy-2′a,5′a,7′-trimethyl tetradecahydrospiro[6*H*-furo[2,3-*b*]pyran-6,6′-[6*H*]naphtho[1,8-*bc*]furan]-2′(2′a*H*)-one (**42**): *R_f_* 0.78 (AcOEt); [α${]}_{D}^{22}$ +49.1 (*c* 0.75, CHCl_3_); IR (neat) 2955, 2928, 1749, 1541, 1506, 1456, 1265 cm^−^^1^; ^1^H-NMR (500 MHz, CDCl_3_) δ 0.94 (d, *J* = 6.1 Hz, 3H, C8-C*H*_3_), 1.01 (s, 3H, C10-C*H*_3_), 1.31 (s, 3H, C4-C*H*_3_), 1.42−1.56 (m, 4H, one of C1-*H*_2_, one of C2-*H*_2_, one of C3-*H*_2_, one of C11-*H*_2_), 1.70−1.83 (m, 4H, one of C1-*H*_2_, one of C2-*H*_2_, one of C7-*H*_2_, one of C12-*H*_2_), 1.98 (dt, *J* = 3.8, 14.2 Hz, 1H, one of C11-*H*_2_), 1.99−2.12 (m, 3H, one of C3-*H*_2_, one of C7-*H*_2_, C8-*H*), 2.17 (dt, *J* = 4.4, 14.2 Hz, 1H, one of C12-*H*_2_), 2.35 (d, *J* = 4.5 Hz, 1H, C5-*H*), 2.87 (br s, 1H, O*H*), 3.41 (s, 3H, OC*H*_3_), 3.73 (s, 1H, C14-*H*), 4.79 (dd, *J* = 4.5, 6.2 Hz, 1H, C6-*H*), 4.91 (s, 1H, C15-*H*), 5.52 (s, 1H, C16-*H*); ^13^C-NMR (125.7 MHz, CDCl_3_) δ 16.0 (CH_3_), 18.1 (CH_2_), 21.4 (CH_2_), 23.1 (CH_3_), 24.0 (CH_3_), 28.3 (CH_2_), 30.6 (CH_2_), 30.9 (CH_2_), 31.3 (CH_2_), 35.0 (CH), 40.8 (C), 43.9 (C), 45.5 (CH), 55.2 (CH_3_), 76.1 (C), 76.7 (CH), 79.8 (CH), 80.5 (C), 106.3 (CH), 109.4 (CH), 184.1 (C); HRMS (ESI) *m*/*z* [M + Na]^+^ calcd for C_21_H_32_O_7_Na 419.2045; found 419.2059.

*Marrubasch F* (**19**). *m*-CPBA (ca. 70%, 10.7 mg, 43.4 μmol) was azeotropically dried with benzene, and dissolved in CH_2_Cl_2_ (0.2 mL). The *m*-CPBA solution was added to an ice-cooled (0 °C) solution of silylated marrubiin **35** (8.1 mg, 20.0 μmol) in CH_2_Cl_2_ (0.8 mL). After 1.5 h of stirring, the reaction was quenched with a mixture of 1 M aqueous Na_2_S_2_O_3_ (2 mL) and saturated aqueous NaHCO_3_ (2 mL), and the resulting mixture was extracted with AcOEt (10 mL). The organic extract was washed with brine (4 mL), and dried over anhydrous Na_2_SO_4_. Filtration and evaporation in vacuo furnished the colorless oil (28.4 mg), which was purified by preparative thin layer chromatography (200 mm × 100 mm × 0.25 mm preparative silica gel plate and elution with 1:1 *n*-hexane/AcOEt) to give marrubasch F (**19**, 3.9 mg, 56%) as a white solid. *R_f_* 0.32 (1:1 *n*-hexane/AcOEt); mp 195−196 °C (pale yellow plates from 1:2 *n*-hexane/AcOEt) (lit. [20], mp 191−193 °C); [α${]}_{D}^{21}$ +41.1 (*c* 0.534, CHCl_3_) [lit. [20], [α]_D_ + 41.5 (*c* 1.00, CHCl_3_)]; IR (neat) 3536, 2926, 2873, 1747, 1456, 1199, 1139, 1095, 1076, 1041, 983 cm^−1^; ^1^H-NMR (500 MHz, CDCl_3_) δ 0.96 (d, *J* = 6.4 Hz, 3H, C8-C*H*_3_), 1.04 (s, 3H, C10-C*H*_3_), 1.25 (dt, *J* = 12.8, 8.5 Hz, 1H, one of C1-*H*_2_), 1.30 (s, 3H, C4-C*H*_3_), 1.45 (dt, *J* = 14.4, 4.8 Hz, 1H, one of C3-*H*_2_), 1.53 (m, 1H, one of C2-*H*_2_), 1.69−1.84 (m, 5H, one of C1-*H*_2_, one of C2-*H*_2_, one of C7-*H*_2_, C11-*H*_2_), 2.05−2.17 (m, 3H, one of C3-*H*_2_, one of C7-*H*_2_, C8-*H*), 2.28 (d, *J* = 4.5 Hz, 1H, C5-*H*), 2.41−2.52 (m, 2H, C12-*H*_2_), 4.75 (dd, *J* = 4.5, 6.5 Hz, 1H, C6-*H*), 4.80 (br d, *J* = 1.4 Hz, 2H, C15-*H*_2_), 7.14 (t, *J* = 1.4 Hz, 1H, C14-*H*); ^13^C-NMR (125.7 MHz, CDCl_3_) δ 16.7 (CH_3_), 18.1 (CH_2_), 21.2 (CH_2_), 22.3 (CH_3_), 22.9 (CH_3_), 28.3 (CH_2_), 28.6 (CH_2_), 31.6 (CH_2_), 32.4 (CH_2_), 32.6 (CH_2_), 39.9 (C), 43.8 (C), 44.9 (CH), 70.4 (CH_2_), 75.2 (C), 76.2 (CH), 134.7 (C), 144.3 (CH), 174.9 (C), 183.9 (C); HRMS (ESI) *m/z* [M + Na]^+^ calcd for C_20_H_28_O_5_Na 371.1830; found 371.1834.

*(1S,4R,6R,7R,8S,12R)-7-Hydroxy-1,6,8-trimethyl-2-oxo-7-propargyl-3-oxatricyclo[6.3.1.0^4,12^]dodecane* (**43**). Lithium acetylide ethylene diamine complex (36.5 mg, 0.397 mmol) was added to a solution of epoxide **22** (10.7 mg, 42.7 μmol) in DMSO (0.2 mL). After 30 min of stirring, the reaction was quenched with H_2_O (1 mL), and the resulting mixture was extracted with AcOEt (3 × 5 mL). The combined organic extracts were washed with brine (2 × 5 mL), and dried over anhydrous Na_2_SO_4_. Filtration and evaporation in vacuo furnished the pale yellow solid (13.1 mg), which was purified by column chromatography (silica gel 3 g, 2:1 *n*-hexane/AcOEt) to give alcohol **43** (11.2 mg, 95%) as a pale yellow solid. *R_f_* 0.57 (1:1 *n*-hexane/AcOEt); mp 195−196 °C (colorless plates from *n*-hexane/Et_2_O); [α${]}_{D}^{25}$ +29.9 (*c* 0.249, CHCl_3_); IR (neat) 3306, 3019, 2955, 2934, 2872, 1757, 1458, 1427, 1393, 1136, 1096, 1045, 1003 cm^−^^1^; ^1^H-NMR (500 MHz, CDCl_3_) δ 0.99 (d, *J* = 6.3 Hz, 3H, C8-C*H*_3_), 1.05 (s, 3H, C10-C*H*_3_), 1.25 (dt, *J* = 13.0, 9.0 Hz, 1H, one of C1-*H*_2_), 1.30 (s, 3H, C4-C*H*_3_), 1.46 (dt, *J* = 14.7, 4.2 Hz, 1H, one of C3-*H*_2_), 1.53 (m, 1H, one of C2-*H*_2_), 1.69 (ddd, *J* = 6.3, 13.3, 17.7 Hz, 1H, one of C7-*H*_2_), 1.76 (m, 1H, one of C2-*H*_2_), 1.85 (dd, *J* = 9.6, 11.6 Hz, 1H, one of C1-*H*_2_), 2.09−2.18 (m, 3H, one of C3-*H*_2_, one of C7-*H*_2_, C8-*H*), 2.19 (t, *J* = 2.7 Hz, 1H, C13-*H*), 2.26 (d, *J* = 4.5 Hz, 1H, C5-*H*), 2.36 (dd, *J* = 2.7, 17.1 Hz, 1H, one of C11-*H*_2_), 2.59 (dd, *J* = 2.7, 17.1 Hz, 1H, one of C11-*H*_2_), 4.73 (dd, *J* = 4.5, 6.3 Hz, 1H, C6-*H*); ^13^C-NMR (125.7 MHz, CDCl_3_) δ 16.3 (CH_3_), 18.1 (CH_2_), 22.3 (CH_3_), 22.6 (CH_3_), 25.3 (CH_2_), 28.21 (CH_2_), 28.22 (CH_2_), 31.5 (CH_2_), 32.3 (CH), 39.2 (C), 43.9 (C), 44.8 (CH), 73.77 (C), 73.79 (CH), 76.1 (CH), 80.7 (C), 183.8 (C); HRMS (ESI) *m/z* [M + Na]^+^ calcd for C_17_H_24_O_3_Na 299.1618; found 299.1623.

*Cyllenine C* (**12**). To an ice-cooled (0 °C) mixture of alcohol **43** (6.8 mg, 24.6 μmol) and *m*-CPBA (ca. 65%, 9.8 mg, 36.9 μmol) in CH_2_Cl_2_ (0.3 mL) was added a 0.123 M solution of methanesulfonic acid in CH_2_Cl_2_ (0.20 mL, 24.6 μmol) followed by Ph_3_PAuNTf_2_ (1.0 mg, 1.3 μmol). After 1 h of stirring at room temperature, the reaction was quenched with saturated aqueous NaHCO_3_ (1 mL), and the resulting mixture was extracted with AcOEt (2 × 5 mL). The combined organic extracts were washed with brine (5 mL), and dried over anhydrous Na_2_SO_4_. Filtration and evaporation in vacuo furnished the colorless oil (13.7 mg), which was purified by column chromatography (silica gel 2 g, 2:1 *n*-hexane/AcOEt) to give cyllenine C (**12**, 6.9 mg, 96%) as a white solid. *R_f_* 0.32 (1:2 *n*-hexane/AcOEt); mp 164−165 °C (colorless needles from *n*-hexane/Et_2_O); [α${]}_{D}^{24}$ +22.1 (*c* 0.69, CH_2_Cl_2_) [lit. [11], [α${]}_{D}^{20}$ +11.82 (*c* 0.33, CH_2_Cl_2_)]; IR (neat) 3019, 2934, 2870, 1763, 1474, 1458, 1420, 1391, 1273, 1120, 1067, 1042, 993 cm^−^^1^; ^1^H-NMR (500 MHz, CDCl_3_) δ 0.93 (d, *J* = 6.5 Hz, 3H, C8-C*H_3_*), 1.09 (s, 3H, C10-C*H_3_*), 1.30 (dt, *J* = 12.2, 9.8 Hz, 1H, one of C1-*H_2_*), 1.31 (s, 3H, C4-C*H_3_*), 1.46 (m, 1H, one of C1-*H*_2_), 1.49 (m, 1H, one of C3-*H*_2_), 1.52 (m, 1H, one of C2-*H*_2_), 1.72 (ddd, *J* = 6.3, 11.9, 16.4 Hz, 1H, one of C7-*H_2_*), 1.78 (m, 1H, one of C2-*H_2_*), 1.95 (ddd, *J* = 4.4, 11.4, 13.8 Hz, 1H, one of C11-*H_2_*), 2.12 (m, 1H, one of C3-*H_2_*), 2.18 (ddq, *J* = 6.3, 11.9, 6.5 Hz, 1H, C8-*H*), 2.19 (d, *J* = 4.5 Hz, 1H, C5-*H*), 2.22 (ddd, *J* = 8.9, 11.5, 13.8 Hz, 1H, one of C11-*H_2_*), 2.28 (dd, *J* = 6.3, 16.4 Hz, 1H, one of C7-*H_2_*), 2.54 (ddd, *J* = 4.4, 11.5, 18.8 Hz, 1H, one of C12-*H_2_*), 2.62 (ddd, *J* = 8.9, 11.4, 18.8 Hz, 1H, one of C12-*H_2_*), 4.75 (dd, *J* = 4.5, 6.3 Hz, 1H, C6-*H*); ^13^C-NMR (125.7 MHz, CDCl_3_) δ 15.4 (CH_3_), 17.7 (CH_2_), 22.2 (CH_3_), 22.4 (CH_3_), 24.5 (CH_2_), 27.7 (CH_2_), 28.2 (CH_2_), 29.3 (CH_2_), 31.2 (CH_2_), 32.3 (CH), 38.6 (C), 44.0 (C), 45.3 (CH), 75.6 (CH), 91.2 (C), 176.9 (C), 183.3 (C); HRMS (ESI) *m*/*z* [M + Na]^+^ calcd for C_17_H_24_O_4_Na 315.1567; found 315.1566.

*(1S,4R,6R,7R,8S,12R)-7-[4-(tert-Butyldimethylsilyl)oxybutyl]-7-hydroxy-1,6,8-trimethyl-2-oxo-3-oxatricyclo[6.3.1.0^4,12^]dodecane* (**45**). To an ice-cooled (0 °C) solution of 3-(*tert*-butyldimethylsilyl)oxypropylmagnesium bromide (**44**) [prepared from (3-bromopropoxy)(*tert*-butyl)dimethylsilane (2.00 g, 7.90 mmol) and magnesium tuning (241 mg, 9.93 mmol)] in THF (14 mL) was added CuBr·SMe_2_ (129 mg, 0.627 mmol), followed by addition of a solution of epoxide **22** (39.1 mg, 0.156 mmol) in THF (0.20 mL plus 2 × 0.20 mL rinse). After 5 h of stirring at room temperature, the reaction was quenched with saturated aqueous NH_4_Cl (15 mL), and the resulting mixture was extracted with AcOEt (3 × 20 mL). The combined organic extracts were washed with brine (20 mL), and dried over anhydrous Na_2_SO_4_. Filtration and evaporation in vacuo furnished the pale yellow oil (869 mg), which was purified by column chromatography (silica gel 20 g, 10:1 → 6:1 → 3:1 *n*-hexane/AcOEt) to give TBS ether **45** (42.1 mg, 48%) as a pale yellow amorphous. *R_f_* 0.46 (3:1 *n*-hexane/AcOEt); [α${]}_{D}^{22}$ +23.2 (*c* 1.00, CHCl_3_); IR (neat) 3534, 2953, 2927, 2859, 1755, 1471, 1462, 1336, 1253, 1197, 1149, 1099, 994 cm^−1^; ^1^H-NMR (500 MHz, C_6_D_6_) δ 0.08 (s, 6H, Si(C*H*_3_)_2_), 0.65 (d, *J* = 6.6 Hz, 3H, C8-C*H*_3_), 0.78 (br s, 1H, O*H*), 0.96 (m, 1H, one of C1-*H*_2_), 0.97 (s, 6H, C4-C*H*_3_, C10-C*H*_3_), 1.00 (s, 9H, SiC(C*H*_3_)_3_), 1.14−1.30 (m, 6H, one of C2-*H*_2_, one of C3-*H*_2_, C11-*H*_2_, C12-*H*_2_), 1.35−1.45 (m, 4H, one of C2-*H*_2_, one of C7-*H*_2_, C13-*H*_2_), 1.47 (m, 1H, one of C1-*H*_2_), 1.77 (ddq, *J* = 6.2, 11.2, 6.6 Hz, 1H, C8-*H*), 1.92 (dd, *J* = 6.2, 15.8 Hz, 1H, one of C7-*H*_2_), 2.00 (d, *J* = 4.6 Hz, 1H, C5-*H*), 2.18 (dt, *J* = 4.3, 13.7 Hz, 1H, one of C3-*H*_2_), 3.52 (t, *J* = 6.1 Hz, 2H, C14-*H*_2_), 4.36 (dd, *J* = 4.6, 6.4 Hz, 1H, C6-*H*); ^13^C-NMR (125.7 MHz, C_6_D_6_) δ −5.1 (CH_3_), 16.6 (CH_3_), 18.51 (C), 18.55 (CH_2_), 21.9 (CH_2_), 22.6 (CH_3_), 22.9 (CH_3_), 26.1 (CH_3_), 28.8 (CH_2_), 28.9 (CH_2_), 32.0 (CH_2_), 32.3 (CH), 34.0 (CH_2_), 34.8 (CH_2_), 39.8 (C), 43.7 (C), 44.9 (CH), 62.8 (CH_2_), 75.48 (C), 75.52 (C), 182.6 (C); HRMS (ESI) *m*/*z* [M + Na]^+^ calcd for C_24_H_44_O_4_SiNa 447.2907; found 447.2891.

*(1S,4R,6R,7R,8S,12R)-7-Hydroxy-7-(4-hydroxybutyl)-1,6,8-trimethyl-2-oxo-3-oxatricyclo[6.3.1.0^4,12^]dodecane* (**46**). Bu_4_NF in THF (1.0 M, 0.38 mL, 0.38 mmol) was added to an ice-cooled (0 °C) solution of TBS ether **45** (32.1 mg, 0.76 mmol) in THF (1.0 mL). After 1 h of stirring at room temperature, the mixture was partitioned between AcOEt (2 mL) and H_2_O (2 mL), and the aqueous layer was extracted with AcOEt (3 × 2 mL). The combined organic extracts were washed with brine (5 mL), and dried over anhydrous Na_2_SO_4_. Filtration and evaporation in vacuo furnished the pale yellow oil (144 mg), which was purified by column chromatography (silica gel 2 g, 1:4 *n*-hexane/AcOEt) to give diol **46** (19.3 mg, 82%) as a colorless amorphous. *R_f_* 0.33 (1:2 *n*-hexane/AcOEt); [α${]}_{D}^{22}$ +24.4 (*c* 0.90, CHCl_3_); IR (neat) 3478, 2951, 2930, 2870, 1747, 1462, 1454, 1350, 1263, 1199, 1149, 1099, 1072, 1041, 991 cm^−1^; ^1^H-NMR (500 MHz, C_6_D_6_) δ 0.54 (s, 1H, O*H*), 0.61 (d, *J* = 6.7 Hz, 3H, C8-C*H*_3_), 0.86 (s, 1H, O*H*), 0.91 (m, 1H, one of C1-*H*_2_), 0.95 (s, 3H, C10-C*H*_3_), 0.97 (s, 3H, C4-C*H*_3_), 1.07−1.30 (m, 8H, one of C1-*H*_2_, one of C2-*H*_2_, C11-*H*_2_, C12-*H*_2_, C13-*H*_2_), 1.36−1.48 (m, 3H, one of C2-*H*_2_, one of C3-*H*_2_, one of C7-*H*_2_), 1.73 (ddq, *J* = 6.2, 11.3, 6.7 Hz, 1H, C8-*H*), 1.91 (ddd, *J* = 1.3, 6.2, 15.9 Hz, 1H, one of C7-*H*_2_), 2.00 (d, *J* = 4.8 Hz, 1H, C5-*H*), 2.18 (dt, *J* = 4.2, 13.8 Hz, 1H, one of C3-*H*_2_), 3.30 (t, *J* = 6.1 Hz, 2H, C14-*H*_2_), 4.35 (ddd, *J* = 1.3, 4.8, 6.2 Hz, 1H, C6-*H*); ^13^C-NMR (125.7 MHz, C_6_D_6_) δ 16.6 (CH_3_), 18.6 (CH_2_), 21.8 (CH_2_), 22.5 (CH_3_), 22.9 (CH_3_), 28.7 (CH_2_), 28.9 (CH_2_), 32.0 (CH_2_), 32.3 (CH), 33.7 (CH_2_), 34.7 (CH_2_), 39.8 (C), 43.7 (C), 44.9 (CH), 62.2 (CH_2_), 75.4 (C), 75.6 (CH), 182.7 (C); HRMS (ESI) *m*/*z* [M + Na]^+^ calcd for C_18_H_30_O_4_Na 333.2042; found 333.2052.

*Marrulactone* (**14**). TEMPO (2.3 mg, 14.7 μmol) was added to an ice-cooled (0 °C) mixture of diol **46** (13.1 mg, 42.2 μmol) and PhI(OAc)_2_ (40.5 mg, 0.125 mmol) in CH_2_Cl_2_ (0.3 mL). After 4 h of stirring at room temperature, the reaction was quenched with 1 M aqueous Na_2_S_2_O_3_ (1 mL), and the resulting mixture was extracted with AcOEt (3 × 1 mL). The combined organic extracts were washed with brine (3 mL), and dried over anhydrous Na_2_SO_4_. Filtration and evaporation in vacuo furnished the colorless oil (64.0 mg), which was purified by column chromatography (silica gel 1 g, 1:1 *n*-hexane/AcOEt) to give marrulactone (**14**, 11.8 mg, 91%) as a colorless amorphous. *R_f_* 0.60 (1:2 *n*-hexane/AcOEt); [α${]}_{D}^{22}$ −11.6 (*c* 0.61, CHCl_3_) [lit. [12], [α${]}_{D}^{25}$ −23.80 (*c* 0.22, CHCl_3_)]; IR (neat) 3017, 2961, 2928, 2872, 1769, 1719, 1458, 1391, 1329, 1261, 1244, 1215, 1188, 1117, 1070, 1030, 1003 cm^−^^1^; ^1^H-NMR (500 MHz, CDCl_3_) δ 0.98 (d, *J* = 6.3 Hz, 3H, C8-C*H_3_*), 1.11 (s, 3H, C10-C*H_3_*), 1.31 (s, 3H, C4-C*H_3_*), 1.34−1.42 (m, 2H, C1-*H_2_*), 1.48 (m, 1H, one of C3-*H_2_*), 1.55 (m, 1H, one of C2-*H_2_*), 1.74−1.80 (m, 2H, one of C2-*H_2_*, one of C11-*H_2_*), 1.80−1.94 (m, 4H, one of C7-*H_2_*, one of C11-*H_2_*, C12-*H_2_*), 2.09−2.20 (m, 3H, one of C3-*H_2_*, one of C7-*H_2_*, C8-*H*), 2.25 (ddd, *J* = 6.2, 11.2, 17.3 Hz, 1H, one of C13-*H_2_*), 2.32 (d, *J* = 4.4 Hz, 1H, C5-*H*), 2.57 (ddt, *J* = 2.0, 17.3, 4.1 Hz, 1H, one of C13-*H_2_*), 4.78 (ddd, *J* = 0.7, 4.4, 5.5 Hz, 1H, C6-*H*); ^13^C-NMR (125.7 MHz, CDCl_3_) δ 16.5 (CH_3_), 17.8 (CH_2_), 18.9 (CH_2_), 22.0 (CH_3_), 22.8 (CH_3_), 26.1 (CH_2_), 28.1 (CH_2_), 28.6 (CH_2_), 30.4 (CH_2_), 30.9 (CH_2_), 34.3 (CH), 40.8 (C), 44.0 (C), 44.3 (CH), 75.8 (CH), 88.3 (C), 172.2 (C), 183.5 (C); HRMS (ESI) *m*/*z* [M + Na]^+^ calcd for C_18_H_26_O_4_Na 329.1723; found 329.1711.

*Marrulanic acid* (**10**). LiOH (18.7 mg, 0.78 mmol) was added to a solution of marrulactone (**14**, 11.8 mg, 38.5 μmol) in THF/H_2_O (2:1, 1.2 mL). After 31 h of stirring, the mixture was acidified with 1 M aqueous HCl (3 mL), and the resulting mixture was extracted with AcOEt (3 × 3 mL). The combined organic extracts were washed with brine (5 mL), and dried over anhydrous Na_2_SO_4_. Filtration and evaporation in vacuo furnished the colorless oil (11.7 mg), which was purified by column chromatography (silica gel 1 g, 1:1 *n*-hexane/AcOEt → AcOEt → 20:1 AcOEt/MeOH) to give marrulanic acid (**10**, 10.5 mg, 84%) as a colorless amorphous. *R_f_* 0.19 (1:2 *n*-hexane/AcOEt); [α${]}_{D}^{27}$ +25.4 (*c* 0.53, CHCl_3_) [lit. [12], [α${]}_{D}^{25}$ −10.8 (*c* 1.2, CHCl_3_)]; IR (neat) 3536, 3196, 2949, 2928, 2868, 1761, 1732, 1464, 1412, 1391, 1287, 1260, 1196, 1179, 1159, 1126, 1094, 1078, 1042, 1018 cm^−1^; ^1^H-NMR (500 MHz, CDCl_3_) δ 0.92 (d, *J* = 6.5 Hz, 3H, C8-C*H*_3_), 1.04 (s, 3H, C10-C*H*_3_), 1.26 (m, 1H, one of C1-*H_2_*), 1.29 (s, 3H, C4-C*H*_3_), 1.44−1.80 (m, 9H, one of C1-*H_2_*, C2-*H*_2_, one of C3-*H_2_*, one of C7-*H_2_*, C11-*H*_2_, C12-*H*_2_), 2.04−2.17 (m, 3H, one of C3-*H_2_*, one of C7-*H_2_*, C8-*H*), 2.24 (d, *J* = 4.6 Hz, 1H, C5-*H*), 2.38 (dt, *J* = 1.8, 7.0 Hz, 2H, C13-*H*_2_), 4.74 (ddd, *J* = 1.2, 4.6, 5.8 Hz, 1H, C6-*H*); ^13^C-NMR (125.0 MHz, CDCl_3_) δ 16.5 (CH_3_), 18.2 (CH_2_), 20.3 (CH_2_), 22.3 (CH_3_), 22.9 (CH_3_), 28.3 (CH_2_), 28.6 (CH_2_), 31.5 (CH_2_), 32.2 (CH), 34.29 (CH_2_), 34.31 (CH_2_), 39.7 (C), 43.8 (C), 44.8 (CH), 75.6 (C), 76.2 (CH), 178.5 (C), 183.9 (C); HRMS (ESI) *m*/*z* [M + Na]^+^ calcd for C_18_H_28_O_5_Na 347.1834; found 347.1813.

*[1S,4R,6R,7R,7(2S,3S,4R,5R),8S,12R]-7-[2-(3,4-Dihydroxy-2,5-dimethoxytetrahydrofuran-3-yl)ethyl]-7-hydroxy-1,6,8-trimethyl-2-oxo-3-oxatricyclo[6.3.1.0^4,12^]dodecane* (**40**). TsOH (0.4 mg, 2.3 μmol) was added to a solution of marrulibacetal A (**17**, 0.90 mg, 2.3 μmol) in MeOH (1.0 mL), and the mixture was heated at reflux for 5 h. After cooling to 0 °C, the reaction was quenched with half saturated aqueous NaHCO_3_ (4 mL), and the resulting mixture was extracted with AcOEt (2 × 5 mL). The combined organic extracts were washed with brine (5 mL), and dried over anhydrous Na_2_SO_4_. Filtration and evaporation in vacuo furnished the colorless oil (1.4 mg), which was purified by flash column chromatography (silica gel 0.8 g, 1:1 *n*-hexane/AcOEt → AcOEt) to give triol **40** (0.24 mg, 25%) as a colorless oil. *R_f_* 0.30 (1:1 *n*-hexane/AcOEt); [α${]}_{D}^{23}$ +16.5 (*c* 0.46, benzene); IR (neat) 3447, 2930, 1744, 1466, 1449, 1389, 1288, 1198, 1130, 1094, 1042, 991 cm^−1^; ^1^H-NMR (500 MHz, CDCl_3_) δ 0.91 (d, *J* = 6.4 Hz, 3H, C8-C*H*_3_), 1.05 (s, 3H, C10-C*H*_3_), 1.28 (m, 1H, one of C1-*H*_2_), 1.29 (s, 3H, C4-C*H*_3_), 1.45 (m, 1H, one of C3-*H*_2_), 1.51 (m, 1H, one of C2-H_2_), 1.65 (m, 1H, one of C7-*H*_2_), 1.74 (m, 1H, one of C2-*H*_2_), 1.60−1.98 (m, 5H, one of C1-*H*_2_, C11-*H*_2_, C12-*H*_2_), 2.07 (m, 1H, C8-*H*), 2.11 (m, 1H, one of C3-*H*_2_), 2.14 (m, 1H, one of C7-*H*_2_), 2.22 (d, *J* = 4.5 Hz, 1H, C5-*H*), 3.40 (s, 3H, C16-OC*H*_3_), 3.48 (s, 3H, C15-OC*H*_3_), 3.92 (d, *J* = 3.7 Hz, 1H, C14-*H*), 4.73 (dd, *J* = 4.5, 6.5 Hz, 1H, C6-H), 4.77 (s, 1H, C16-*H*), 4.89 (d, *J* = 3.7 Hz, 1H, C15-*H*); ^13^C-NMR (125.7 MHz, CDCl_3_) δ 16.4 (CH_3_), 18.1 (CH_2_), 22.3 (CH_3_), 22.9 (CH_3_), 28.0 (CH_2_), 28.26 (CH_2_), 28.28 (CH_2_), 28.4 (CH_2_), 31.5 (CH_2_), 32.6 (CH), 40.0 (C), 43.9 (C), 44.9 (CH), 55.1 (CH_3_), 56.4 (CH_3_), 75.4 (C), 76.2 (CH), 80.3 (CH), 81.4 (C), 108.3 (CH), 110.7 (CH), 184.0 (C); HRMS (ESI) m/z [M + Na]^+^ calcd for C_22_H_36_O_8_Na 451.2302; found 451.2308.

*Desertine* (**18**). TsOH (1.6 mg, 9.3 μmol) was added to a solution of bisacetal **42** (2.0 mg, 4.7 μmol) in MeOH (0.5 mL), and the mixture was heated at reflux for 3.5 h. After cooling to 0 °C, the reaction was quenched with saturated aqueous NaHCO_3_ (2 mL), and the resulting mixture was extracted with AcOEt (2 × 5 mL). The combined organic extracts were washed with brine (10 mL), and dried over anhydrous Na_2_SO_4_. Filtration and evaporation in vacuo furnished the pale yellow oil (3.6 mg), which was purified by flash column chromatography (silica gel 0.8 g, 1:1 *n*-hexane/AcOEt) to give desertine (**18**, 0.34 mg, 16%) as a colorless oil. *R_f_* 0.30 (1:1 *n*-hexane/AcOEt); [α${]}_{D}^{23}$ +22.8 (*c* 0.50, benzene) (lit. [14], [α${]}_{D}^{25}$ −56.6); IR (neat) 3464, 2930, 1748, 1463, 1450, 1391, 1256, 1198, 1152, 1099, 1043, 989 cm^−1^; ^1^H-NMR (500 MHz, CDCl_3_) δ 0.91 (d, *J* = 6.2 Hz, 3H, C8-C*H*_3_), 1.04 (s, 3H, C10-C*H*_3_), 1.28 (m, 1H, one of C1-*H*_2_), 1.29 (s, 3H, C4-C*H*_3_), 1.45 (m, 1H, one of C3-*H*_2_), 1.51 (m, 1H, one of C2-*H*_2_), 1.65 (m, 1H, one of C7-*H*_2_), 1.74 (m, 1H, one of C2-*H*_2_), 1.60−1.98 (m, 5H, one of C1-*H*_2_, C11-*H*_2_, C12-*H*_2_), 2.07 (m, 1H, C8-*H*), 2.11 (m, 1H, one of C3-*H*_2_), 2.14 (m, 1H, one of C7-*H*_2_), 2.23 (d, *J* = 4.0 Hz, 1H, C5-*H*), 3.41 (s, 3H, C16-OC*H*_3_), 3.47 (s, 3H, C15-OC*H*_3_), 3.95 (d, *J* = 3.5 Hz, 1H, C14-*H*), 4.73 (dd, *J* = 4.0, 6.9 Hz, 1H, C6-*H*), 4.76 (s, 1H, C16-*H*), 4.90 (d, *J* = 3.5 Hz, 1H, C15-*H*); ^13^C-NMR (125.7 MHz, CDCl_3_) δ 16.8 (CH_3_), 18.2 (CH_2_), 22.2 (CH_3_), 23.0 (CH_3_), 27.4 (CH_2_), 28.3 (CH_2_), 28.4 (CH_2_), 28.5 (CH_2_), 31.6 (CH_2_), 32.5 (CH), 40.1 (C), 43.9 (C), 45.0 (CH), 55.1 (CH_3_), 56.4 (CH_3_), 75.3 (C), 76.2 (CH), 80.2 (CH), 81.2 (C), 108.6 (CH), 110.8 (CH), 183.8 (C); HRMS (ESI) *m*/*z* [M + Na]^+^ calcd for C_22_H_36_O_8_Na 451.2302; found 451.2313.

## 4. Conclusions

Total syntheses of eight labdane diterpene lactones, marrubiin (**1**), marrulanic acid (**10**), cyllenine C (**12**), marrulibacetal (**13**), marrulactone (**14**), marrulibacetal A (**17**), desertine (**18**), and marrubasch F (**19**), have been accomplished. Since chiral building block **20** could be prepared from (4*R*,6*S*)-6-[(*tert*-butyldimethylsilyl)oxymethyl]-4-methyltetrahydro-2*H*-pyran-2-one [53] in 37% yield over eight steps, the syntheses proceeded in 19−22 steps involving a stereoselective intramolecular PKR, and these natural products were obtained in yields ranging from 1.1% (marrulibacetal A) to 10.9% (cyllenin C). Stereochemistries of desertine were established by the facts that the compound could be obtained by both osmylation of bisacetal **37** and transacetalization of the diastereomer of marrulibacetal A with MeOH. The syntheses illustrate the synthetic utility of chiral building block **20** for the synthesis of this class of diterpenoids. The synthetic strategy also features elongation of the C9 side chain through an epoxide-opening reaction as well as high convergency and flexibility. The syntheses of other natural products and non-natural analogues for biological and pharmaceutical investigations are in progress in our laboratory and will be reported in due course.

## Supplementary Materials

The following are available online. Comparison data for natural products and synthetic materials, and copies of ^1^H and ^13^C-NMR spectra for all new compounds.
