# Supplementary material for: Total Syntheses of Marrubiin and Related Labdane Diterpene Lactones
Source: Molecules. 2020 Apr 1;25(7):1610. doi: 10.3390/molecules25071610 (PMC7180712; doi:10.3390/molecules25071610)
Supplement: Supplementary file 1 [file molecules-25-01610-s001.pdf]

Supplementary Materials

Total Syntheses of Marrubiin and Related Labdane Diterpene Lactones

Yukari Sakagami, Naoki Kondo, Yuki Sawayama, Hiroyuki Yamakoshi, and Seiichi Nakamura*

Graduate School of Pharmaceutical Sciences, Nagoya City University, 3-1 Tanabe-dori, Mizuho-ku, Nagoya 467-8603, Japan

nakamura@phar.nagoya-cu.ac.jp

Table of Contents

1. Comparison of Spectral Data for Synthetic and Natural Compounds 2

2. Copies of ^{1}H and ^{13}C NMR Spectra 11

1. Comparison of Spectral Data for Synthetic and Natural Compounds

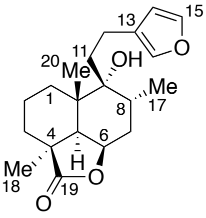

marrubiin (**1**)

position	natural			synthetic		
	^{1}H		^{13}C	^{1}H		^{13}C
1a	1.30	dt, *J* = 14.4, 3.6 Hz	28.6	1.32	dt, *J* = 12.8, 8.5 Hz	28.6
1b	1.66			1.66−1.79	m	
2a	1.49	t, *J* = 3.6, 13.6 Hz	18.2	1.43−1.55	m	18.2
2b	1.71			1.66−1.79	m	
3a	1.44	ddd, *J* = 3.6, 13.5,	28.4	1.43−1.55	m	28.3
3b	2.10			2.09−2.18	m	
4			43.8			43.8
5	2.22	d, *J* = 4.7 Hz	44.9	2.23	d, *J* = 4.6 Hz	44.8
6	4.73	ddd, *J* = 1.6, 4.7, 6.4	76.2	4.74	br dd, *J* = 4.6, 6.5 Hz	76.2
7a	1.67		31.5	1.66−1.79	m	31.5
7b	2.15	ddd, *J* = 1.6, 6.4,		2.09−2.18	m	
8	2.10		32.3	2.09−2.18	m	32.4
9			75.8			75.8
10			39.7			39.7
11a	1.75	ddd, *J* = 7.2, 10.4, 14.6 Hz	35.1	1.66−1.79	m	35.1
11b	1.89	ddd, *J* = 7.2, 10.4, 14.6 Hz		1.90	ddd, *J* = 7.2, 10.1, 14.4 Hz	
12a	2.52		21.0	2.48−2.58	m	21.0
12b	2.52			2.48−2.58	m	
13			125.0			125.0
14	6.26	dd, *J* = 0.8, 1.7 Hz	110.7	6.27	s	110.7
15	7.35	t, *J* = 1.7 Hz	143.1	7.37	s	143.1
16	7.22	ddt, *J* = 0.8, 1.7, 1.0 Hz	138.6	7.24	s	138.6
17	0.96	d, *J* = 6.4 Hz	16.6	0.97	d, *J* = 6.4 Hz	16.6
18	1.28	s	23.0	1.29	s	22.9
19			183.8			183.8
20	1.05	d, *J* = 0.8 Hz	22.2	1.07	s	22.3

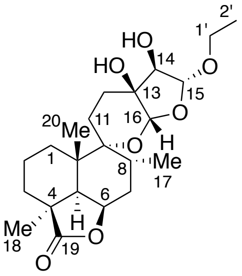

marrulibacetal (**13**)

position	natural			synthetic		
	^{1}H		^{13}C	^{1}H		^{13}C
1a	1.18		27.9	1.19	m	27.8
1b	1.97			1.98	m	
2a	1.50		17.9	1.49	m	17.9
2b	1.73			1.71−1.78	m	
3a	1.42	m	28.2	1.42	m	28.2
3b	2.10			2.05−2.22	m	
4			43.9			43.9
5	2.35	d, *J* = 4.5 Hz	44.7	2.36	d, *J* = 4.7 Hz	44.6
6	4.77	br dd, *J* = 4.5, 5.3 Hz	76.5	4.77	br dd, *J* = 4.7, 5.9 Hz	76.5
7a	1.86		32.3	1.80−1.90	m	32.3
7b	2.17			2.05−2.22	m	
8	2.08		33.7	2.05−2.22	m	33.6
9			80.4			80.4
10			41.0			40.9
11a	1.75		21.1	1.71−1.78	m	21.1
11b	1.80			1.80−1.90	m	
12a	1.85		29.6	1.80−1.90	m	29.6
12b	2.08			2.05−2.22	m	
13			75.6			75.6
14	3.94	br s	78.6	3.95	dd, *J* = 2.0, 6.2 Hz	78.5
15	5.03	d, *J* = 1.7 Hz	108.7	5.04	d, *J* = 2.0 Hz	108.7
16	5.45	s	105.4	5.46	s	105.3
17	1.10	d, *J* = 6.5 Hz	19.5	1.11	d, *J* = 6.8 Hz	19.5
18	1.26	s	23.2	1.28	s	23.1
19			183.9			184.0
20	1.02	s	22.0	1.03	s	22.1
1’a	3.50	dq, *J* = 14.2, 7.0 Hz	64.0	3.51	dq, *J* = 9.5, 7.1 Hz	63.9
1’b	3.81	dq, *J* = 14.2, 7.0 Hz		3.82	dq, *J* = 9.5, 7.1 Hz	
2’	1.20	t, *J* = 7.0 Hz	15.1	1.22	t, *J* = 7.1 Hz	15.0

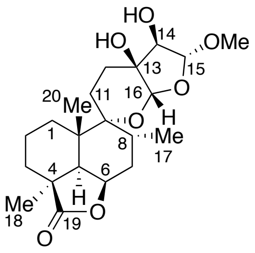

marrulibacetal A (**17**)

position	natural			synthetic		
	^{1}H		^{13}C	^{1}H		^{13}C
1a			27.8	1.19	m	27.8
1b	1.96	m		1.96	t, *J* = 10.6 Hz	
2a	1.44	m	17.9	1.49	m	17.9
2b	1.72	m		1.71−1.92	m	
3a	2.06	m	28.2	1.42	m	28.2
3b	2.12	m		2.05−2.15	m	
4			43.9			43.9
5	2.38	d, *J* = 5.0 Hz	44.6	2.38	d, *J* = 4.5 Hz	44.6
6	4.78	t, *J* = 5.0 Hz	76.6	4.79	dd, *J* = 4.5, 6.3 Hz	76.5
7a	2.18	m	32.3	1.71−1.92	m	32.2
7b	2.22	m		2.20	dd, *J* = 5.5, 16.0 Hz	
8	2.07	m	33.5	2.05−2.15	m	33.5
9			80.3			80.3
10			40.9			40.9
11a	1.77	m	20.7	1.71−1.92	m	20.7
11b	1.77	m		1.71−1.92	m	
12a	1.86	m	30.0	1.71−1.92	m	30.0
12b	2.08	m		2.05−2.15	m	
13			75.8			75.8
14	3.88	s	78.6	3.89	d, *J* = 1.2 Hz	78.6
15	4.93	d, *J* = 1.5 Hz	109.8	4.93	d, *J* = 1.2 Hz	109.8
16	5.47	s	105.7	5.48	s	105.7
17	1.10	d, *J* = 6.8 Hz	19.4	1.11	d, *J* = 7.0 Hz	19.4
18	1.28	s	23.2	1.28	s	23.1
19			184.1			184.1
20	1.03	s	22.0	1.03	s	22.0
15-OMe	3.41	s	55.5	3.42	s	55.5

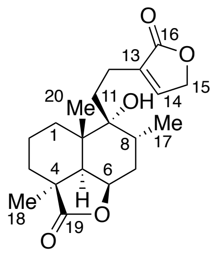

marrubasch F (**19**)

position	natural ^{1}H		natural ^{13}C	synthetic ^{1}H		synthetic ^{13}C
1a				1.25	dt, *J* = 12.8, 8.5 Hz	28.6
1b				1.69−1.84	m	
2a				1.53	m	18.1
2b				1.69−1.84	m	
3a				1.45	dt, *J* = 14.4, 4.8 Hz	28.3
3b				2.05−2.17	m	
4						43.8
5				2.28	d, *J* = 4.5 Hz	44.9
6	4.70−4.90			4.75	dd, *J* = 4.5, 6.5 Hz	76.2
7a				1.69−1.84	m	31.6
7b				2.05−2.17	m	
8				2.05−2.17	m	32.6
9						75.2
10						39.9
11a				1.69−1.84	m	32.4
11b				1.69−1.84	m	
12a				2.41−2.52	m	21.2
12b				2.41−2.52	m	
13						134.7
14	7.20	br s		7.14	t, *J* = 1.4 Hz	144.3
15a	4.70−4.90			4.80	br d, *J* = 1.4 Hz	70.4
15b	4.70−4.90			4.80	br d, *J* = 1.4 Hz	
16						174.9
17	0.99	d, *J* = 6 Hz		0.96	d, *J* = 6.4 Hz	16.7
18	1.30	s		1.30	s	22.9
19						183.9
20	1.04	s		1.04	s	22.3

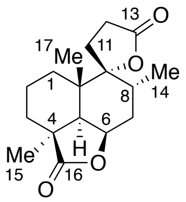

cyllenine C (**12**)

position	natural			synthetic		
	^{1}H		^{13}C	^{1}H		^{13}C
1a	1.27−1.31		27.8	1.30	dt, *J* = 12.2, 9.8 Hz	27.7
1b	1.41−1.51	m		1.46	m	
2a	1.50	ddd, *J* = 4.8, 13.3, 13.8 Hz	17.7	1.52	m	17.7
2b	1.76	m		1.78	m	
3a	1.47	m	28.3	1.49	m	28.2
3b	2.09	ddd, *J* = 4.9, 13.0, 14.9 Hz		2.12	m	
4			44.0			44.0
5	2.16	d, *J* = 4.6 Hz	45.3	2.19	d, *J* = 4.5 Hz	45.3
6	4.72	dd, *J* = 5.0, 5.4 Hz	75.6	4.75	dd, *J* = 4.5, 6.3 Hz	75.6
7a	1.69	dddd, *J* = 5.6, 6.4, 12.1, 16.4 Hz	31.2	1.72	ddd, *J* = 6.3, 11.9, 16.4 Hz	31.2
7b	2.26	dd, *J* = 6.6, 16.4 Hz		2.28	dd, *J* = 6.3, 16.4 Hz	
8	2.15		32.3	2.18	ddq, *J* = 6.3, 11.9,	32.3
9			91.2			91.2
10			38.6			38.6
11a	1.93	dt, *J* = 4.6, 11.2 Hz	24.5	1.95	ddd, *J* = 4.4, 11.4, 13.8 Hz	24.5
11b	2.20			2.22	ddd, *J* = 8.9, 11.5, 13.8 Hz	
12a	2.49−2.57		29.3	2.54	ddd, *J* = 4.4, 11.5, 18.8 Hz	29.3
12b	2.55−2.63			2.62	ddd, *J* = 8.9, 11.4, 18.8 Hz	
13			177.0			176.9
14	0.90	d, *J* = 6.7 Hz	15.4	0.93	d, *J* = 6.5 Hz	15.4
15	1.28	s	22.4	1.31	s	22.4
16			183.3			183.3
17	1.06	s	22.1	1.09	s	22.2

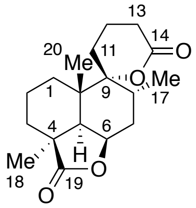

marrulactone (**14**)

position	natural			synthetic		
	^{1}H		^{13}C	^{1}H		^{13}C
1a			28.8	1.34−1.42	m	28.6
1b				1.34−1.42	m	
2a			18.4	1.55	m	17.8
2b				1.74−1.80	m	
3a			28.5	1.48	m	28.1
3b				2.09−2.20	m	
4			44.1			44.0
5	2.32	d, *J* = 4.4 Hz	45.0	2.32	d, *J* = 4.4 Hz	44.3
6	4.78	dd, *J* = 4.4, 5.4 Hz	75.7	4.78	ddd, *J* = 0.7, 4.4, 5.5 Hz	75.8
7a			31.8	1.80−1.94	m	30.9
7b				2.09−2.20	m	
8			32.5	2.09−2.20	m	34.3
9			88.3			88.3
10			39.9			40.8
11a			34.5	1.74−1.80	m	26.1
11b				1.80−1.94	m	
12a			20.5	1.80−1.94	m	18.9
12b				1.80−1.94	m	
13a			34.4	2.25	ddd, *J* = 6.2, 11.2, 17.3 Hz	30.4
13b	2.58	dt, *J* = 17.4, 3.9 Hz		2.57	ddt, *J* = 2.0, 17.3, 4.1 Hz	
14			172.1			172.2
17	0.98	d, *J* = 6.3 Hz	16.8	0.98	d, *J* = 6.3 Hz	16.5
18	1.31	s	23.1	1.31	s	22.8
19			183.4			183.5
20	1.11	s	22.6	1.11	s	22.0

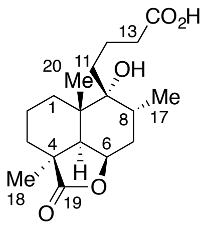

marrulanic acid (**10**)

position	natural ^1H		^{13}C	synthetic ^1H		^{13}C
1a	1.28	m	28.5	1.26	m	28.6
1b	1.74	m		1.44−1.80	m	
2a	1.48	m	18.1	1.44−1.80	m	18.2
2b	1.71	m		1.44−1.80	m	
3a	1.44	m	28.3	1.44−1.80	m	28.3
3b	2.11	m		2.04−2.17	m	
4			43.8			43.8
5	2.24	d, J = 4.4 Hz	44.7	2.24	d, J = 4.6 Hz	44.8
6	4.74	dd, J = 4.4, 5.6 Hz	76.3	4.74	ddd, J = 1.2, 4.6, 5.8 Hz	76.2
7a	1.65	m	31.5	1.44−1.80	m	31.5
7b	2.14	m		2.04−2.17	m	
8	2.05	m	32.2	2.04−2.17	m	32.2
9			75.5			75.6
10			39.7			39.7
11a	1.33		34.2	1.44−1.80	m	34.3
11b	1.48			1.44−1.80	m	
12a	1.72	m	20.2	1.44−1.80	m	20.3
12b	1.77	m		1.44−1.80	m	
13a	2.39	t, J = 6.4 Hz	34.1	2.38	dt, J = 1.8, 7.0 Hz	34.3
13b	2.39	t, J = 6.4 Hz		2.38	dt, J = 1.8, 7.0 Hz	
14			177.4			178.5
17	0.92	d, J = 6.0 Hz	16.5	0.92	d, J = 6.5 Hz	16.5
18	1.29	s	22.9	1.29	s	22.9
19			184.0			183.9
20	1.04	s	22.3	1.04	s	22.3

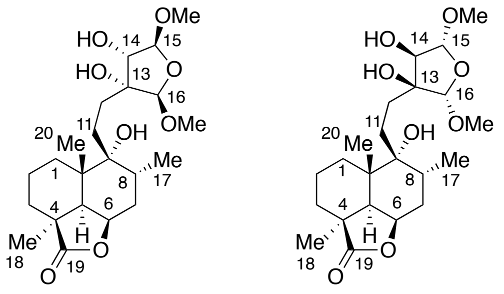

desertine (**18**)

isomer **40**

position	natural **18** ^{1}H		synthetic **18** ^{1}H		isomer **40** ^{1}H	
1a	1.65	m	1.28	m	1.28	m
1b	1.84	m	1.60−1.98	m	1.60−1.98	m
2a	1.51	m	1.51	m	1.51	m
2b	1.74	m	1.74	m	1.74	m
3a	1.45	m	1.45	m	1.45	m
3b	2.12	m	2.11	m	2.11	m
5	2.23	d, *J* = 7.5 Hz	2.23	d, *J* = 4.0 Hz	2.22	d, *J* = 4.5 Hz
6	4.73	m	4.73	dd, *J* = 4.0, 6.9 Hz	4.73	dd, *J* = 4.5, 6.5 Hz
7a	1.66	m	1.65	m	1.65	m
7b	2.14	m	2.14	m	2.14	m
8	2.06		2.07	m	2.07	m
11a	1.29	m	1.60−1.98	m	1.60−1.98	m
11b	1.29	m	1.60−1.98	m	1.60−1.98	m
12a	1.75	m	1.60−1.98	m	1.60−1.98	m
12b	1.75	m	1.60−1.98	m	1.60−1.98	m
14	3.95	br s	3.95	d, *J* = 3.5 Hz	3.92	d, *J* = 3.7 Hz
15	4.89	d, *J* = 3.5 Hz	4.90	d, *J* = 3.5 Hz	4.89	d, *J* = 3.7 Hz
16	4.75	s	4.76	s	4.77	s
17	0.91	d	0.91	d, *J* = 6.2 Hz	0.91	d, *J* = 6.4 Hz
18	1.29	s	1.29	s	1.29	s
20	1.04	s	1.04	s	1.05	s
15-OMe	3.47	s	3.47	s	3.48	s
16-OMe	3.40	s	3.41	s	3.41	s

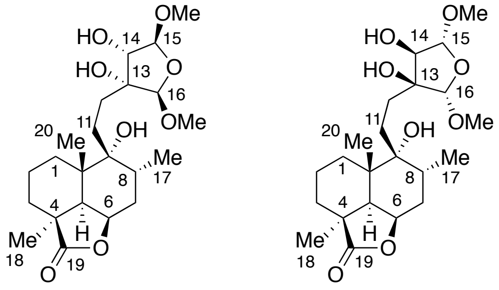

desertine (**18**) isomer **40**

	natural **18**	synthetic **18**	isomer **40**
position	^{13}C	^{13}C	^{13}C
1	28.4	28.4	28.3
2	18.2	18.2	18.2
3	28.3	28.3	28.2
4	43.9	43.9	43.9
5	45.0	45.0	44.9
6	76.3	76.2	76.0
7	31.6	31.6	31.5
8	32.5	32.5	32.7
9	75.3	75.3	75.4
10	40.1	40.1	40.0
11	28.5	28.5	28.5
12	27.4	27.4	28.0
13	81.2	81.2	81.4
14	80.2	80.2	80.4
15	110.8	110.8	110.7
16	108.6	108.6	108.3
17	16.8	16.8	16.5
18	23.0	23.0	22.9
19	183.9	183.8	183.8
20	22.2	22.2	22.3
15-OMe	56.4	56.4	56.4
16-OMe	55.1	55.1	55.1

2. Copies of ^{1}H and ^{13}C NMR Spectra

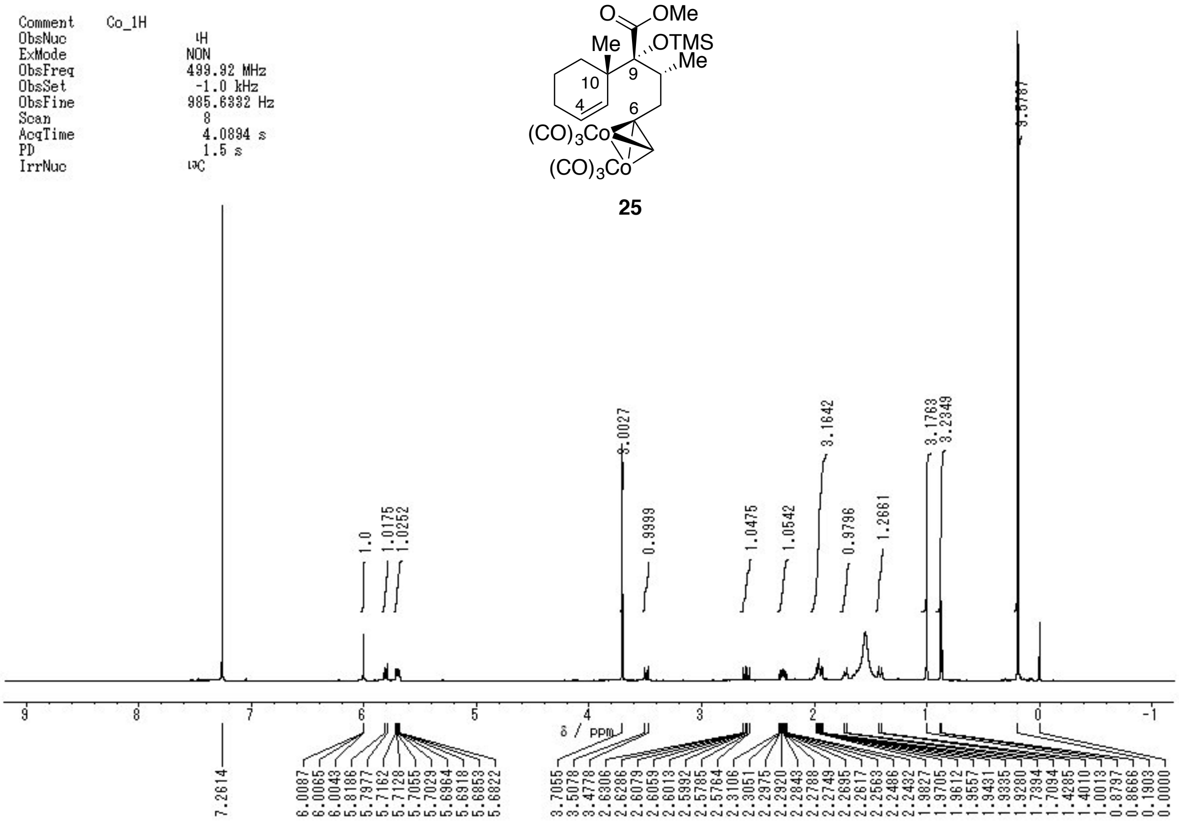

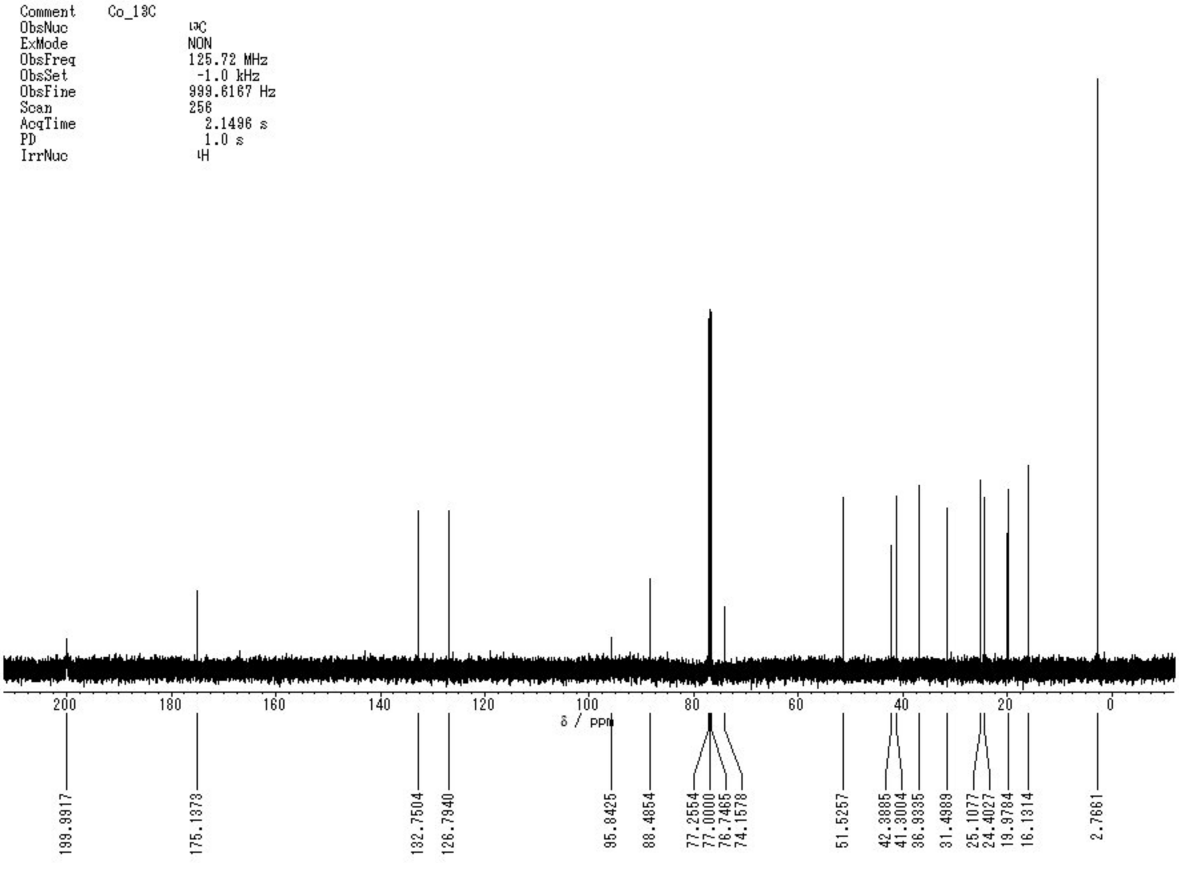

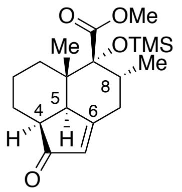
O
OMe
Me
OTMS
Me
8
5
4
6
H
H
O

trans-**24**

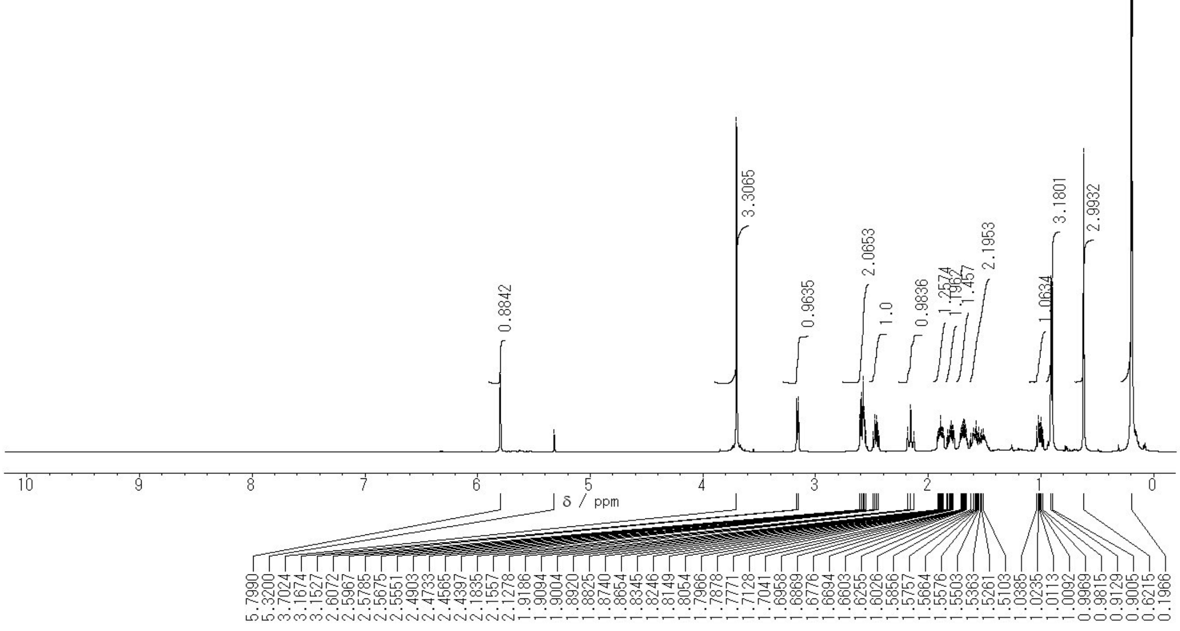
Comment 01PKR
ObsNuc 1H
ExMode NON
ObsFreq 499.92 MHz
ObsSet -1.0 kHz
ObsFine 997.0383 Hz
Scan 16
AcqTime 4.0894 s
PD 1.5 s
IrrNuc 13C
0.8842
3.3065
0.9635
2.0653
1.0
0.9836
1.2574
1.457
2.1953
1.0634
3.1801
2.9932
9.8528
10 9 8 7 6 5 4 3 2 1 0
δ / ppm
5.7990
5.3200
3.7024
3.1674
3.1527
2.6072
2.5967
2.5785
2.5675
2.5551
2.4903
2.4733
2.4565
2.4397
2.1835
2.1557
2.1278
1.9186
1.9094
1.9004
1.8920
1.8825
1.8740
1.8654
1.8345
1.8246
1.8149
1.8054
1.7966
1.7878
1.7771
1.7128
1.7041
1.6958
1.6869
1.6776
1.6694
1.6603
1.6255
1.6026
1.5856
1.5757
1.5664
1.5576
1.5503
1.5363
1.5261
1.5103
1.0385
1.0235
1.0113
1.0092
0.9969
0.9815
0.9129
0.9005
0.6215
0.1966

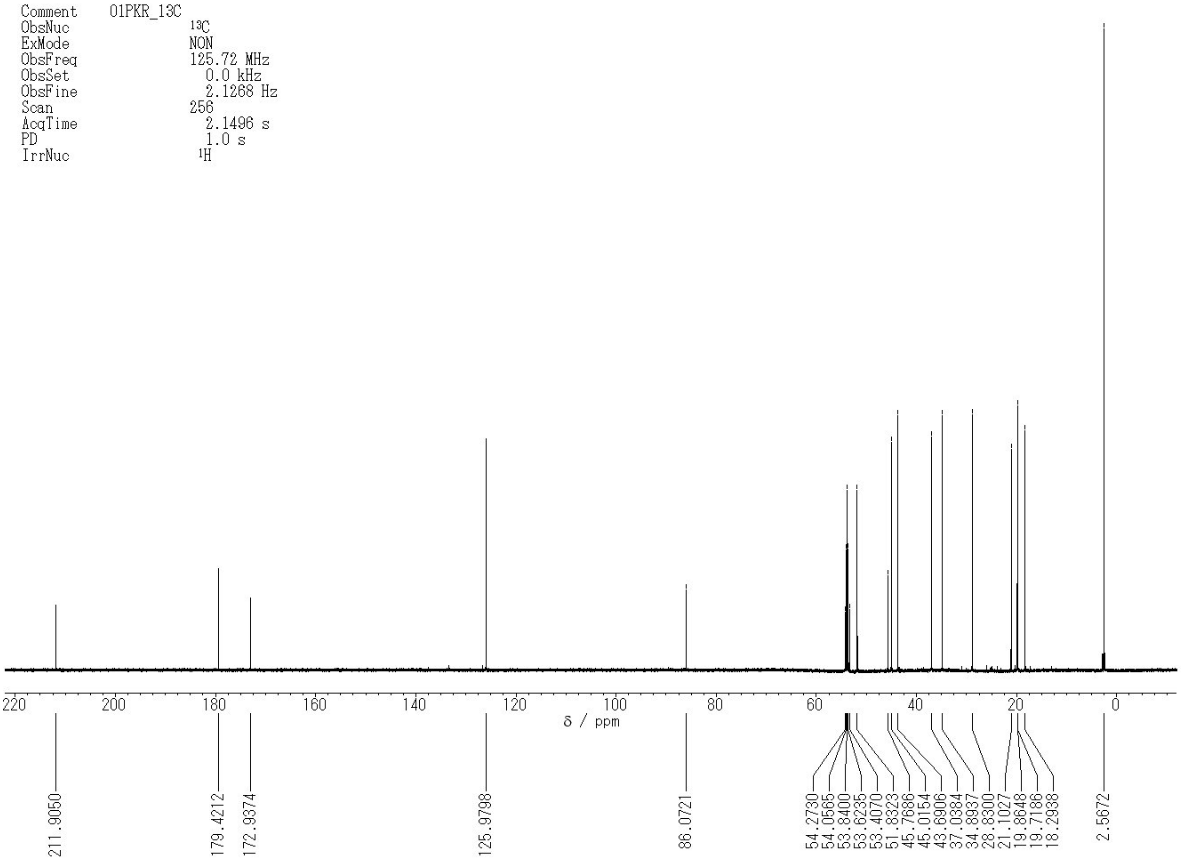
Comment 01PKR_13C
ObsNuc 13C
ExMode NON
ObsFreq 125.72 MHz
ObsSet 0.0 kHz
ObsFine 2.1268 Hz
Scan 256
AcqTime 2.1496 s
PD 1.0 s
IrrNuc 1H
220 200 180 160 140 120 100 80 60 40 20 0
δ / ppm
211.9050
179.4212
172.9374
125.9798
86.0721
54.2730
54.0565
53.8400
53.6235
53.4070
51.8323
45.7686
45.0154
43.6906
37.0384
34.8937
28.8300
21.1027
19.8648
19.7186
18.2938
2.5672

Comment 02Me_1H
ObsNuc ^{1}H
ExMode NON
ObsFreq 499.92 MHz
ObsSet -1.0 kHz
ObsFine 985.6332 Hz
Scan 16
AcqTime 3.5 s
PD 1.5 s
IrrNuc ^{13}C

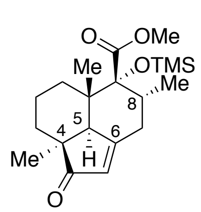

26

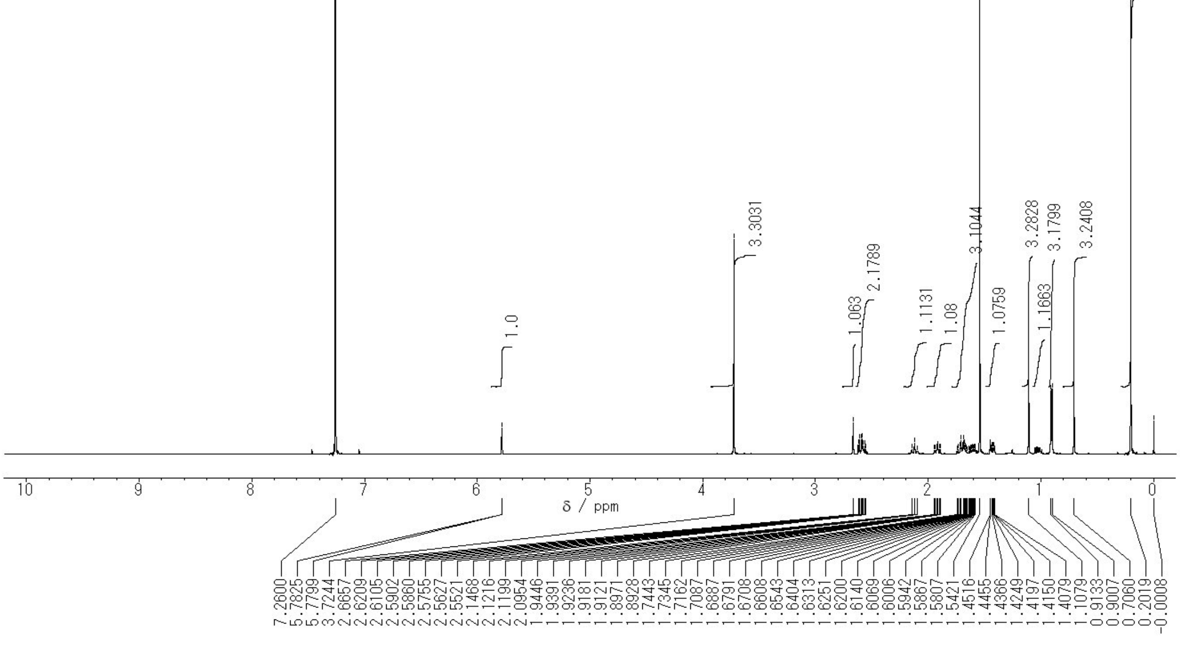

Comment 02Me_13C
ObsNuc ^{13}C
ExMode NON
ObsFreq 125.72 MHz
ObsSet 0.0 kHz
ObsFine 3.5755 Hz
Scan 256
AcqTime 2.1496 s
PD 1.0 s
IrrNuc ^{1}H

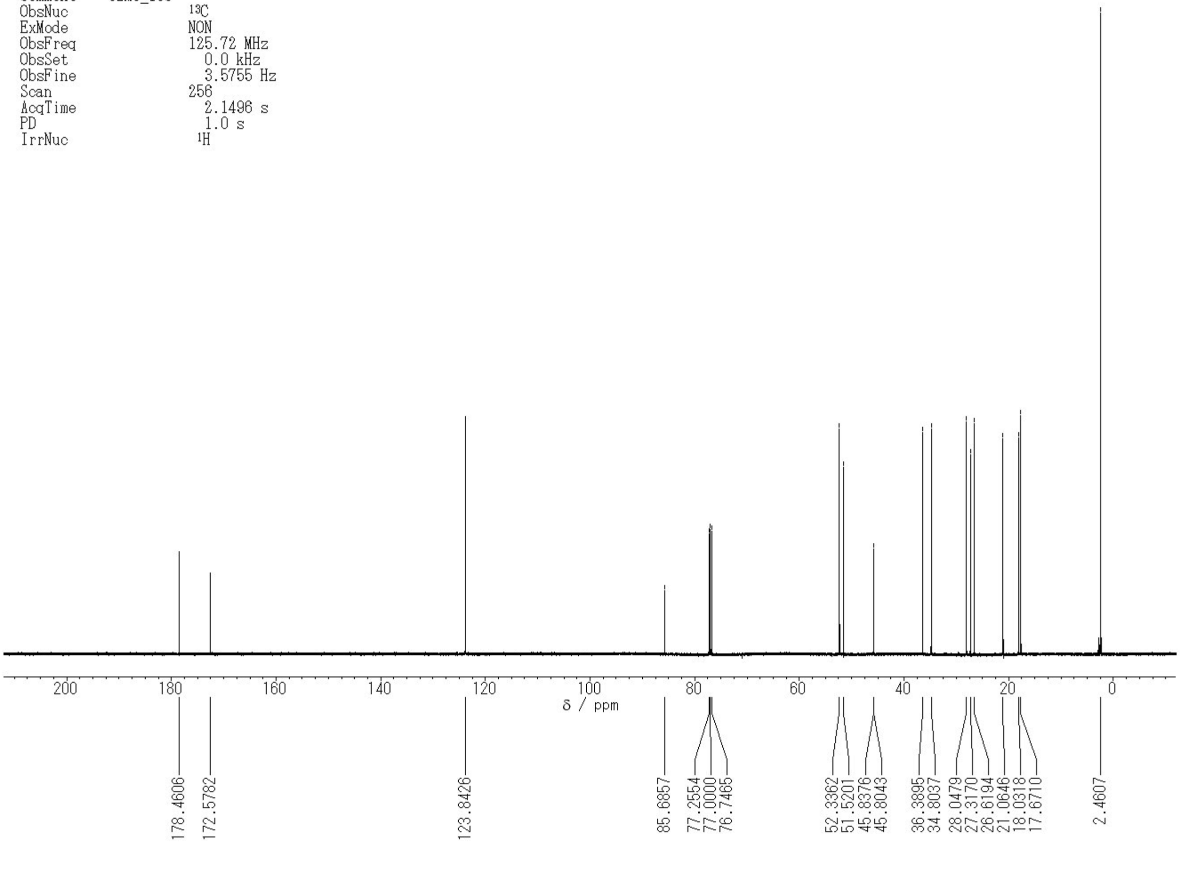

Comment 03KA_1H
ObsNuc 1H
ExMode NON
ObsFreq 499.92 MHz
ObsSet -1.0 kHz
ObsFine 985.6332 Hz
Scan 16
AcqTime 4.0894 s
PD 1.5 s
IrrNuc 13C

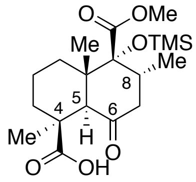

27

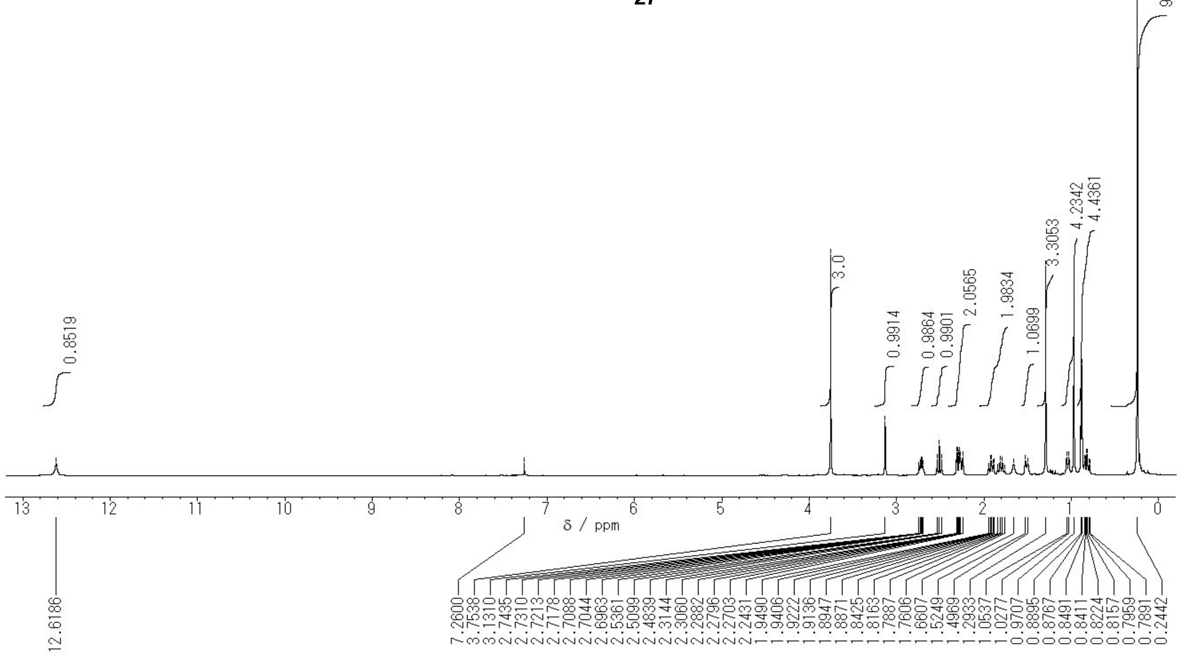

Comment 13KA_13C
ObsNuc 13C
ExMode NON
ObsFreq 125.72 MHz
ObsSet -1.0 kHz
ObsFine 997.238 Hz
Scan 512
AcqTime 2.1496 s
PD 1.0 s
IrrNuc 1H

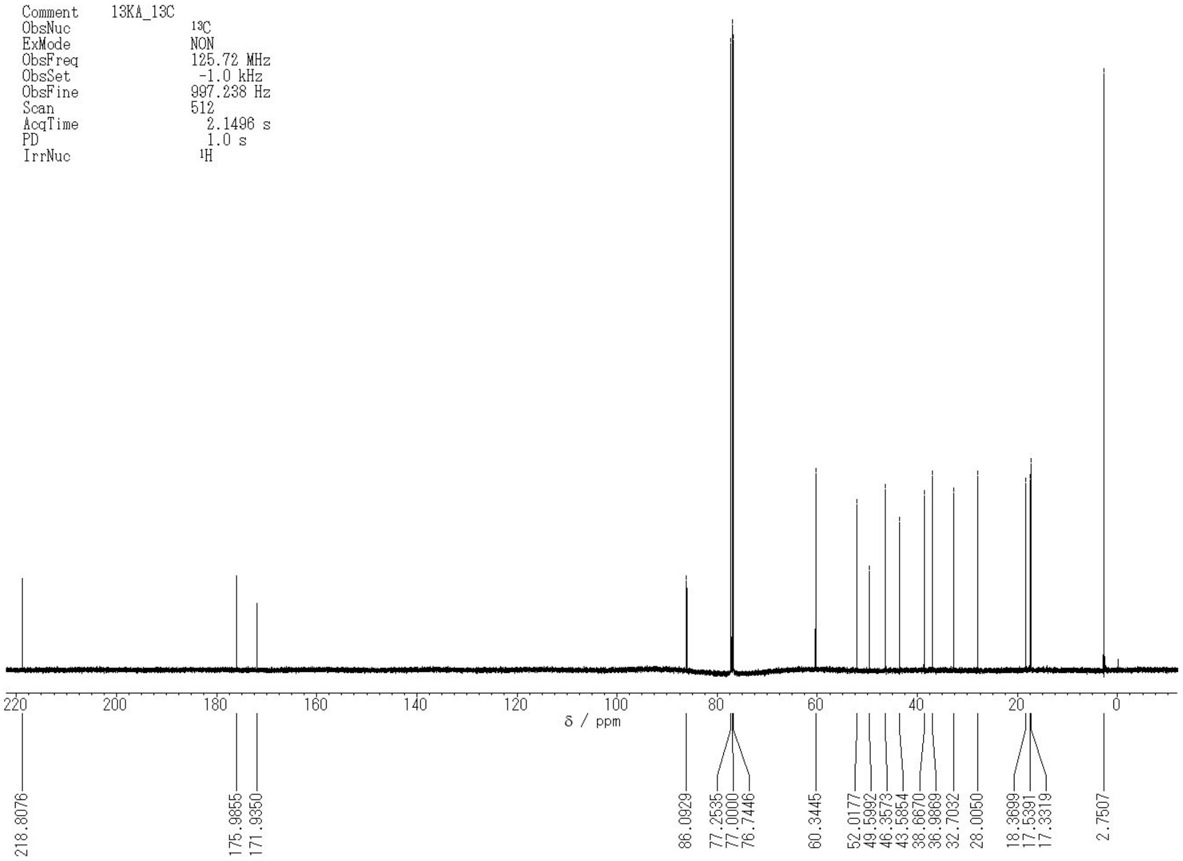

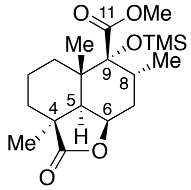
O
11
OMe
Me
OTMS
Me
9
8
5
6
4
H
Me
O
O

23

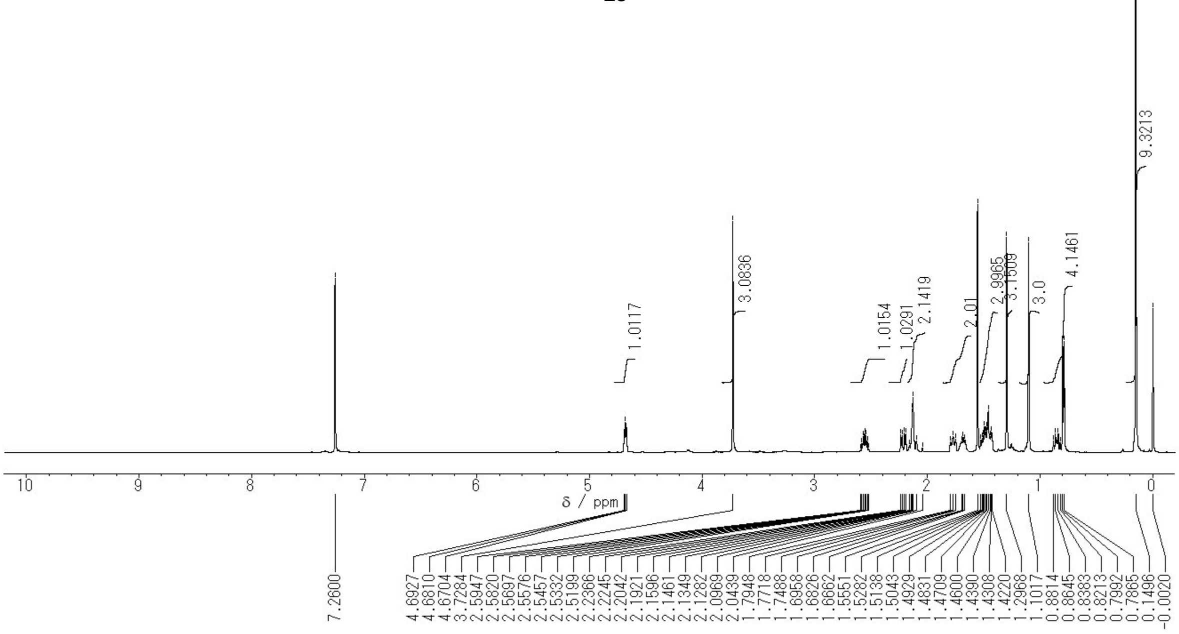
Comment 04lactone_1H
ObsNuc 1H
ExMode NON
ObsFreq 499.92 MHz
ObsSet -1.0 kHz
ObsFine 985.6332 Hz
Scan 16
AcqTime 4.0894 s
PD 1.5 s
IrrNuc 13C
9.3213
4.1461
3.0
3.1509
2.9965
2.01
2.1419
1.0291
1.0154
3.0836
1.0117
δ / ppm
7.2600
4.6927
4.6810
4.6704
3.7284
2.5947
2.5820
2.5697
2.5576
2.5457
2.5332
2.5199
2.2366
2.2245
2.2042
2.1921
2.1596
2.1461
2.1349
2.1282
2.0969
2.0439
1.7948
1.7718
1.7488
1.6958
1.6826
1.6662
1.5551
1.5282
1.5138
1.5043
1.4929
1.4831
1.4709
1.4600
1.4390
1.4308
1.4220
1.2968
1.1017
0.8814
0.8645
0.8383
0.8213
0.7992
0.7865
0.1496
-0.0020

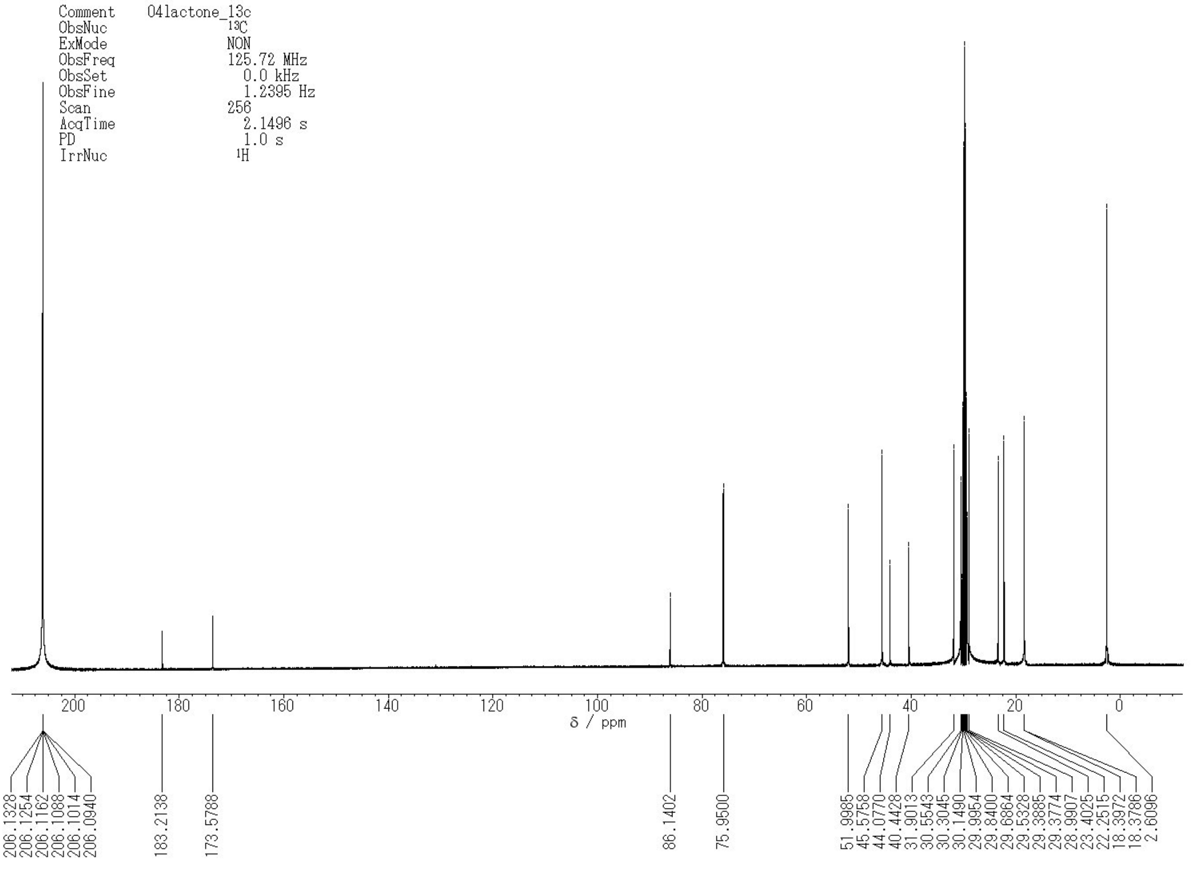
Comment 04lactone_13c
ObsNuc 13C
ExMode NON
ObsFreq 125.72 MHz
ObsSet 0.0 kHz
ObsFine 1.2395 Hz
Scan 256
AcqTime 2.1496 s
PD 1.0 s
IrrNuc 1H
δ / ppm
206.1328
206.1254
206.1162
206.1088
206.1014
206.0940
183.2138
173.5788
86.1402
75.9500
51.9985
45.5758
44.0770
40.4428
31.9013
30.5543
30.3045
30.1490
29.9954
29.8400
29.6864
29.5328
29.3885
29.3774
28.9907
23.4025
22.2515
18.3972
18.3786
2.6096

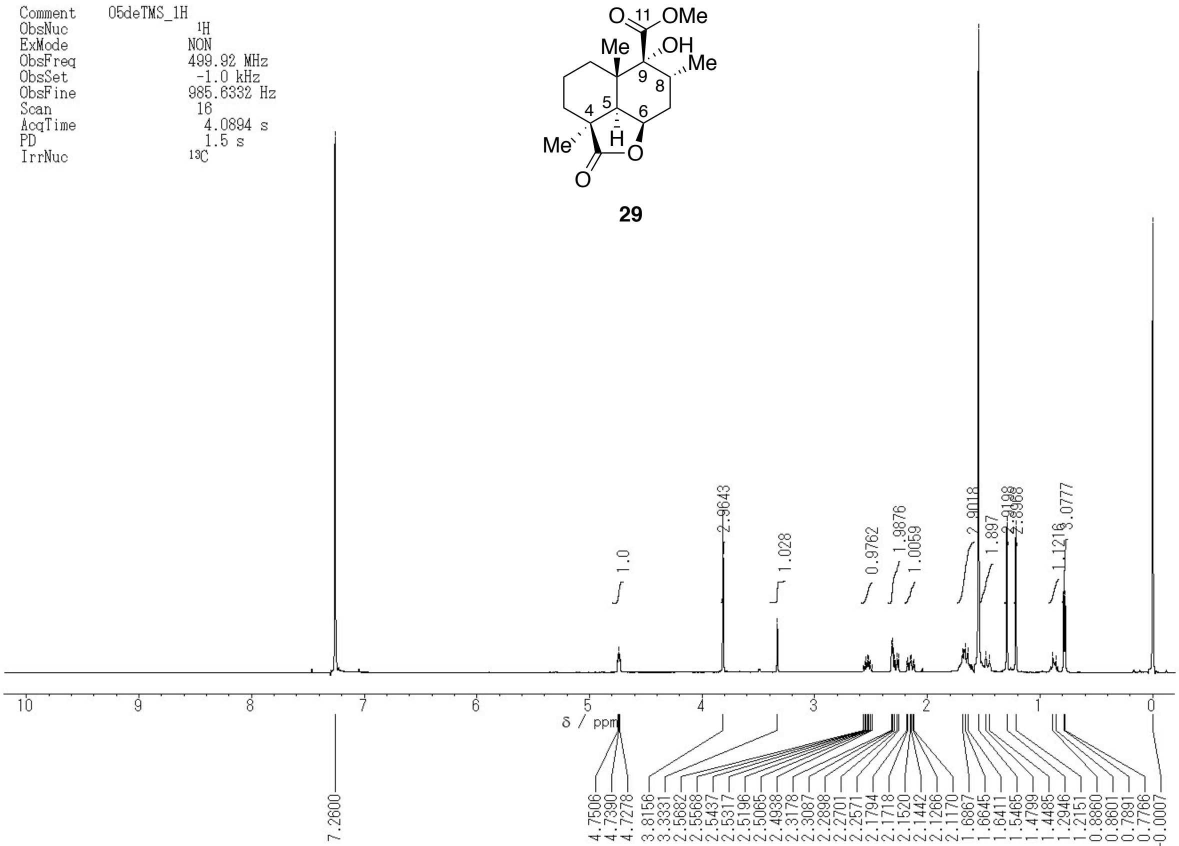

Comment 05deTMS_1H
ObsNuc 1H
ExMode NON
ObsFreq 499.92 MHz
ObsSet -1.0 kHz
ObsFine 985.6332 Hz
Scan 16
AcqTime 4.0894 s
PD 1.5 s
IrrNuc 13C
OMe
OH
Me
Me
Me
H
O
O
O
11
9
8
4
5
6
29
1.0
2.9643
1.028
0.9762
1.9876
1.0059
2.9018
1.897
1.1216
3.0777
δ / ppm
10
9
8
7
6
5
4
3
2
1
0
7.2600
4.7506
4.7390
4.7278
3.8156
3.3331
2.5682
2.5568
2.5437
2.5317
2.5196
2.5065
2.4938
2.3178
2.3087
2.2898
2.2701
2.2571
2.1794
2.1718
2.1520
2.1442
2.1266
2.1170
1.6867
1.6645
1.6411
1.5465
1.4799
1.4485
1.2946
1.2151
0.8860
0.8601
0.7891
0.7766
-0.0007

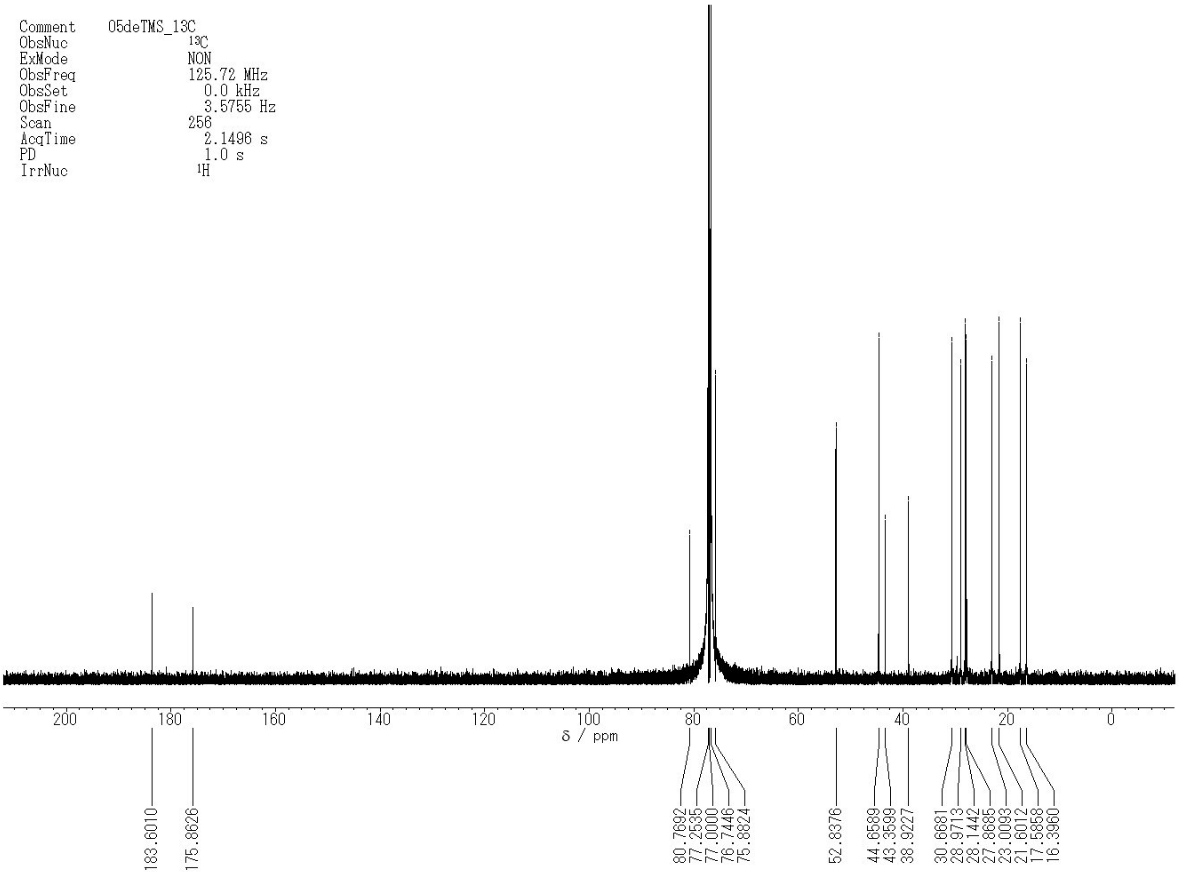

Comment 05deTMS_13C
ObsNuc 13C
ExMode NON
ObsFreq 125.72 MHz
ObsSet 0.0 kHz
ObsFine 3.5755 Hz
Scan 256
AcqTime 2.1496 s
PD 1.0 s
IrrNuc 1H
δ / ppm
200
180
160
140
120
100
80
60
40
20
0
183.6010
175.8626
80.7692
77.2535
77.0000
76.7446
75.8824
52.8376
44.6589
43.3599
38.9227
30.6681
28.9713
28.1442
27.8685
23.0093
21.6012
17.5858
16.3960

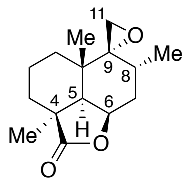
11
Me
O
9
8
Me
4
5
6
H
Me
O
O

22

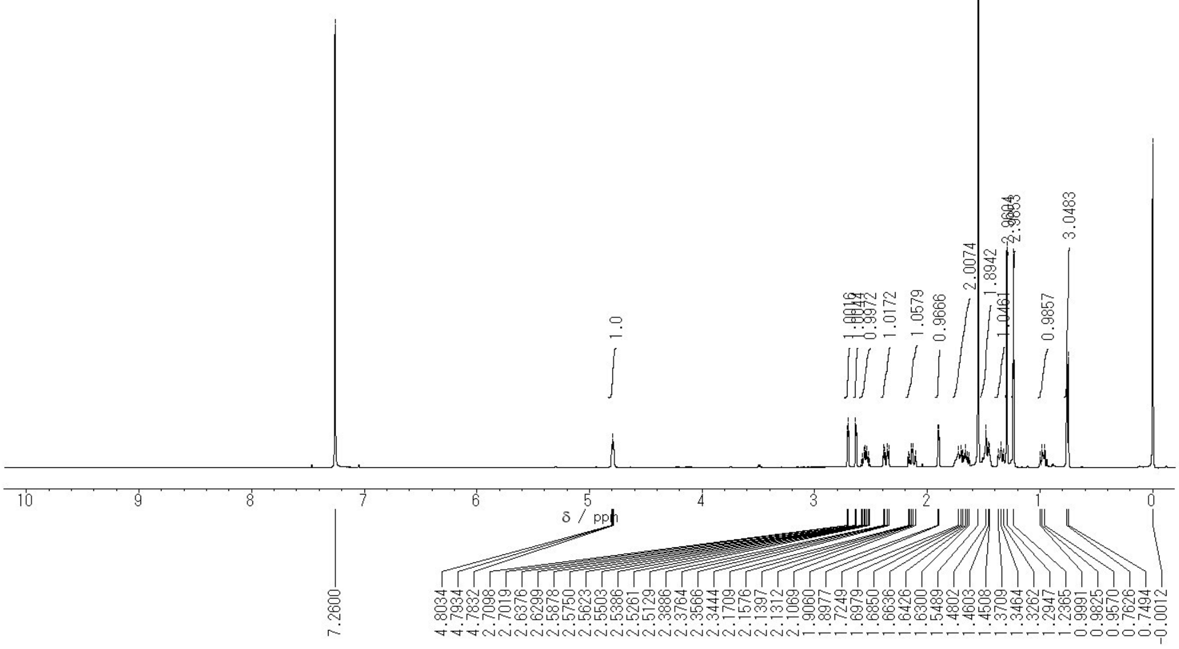
Comment 06epo_1H
ObsNuc 1H
ExMode NON
ObsFreq 499.92 MHz
ObsSet -1.0 kHz
ObsFine 985.6332 Hz
Scan 16
AcqTime 4.0894 s
PD 1.5 s
IrrNuc 13C
δ / ppm
1.0
0.9972
1.0172
1.0579
0.9666
2.0074
1.8942
1.0461
0.9857
3.0483
7.2600
4.8034
4.7934
4.7832
2.7098
2.7019
2.6376
2.6299
2.5878
2.5750
2.5623
2.5503
2.5386
2.5261
2.5129
2.3886
2.3764
2.3566
2.3444
2.1709
2.1576
2.1397
2.1312
2.1069
1.9060
1.8977
1.7249
1.6979
1.6850
1.6636
1.6426
1.6300
1.5489
1.4802
1.4603
1.4508
1.3709
1.3464
1.3262
1.2947
1.2365
0.9991
0.9825
0.9570
0.7626
0.7494
-0.0012

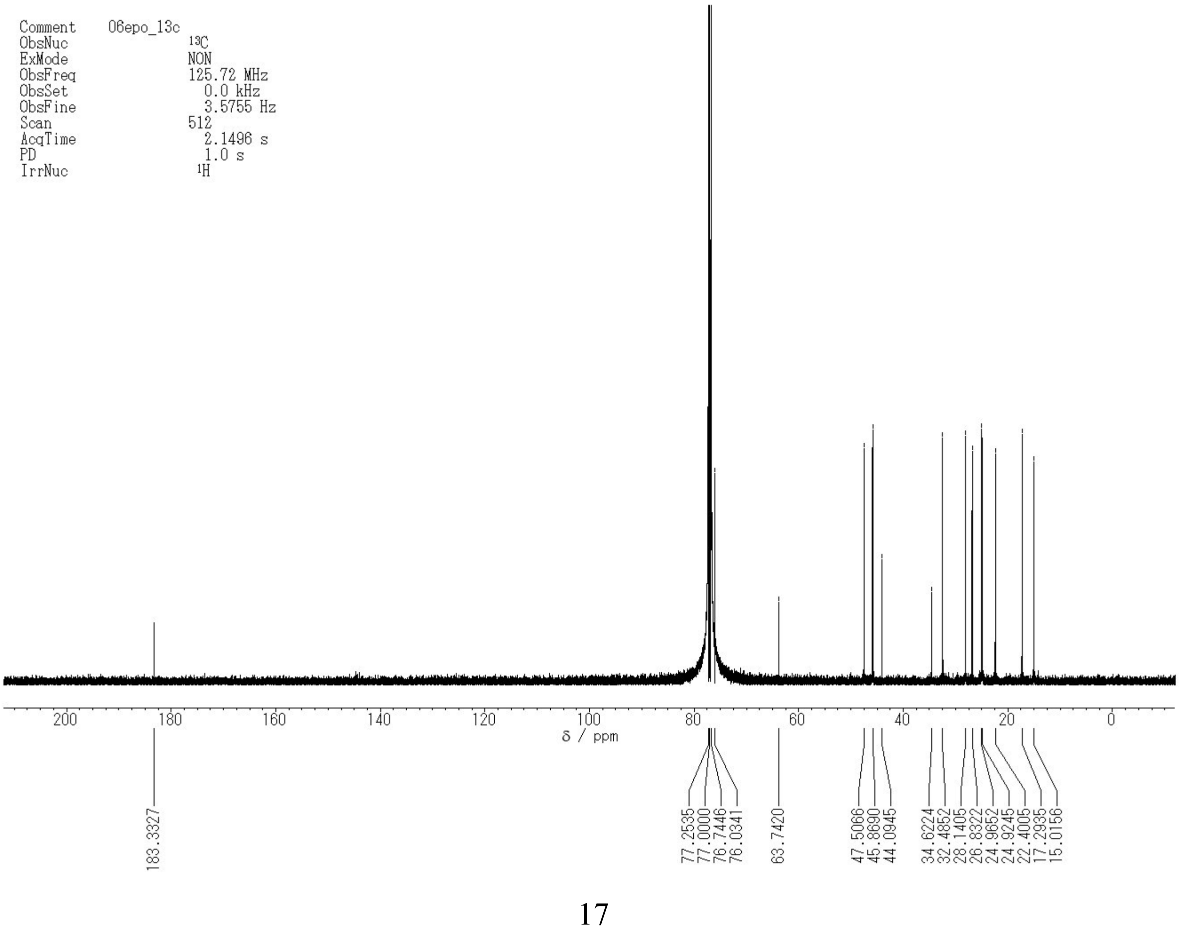
Comment 06epo_13c
ObsNuc 13C
ExMode NON
ObsFreq 125.72 MHz
ObsSet 0.0 kHz
ObsFine 3.5755 Hz
Scan 512
AcqTime 2.1496 s
PD 1.0 s
IrrNuc 1H
δ / ppm
183.3327
77.2535
77.0000
76.7446
76.0341
63.7420
47.5066
45.8690
44.0945
34.6224
32.4852
28.1405
26.8322
24.9652
24.9245
22.4005
17.2935
15.0156

Comment 07marrubiin_1H
ObsNuc 1H
ExMode NON
ObsFreq 499.92 MHz
ObsSet 0.0 kHz
ObsFine 13.8008 Hz
Scan 8
AcqTime 4.0894 s
PD 1.5 s
IrrNuc 13C

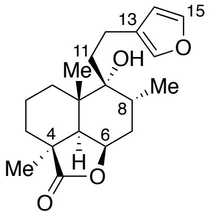

marrubiin (**1**)

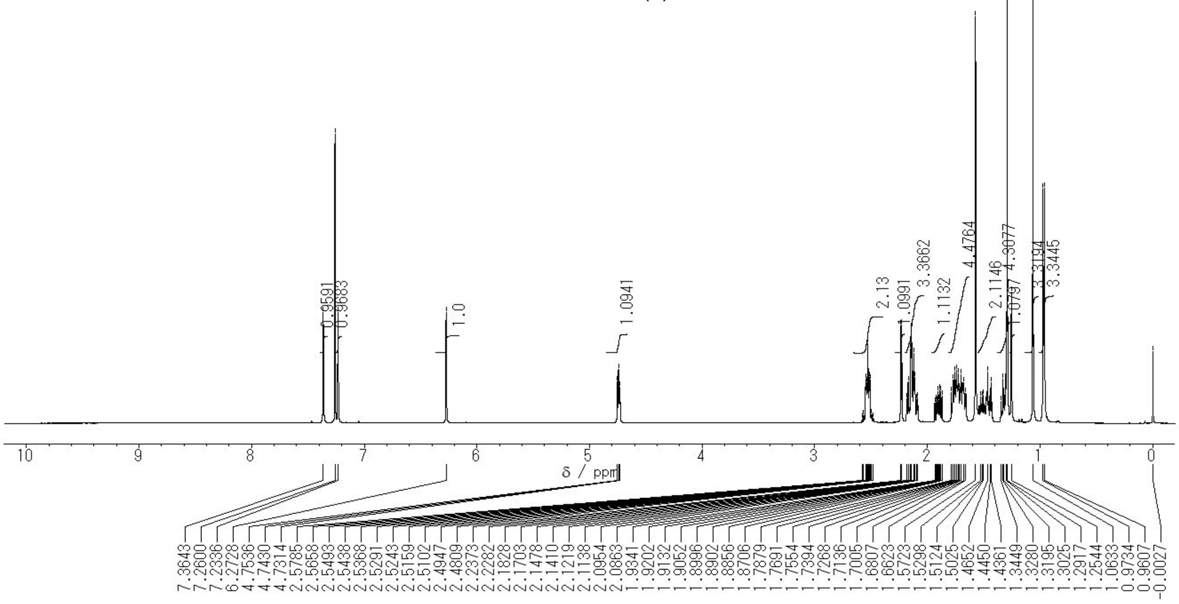

Comment 07marrubiin_13c
ObsNuc 13C
ExMode NON
ObsFreq 125.72 MHz
ObsSet 0.0 kHz
ObsFine 3.5755 Hz
Scan 2000
AcqTime 2.1496 s
PD 1.0 s
IrrNuc 1H

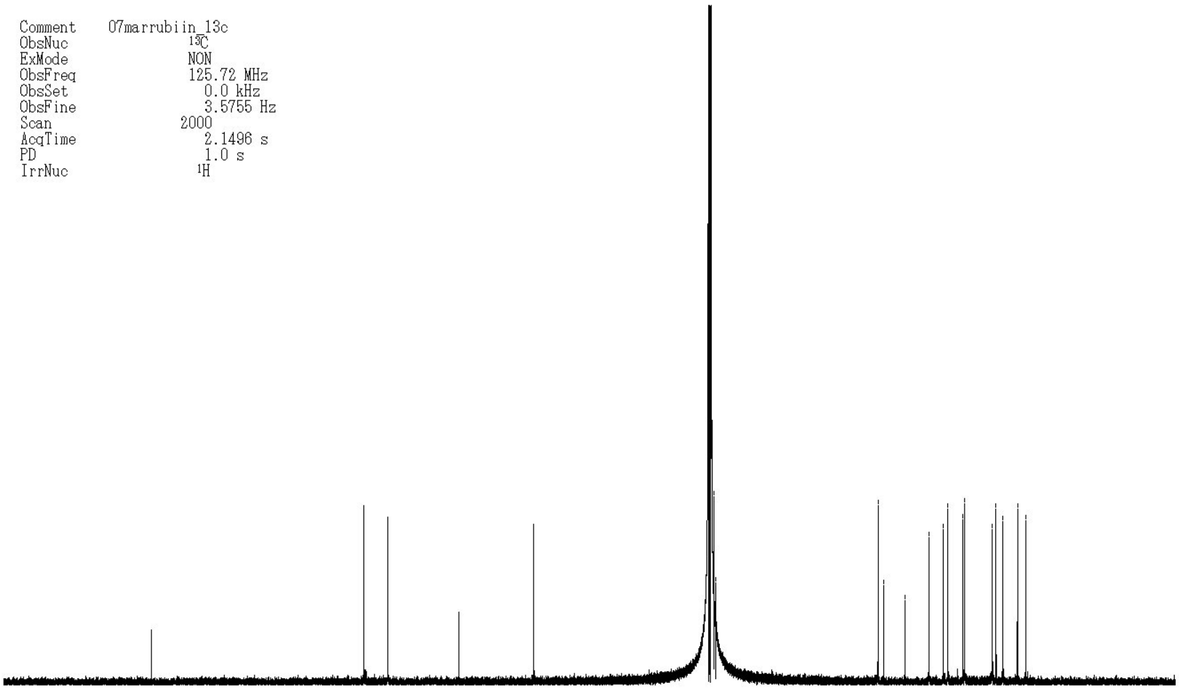

Comment 08_marrubiin_iso_1H
ObsNuc 1H
ExMode single_pulse.jxp
ObsFreq 500.0 MHz
ObsSet 162.0 kHz
ObsFine 416.009 Hz
Scan 8
AcqTime 1.7459 s
PD 5.0 s
IrrNuc NUL

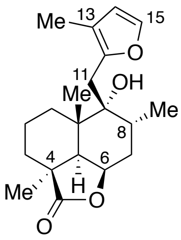

33

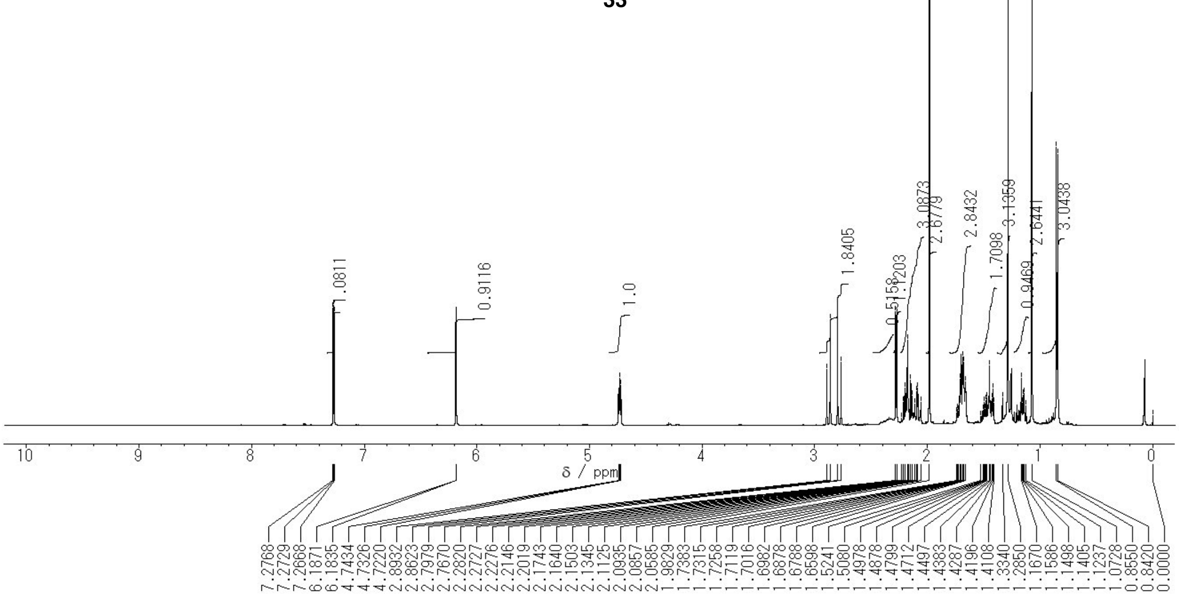

Comment 08_marrubiin_iso_13C
ObsNuc 13C
ExMode CARBON
ObsFreq 125.66 MHz
ObsSet -1.0 kHz
ObsFine 997.2565 Hz
Scan 256
AcqTime 1.0486 s
PD 1.9514 s
IrrNuc 1H

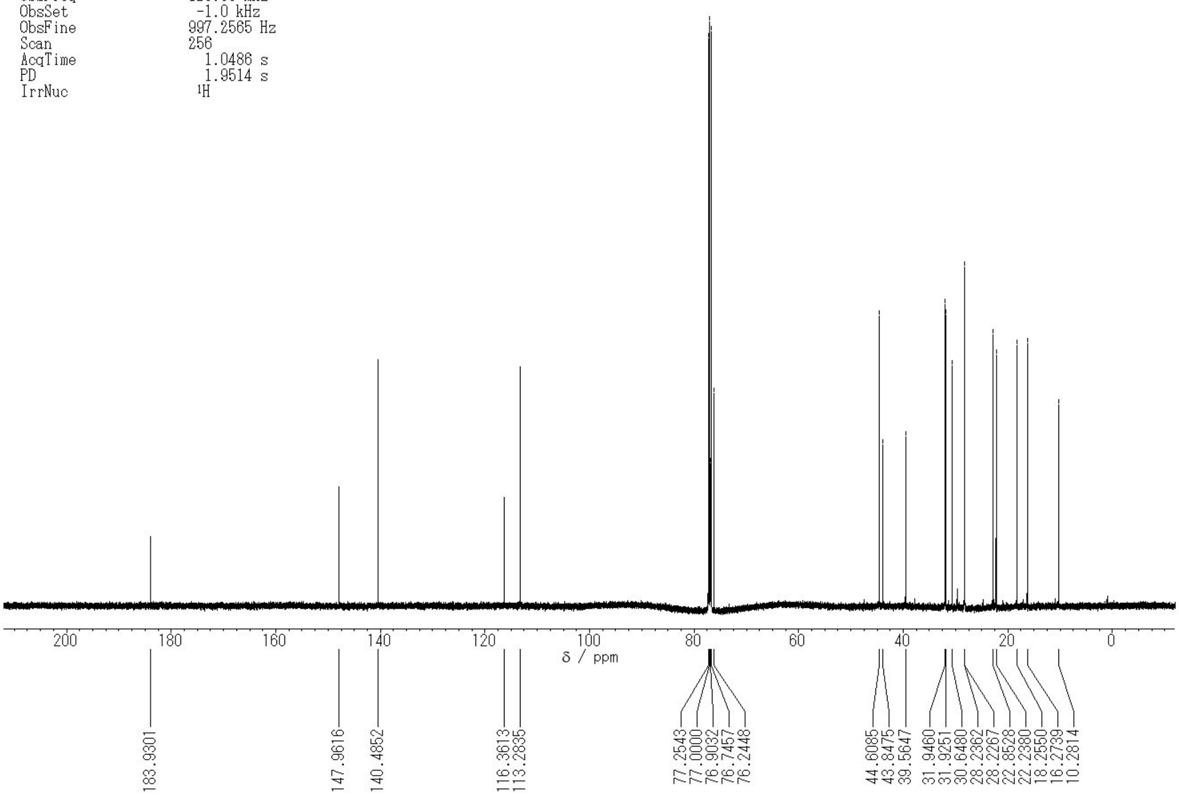

Comment 09_TMSmarrubiin_1H
ObsNuc 1H
ExMode single_pulse.jxp
ObsFreq 500.0 MHz
ObsSet 162.0 kHz
ObsFine 416.009 Hz
Scan 8
AcqTime 1.7459 s
PD 5.0 s
IrrNuc ??

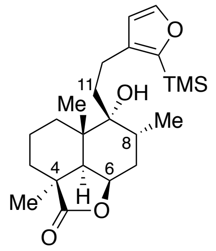

35

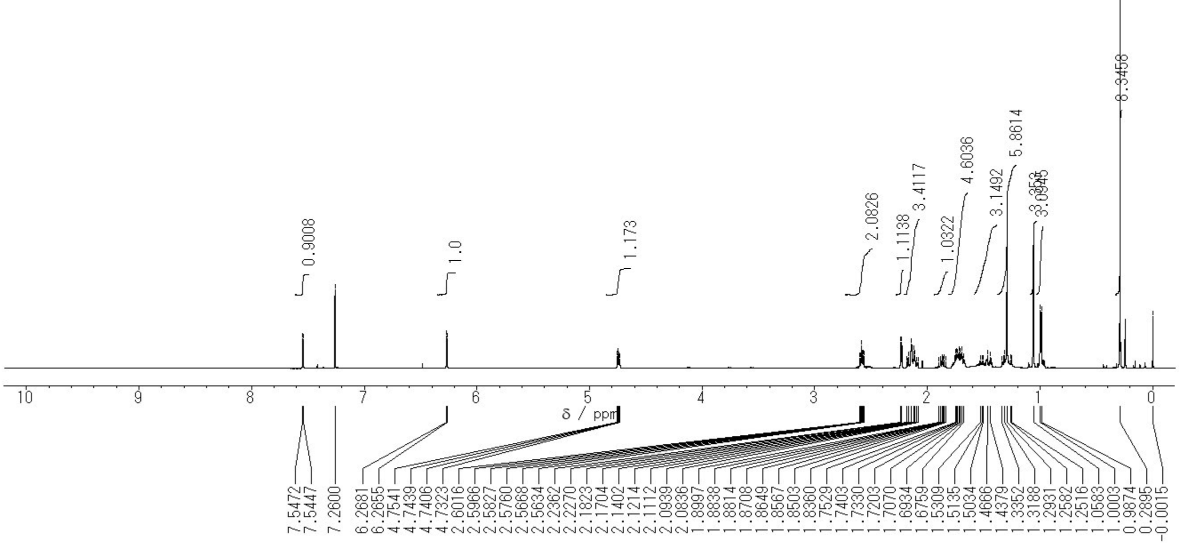

Comment 09_TMSmarrubiin_13C
ObsNuc 13C
ExMode CARBON
ObsFreq 125.72 MHz
ObsSet 0.0 kHz
ObsFine 0.1276 Hz
Scan 128
AcqTime 1.0486 s
PD 1.9514 s
IrrNuc 1H

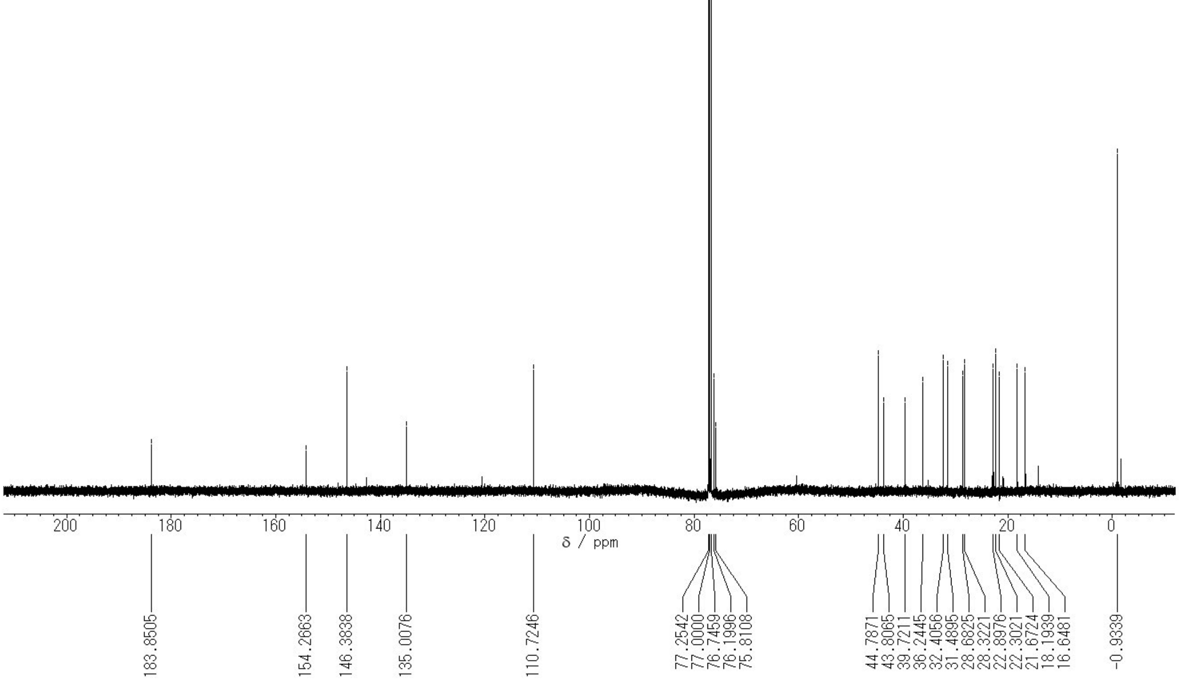

Comment 10bisOEt_1H
ObsNuc ^{1}H
ExMode NON
ObsFreq 499.92 MHz
ObsSet 0.0 kHz
ObsFine 13.8008 Hz
Scan 8
AcqTime 4.0894 s
PD 1.5 s
IrrNuc ^{13}C

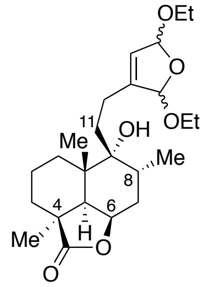

36

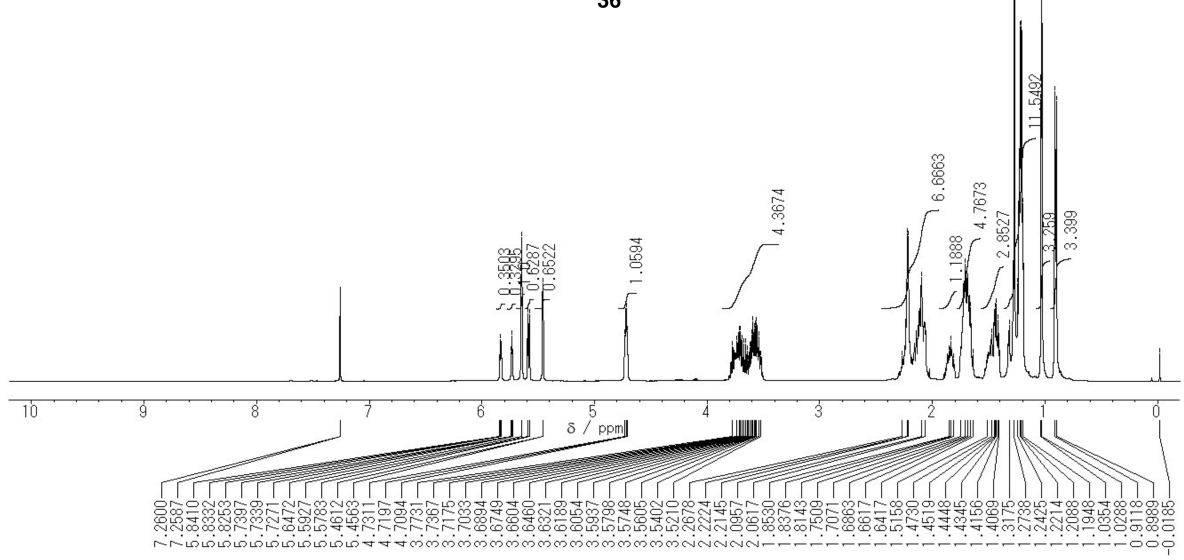

Comment 10bisOEt_13C
ObsNuc ^{13}C
ExMode NON
ObsFreq 125.72 MHz
ObsSet -1.0 kHz
ObsFine 997.238 Hz
Scan 64
AcqTime 2.1496 s
PD 1.0 s
IrrNuc ^{1}H

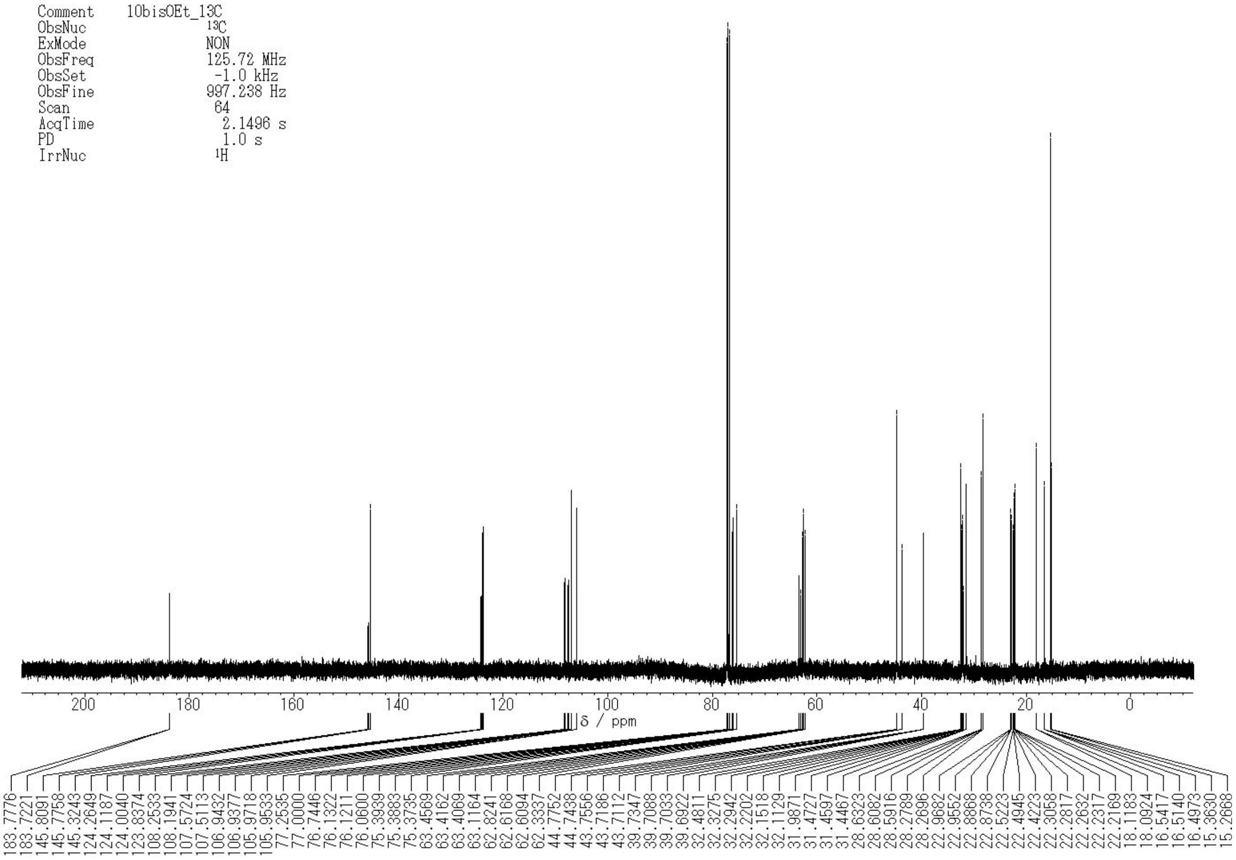

Comment 110s04Et_1H
ObsNuc ^{1}H
ExMode NON
ObsFreq 499.92 MHz
ObsSet 0.0 kHz
ObsFine 13.8008 Hz
Scan 8
AcqTime 4.0894 s
PD 1.5 s
IrrNuc ^{13}C

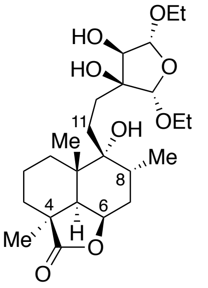

38

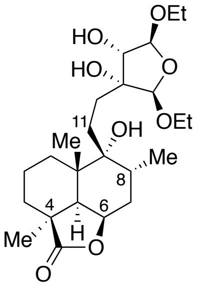

39

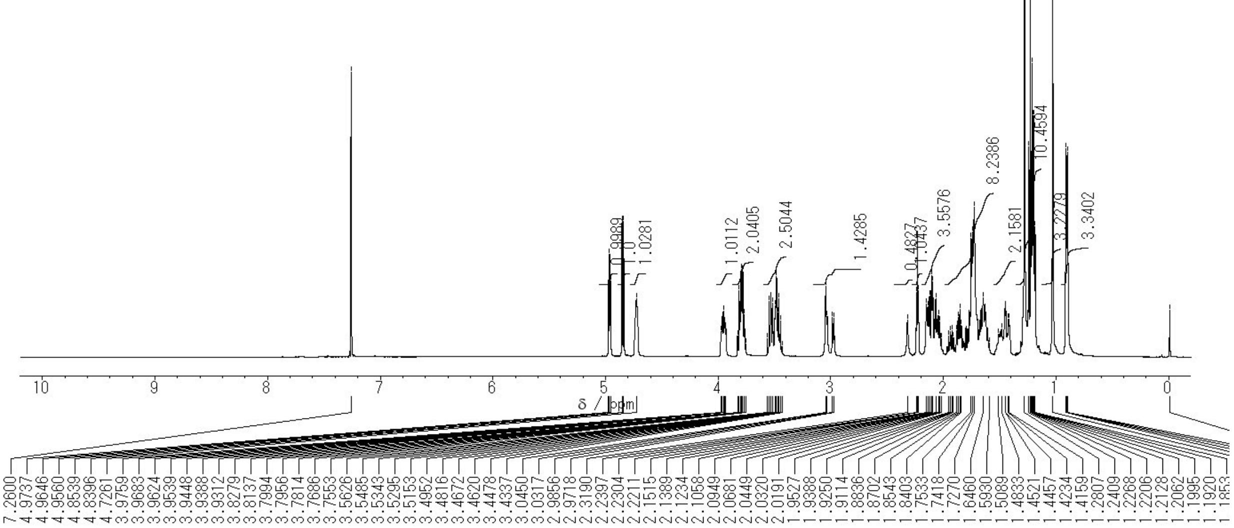

Comment 110s04Et_13c
ObsNuc ^{13}C
ExMode NON
ObsFreq 125.72 MHz
ObsSet -1.0 kHz
ObsFine 997.238 Hz
Scan 512
AcqTime 2.1496 s
PD 1.0 s
IrrNuc ^{1}H

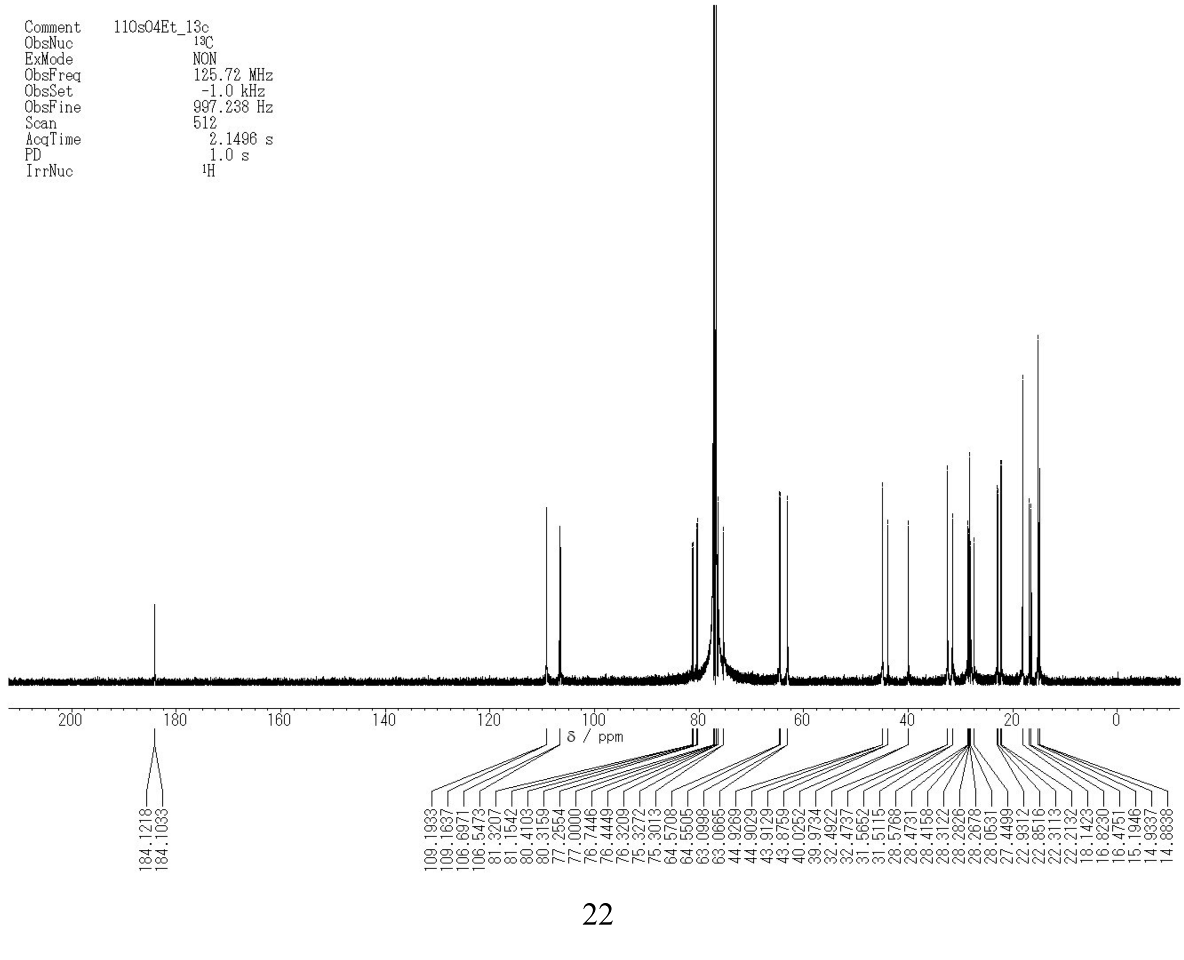

```
Comment     12marrulibacetal_1H
ObsNuc           ¹H
ExMode           NON
ObsFreq          499.92 MHz
ObsSet             0.0 kHz
ObsFine          13.8008 Hz
Scan             16
AcqTime            4.0894 s
PD                 1.5 s
IrrNuc           ¹³C
```

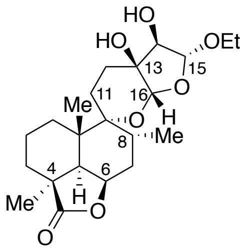

marrulibacetal (**13**)

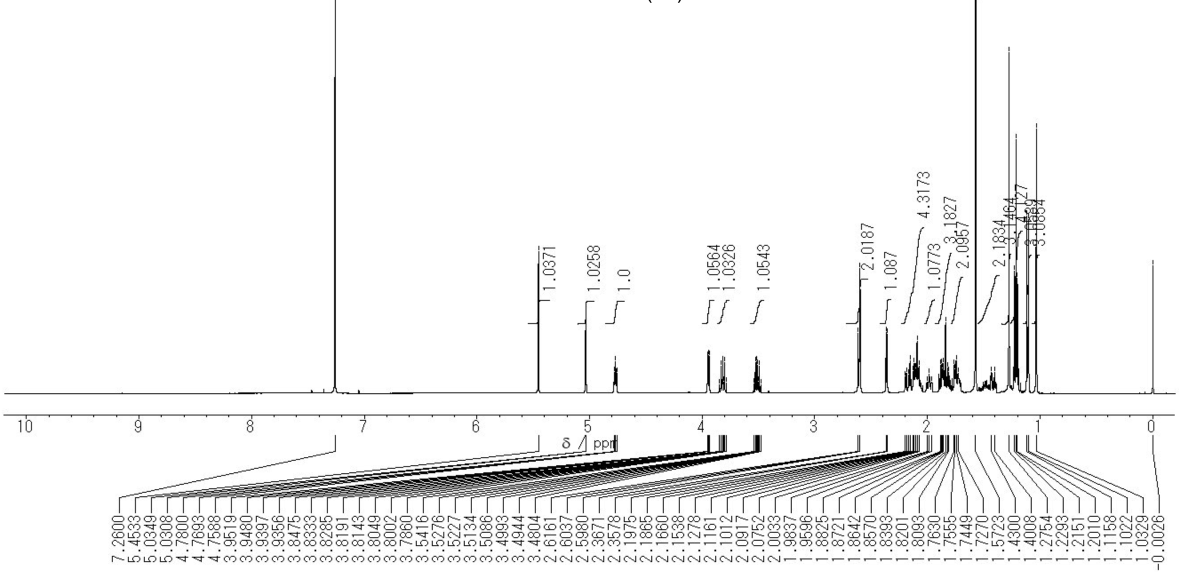


```
Comment     12_marrulibacetal_13C
ObsNuc           ¹³C
ExMode           NON
ObsFreq          125.72 MHz
ObsSet            -1.0 kHz
ObsFine          997.238 Hz
Scan             5000
AcqTime            2.1496 s
PD                 1.0 s
IrrNuc           ¹H
```

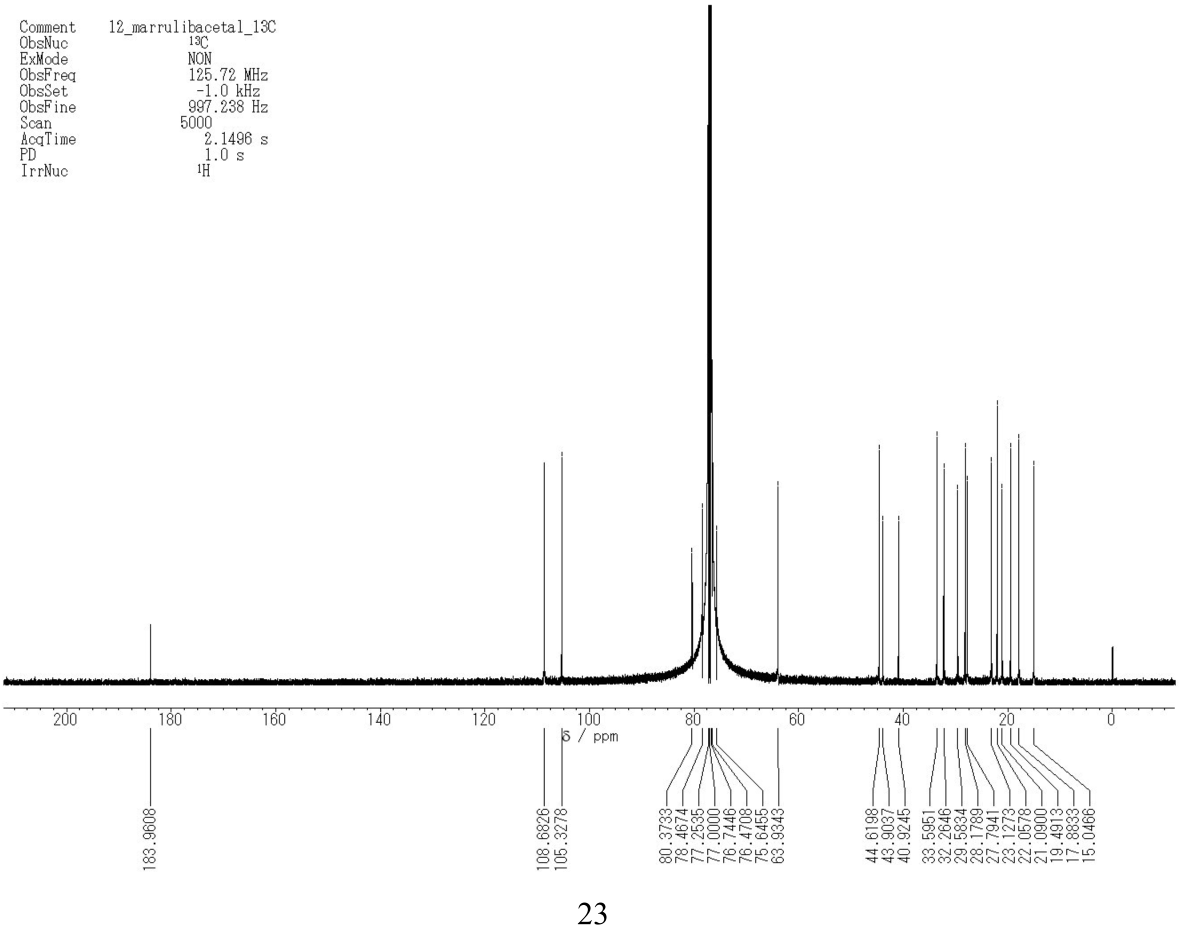

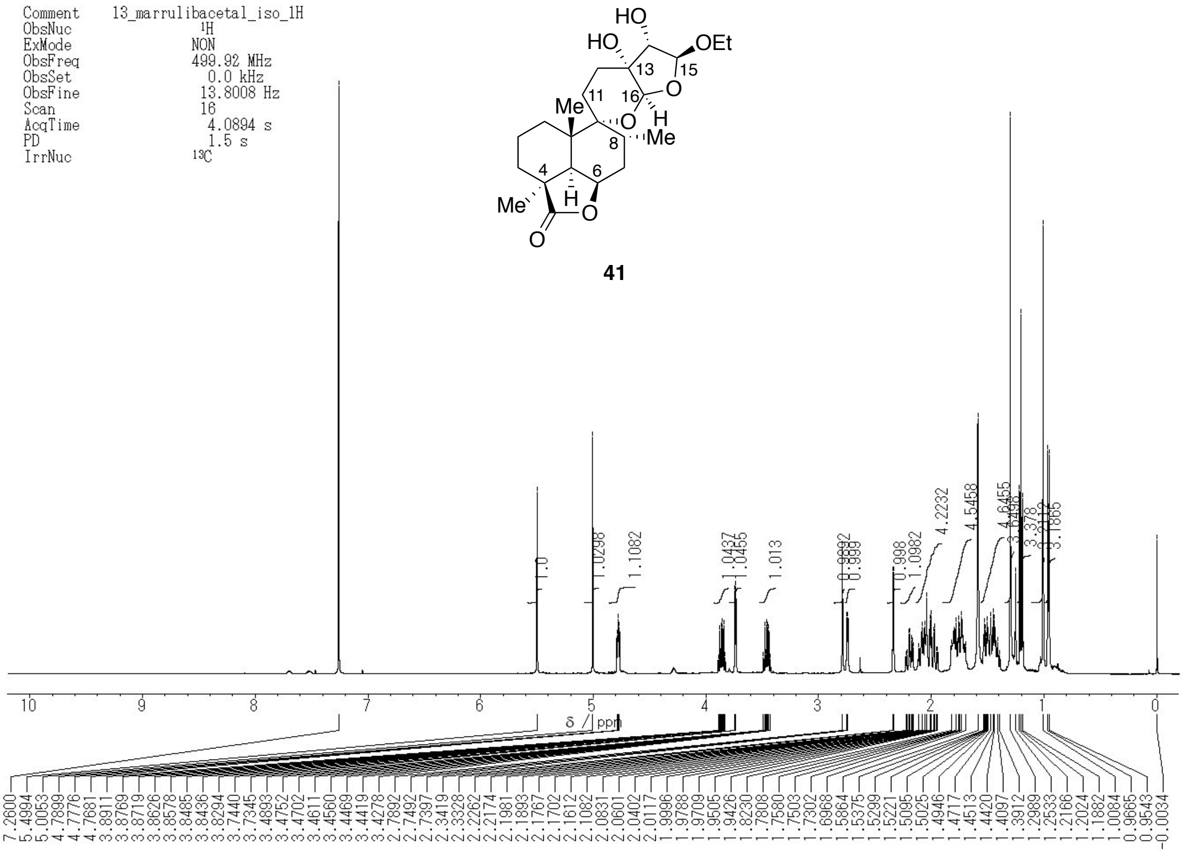
Comment 13_marrulibacetal_iso_1H
ObsNuc 1H
ExMode NON
ObsFreq 499.92 MHz
ObsSet 0.0 kHz
ObsFine 13.8008 Hz
Scan 16
AcqTime 4.0894 s
PD 1.5 s
IrrNuc 13C
HO
HO
OEt
13
15
11
16
O
Me
H
O
8
Me
4
6
H
Me
O
O
41
δ / ppm

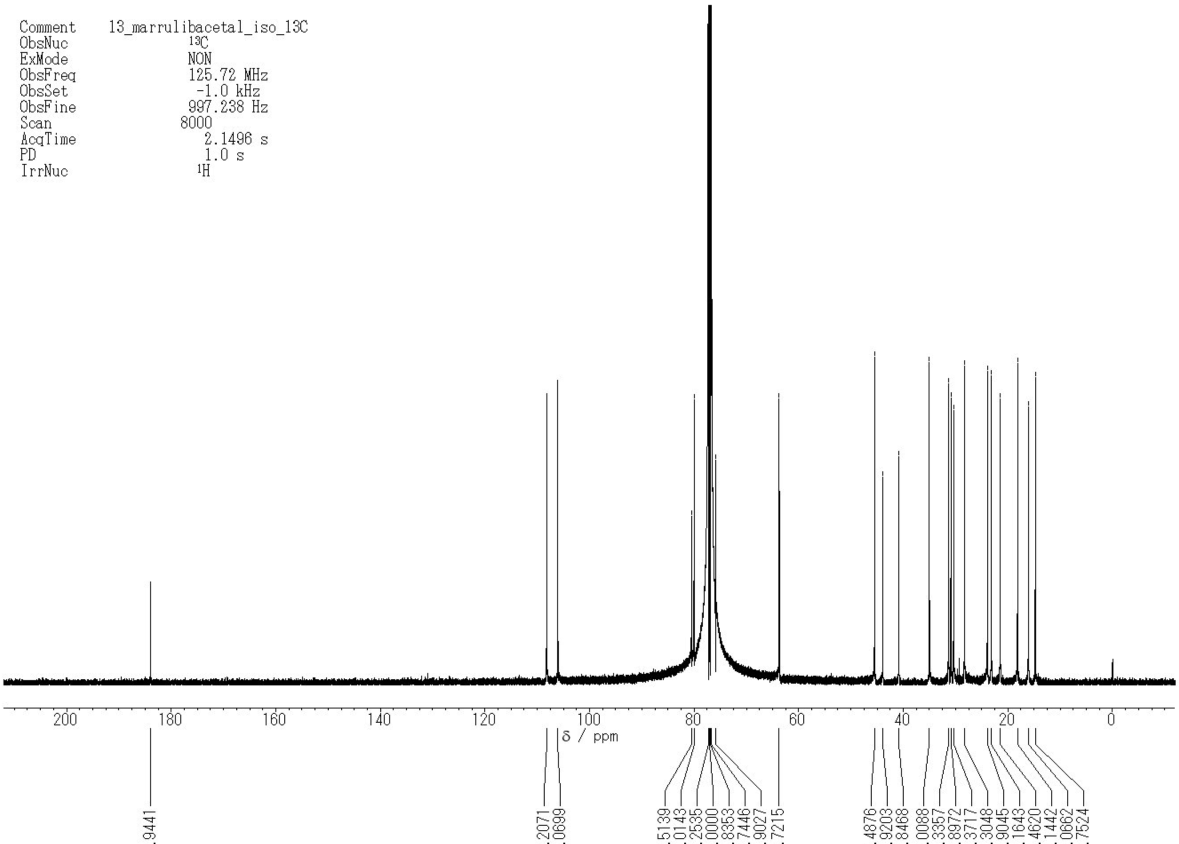
Comment 13_marrulibacetal_iso_13C
ObsNuc 13C
ExMode NON
ObsFreq 125.72 MHz
ObsSet -1.0 kHz
ObsFine 997.238 Hz
Scan 8000
AcqTime 2.1496 s
PD 1.0 s
IrrNuc 1H
δ / ppm

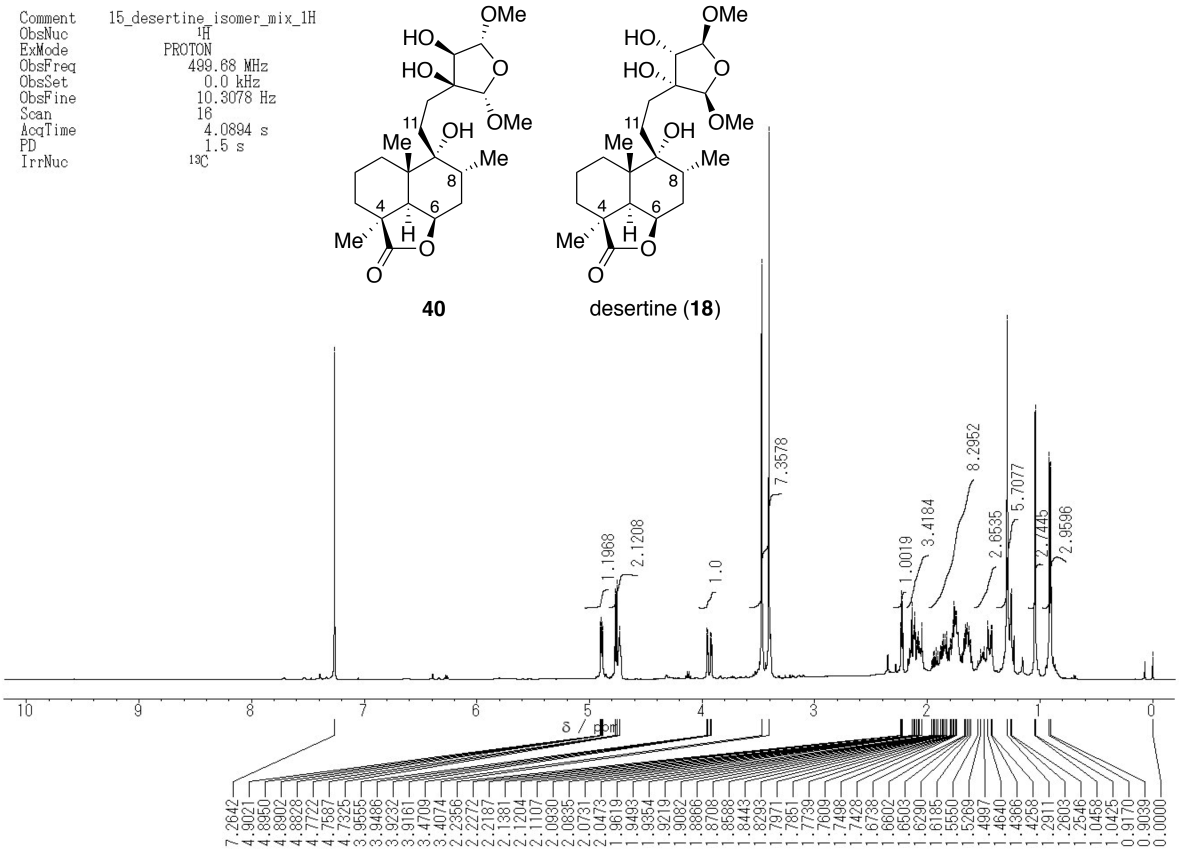
Comment 15_desertine_isomer_mix_1H
ObsNuc 1H
ExMode PROTON
ObsFreq 499.68 MHz
ObsSet 0.0 kHz
ObsFine 10.3078 Hz
Scan 16
AcqTime 4.0894 s
PD 1.5 s
IrrNuc 13C
40
desertine (18)
1.1968
2.1208
1.0
7.3578
1.0019
3.4184
8.2952
2.6535
5.7077
2.7445
2.9596
δ / ppm
7.2642 4.9021 4.8950 4.8902 4.8828 4.7722 4.7587 4.7325 3.9655 3.9486 3.9232 3.9161 3.4709 3.4074 2.2356 2.2272 2.2187 2.1381 2.1204 2.1107 2.0930 2.0835 2.0731 2.0473 1.9619 1.9493 1.9354 1.9219 1.9082 1.8866 1.8708 1.8588 1.8443 1.8293 1.7971 1.7851 1.7739 1.7609 1.7498 1.7428 1.6738 1.6602 1.6503 1.6290 1.6185 1.5550 1.5269 1.4997 1.4640 1.4366 1.4258 1.2911 1.2603 1.2546 1.0458 1.0425 0.9170 0.9039 0.0000

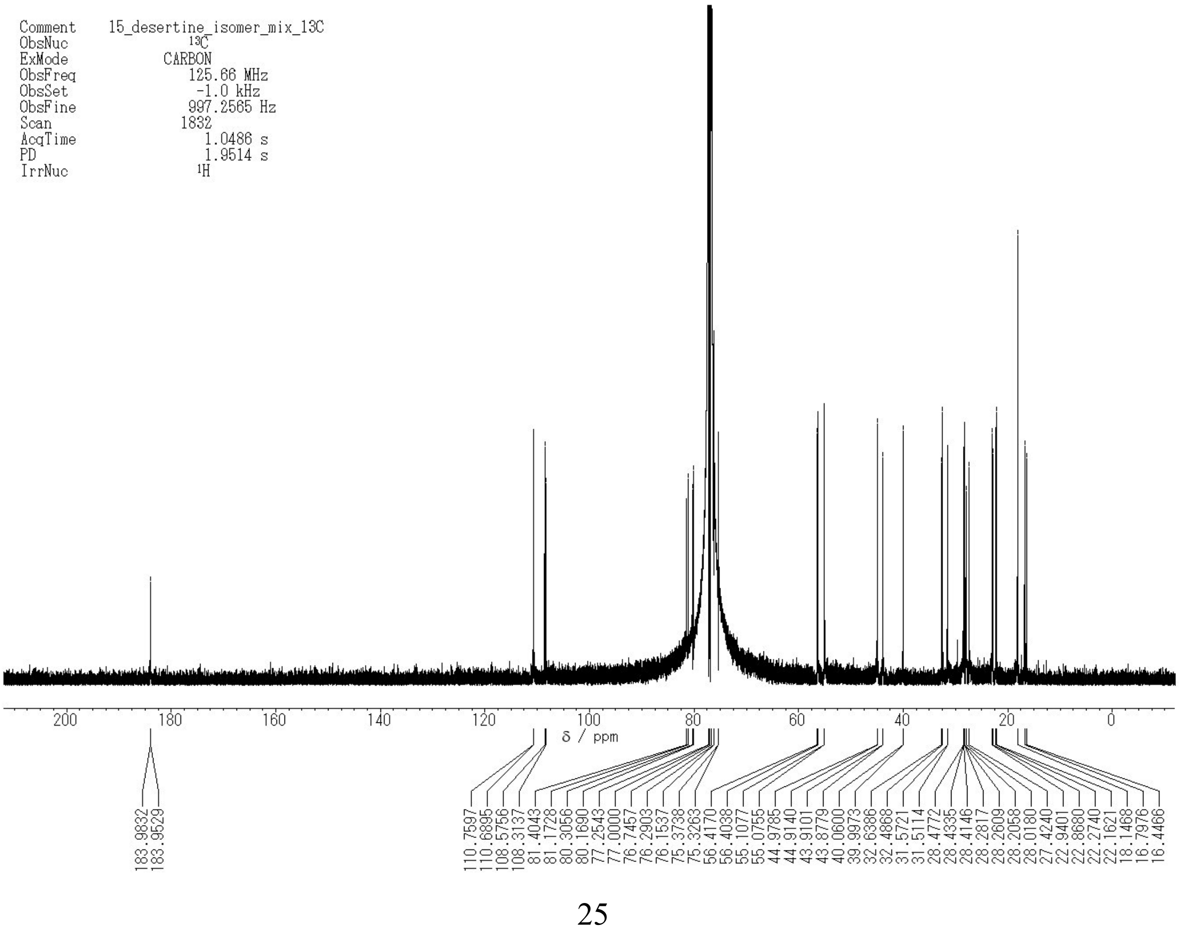
Comment 15_desertine_isomer_mix_13C
ObsNuc 13C
ExMode CARBON
ObsFreq 125.66 MHz
ObsSet -1.0 kHz
ObsFine 997.2565 Hz
Scan 1832
AcqTime 1.0486 s
PD 1.9514 s
IrrNuc 1H
δ / ppm
183.9832 183.9629
110.7597 110.6895 108.5756 108.3137 81.4043 81.1728 80.3056 80.1690 77.2543 77.0000 76.7457 76.2903 76.1537 75.3738 75.3263 56.4170 56.4038 55.1077 55.0755 44.9785 44.9140 43.9101 43.8779 40.0600 39.9973 32.6386 32.4868 31.5721 31.5114 28.4772 28.4335 28.4146 28.2817 28.2609 28.2058 28.0180 27.4240 22.9401 22.8680 22.2740 22.1621 18.1468 16.7976 16.4466

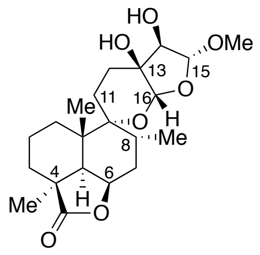

marrulibacetal A (**17**)

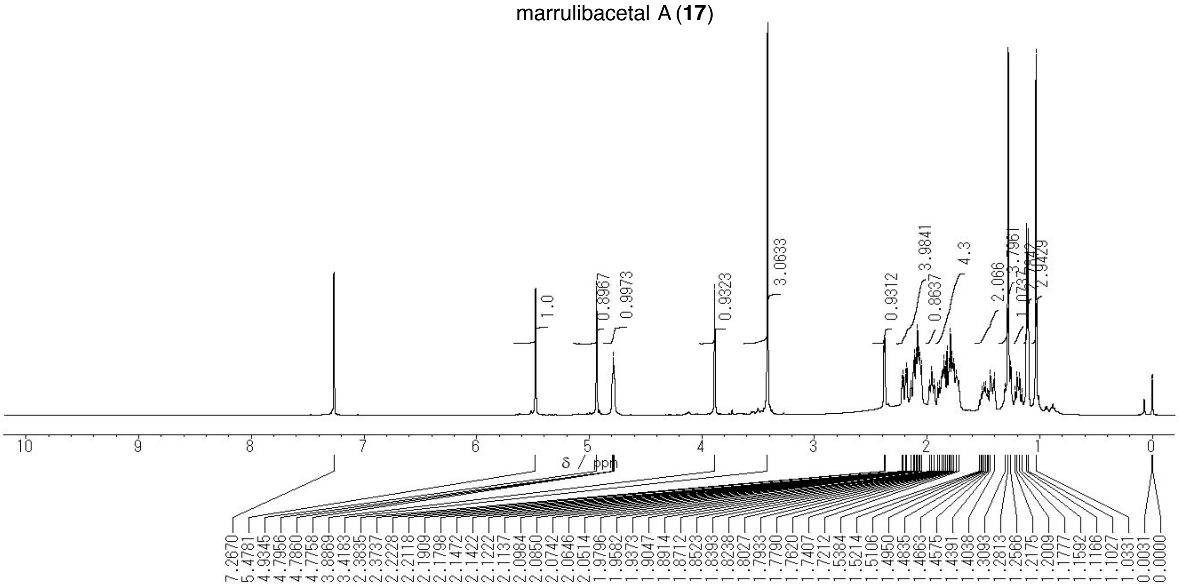

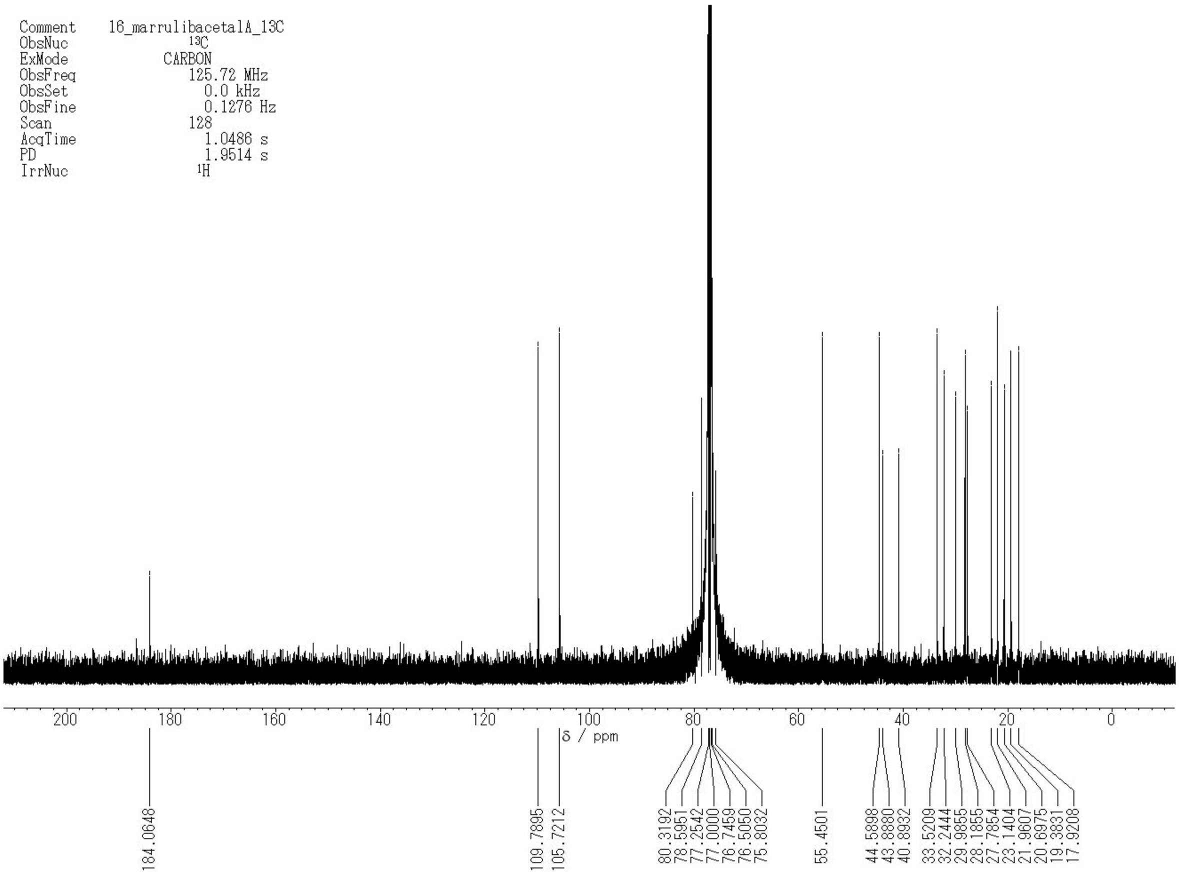

Comment 17_marrulibacetalA_iso_1H
ObsNuc ^{1}H
ExMode PROTON
ObsFreq 499.92 MHz
ObsSet 0.0 kHz
ObsFine 13.8008 Hz
Scan 4
AcqTime 4.0894 s
PD 1.5 s
IrrNuc ^{13}C

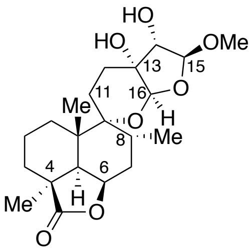

42

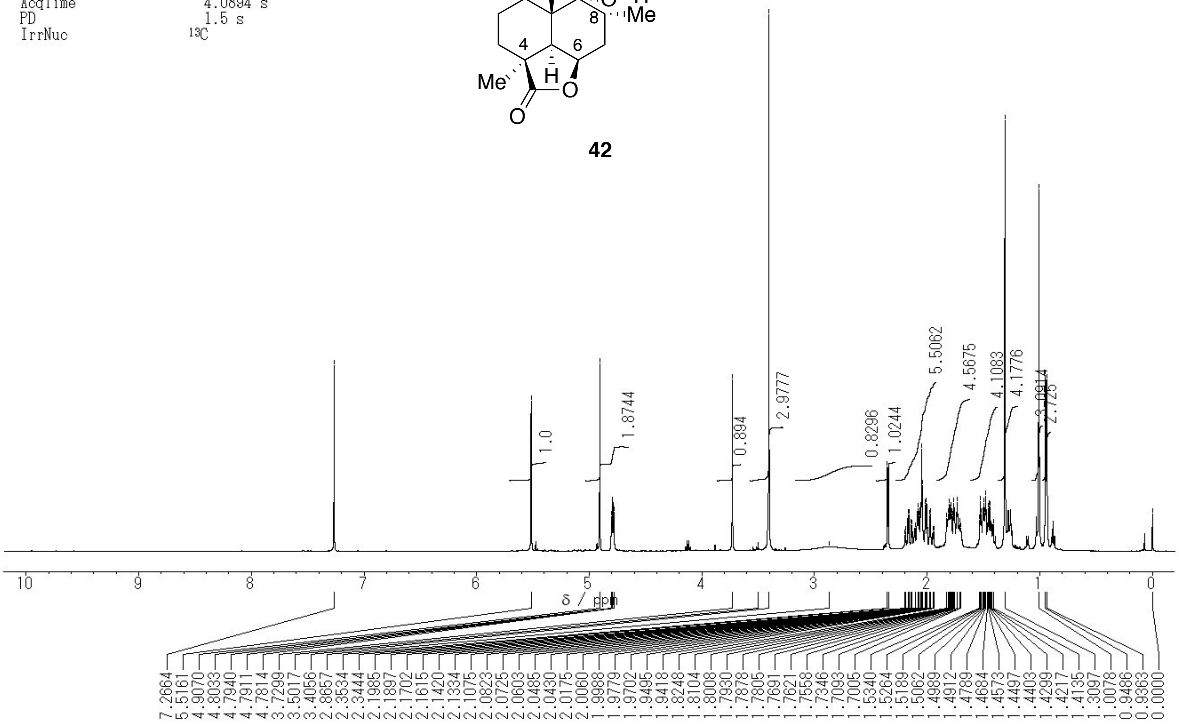

Comment 17_marrulibacetalA_iso_13C
ObsNuc ^{13}C
ExMode CARBON
ObsFreq 125.72 MHz
ObsSet 0.0 kHz
ObsFine 0.1276 Hz
Scan 128
AcqTime 1.0486 s
PD 1.9514 s
IrrNuc ^{1}H

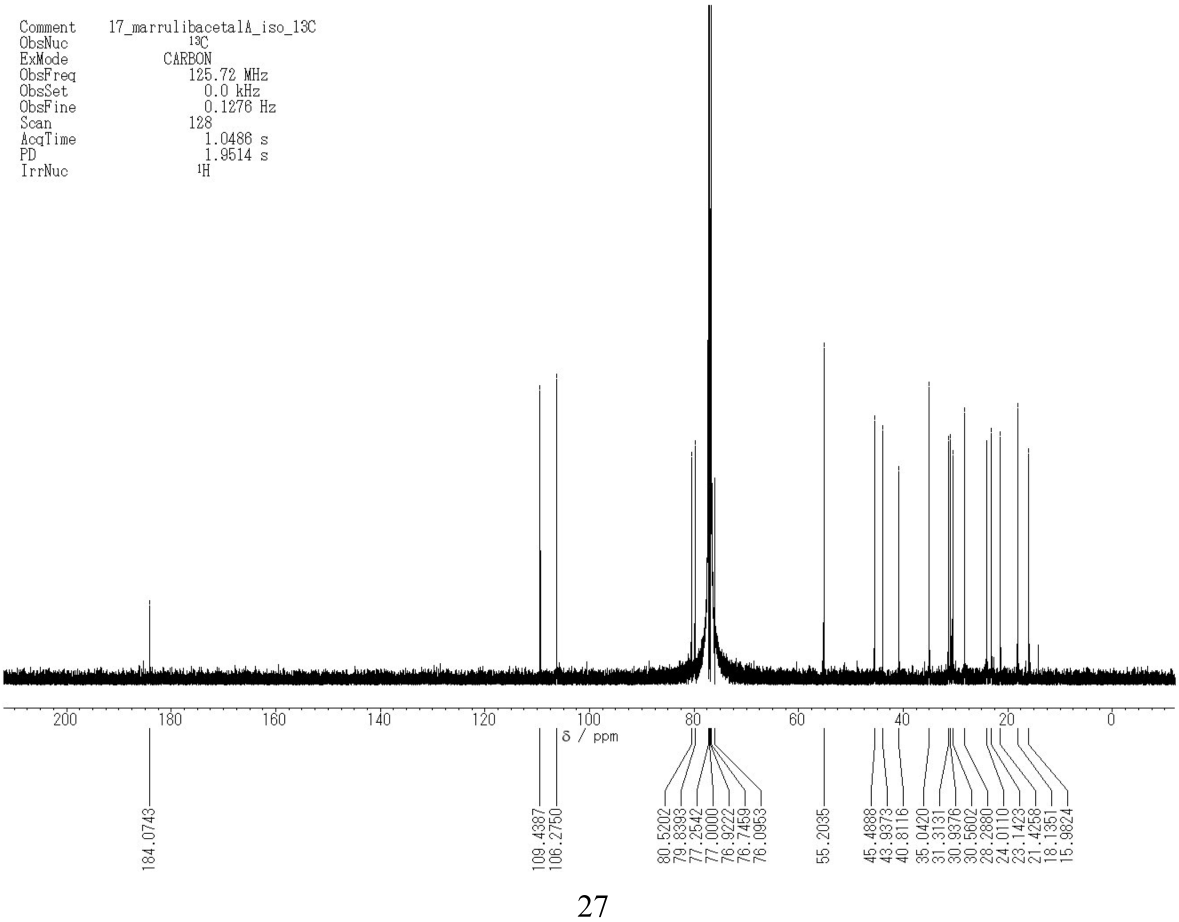

```
Comment    18_marrubashF_1H
ObsNuc     1H
ExMode     PROTON
ObsFreq    499.92 MHz
ObsSet     0.0 kHz
ObsFine    13.8008 Hz
Scan       16
AcqTime    4.0894 s
PD         1.5 s
IrrNuc     13C
```

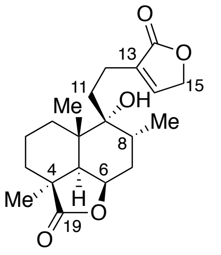

marrubasch F (**19**)

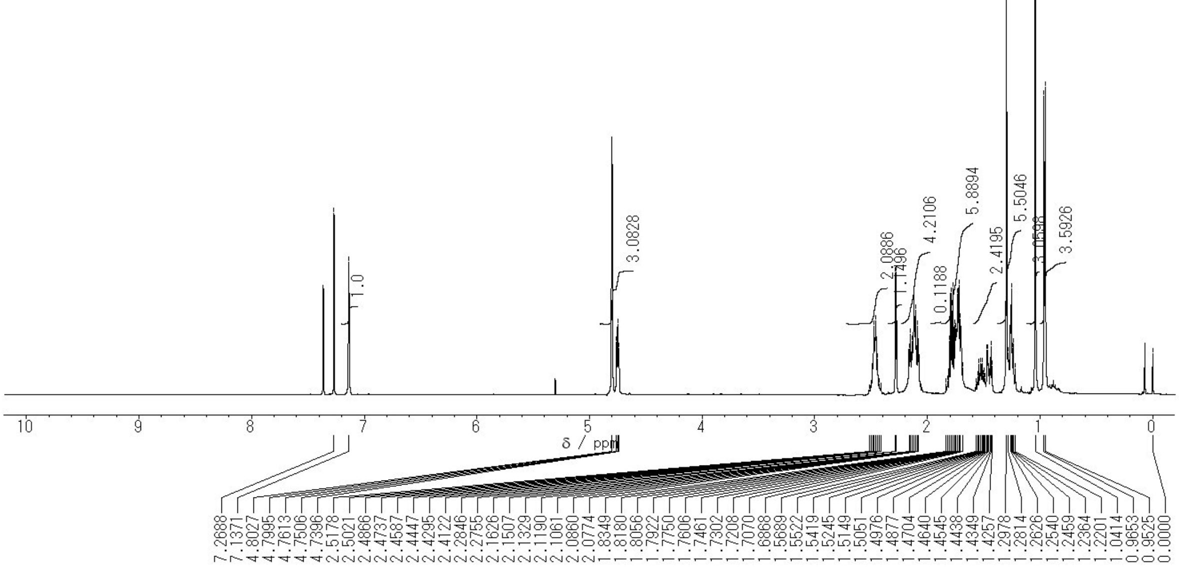


```
Comment    18_marrubashF_13C
ObsNuc     13C
ExMode     CARBON
ObsFreq    125.72 MHz
ObsSet     0.0 kHz
ObsFine    0.1276 Hz
Scan       256
AcqTime    1.0486 s
PD         1.0 s
IrrNuc     1H
```

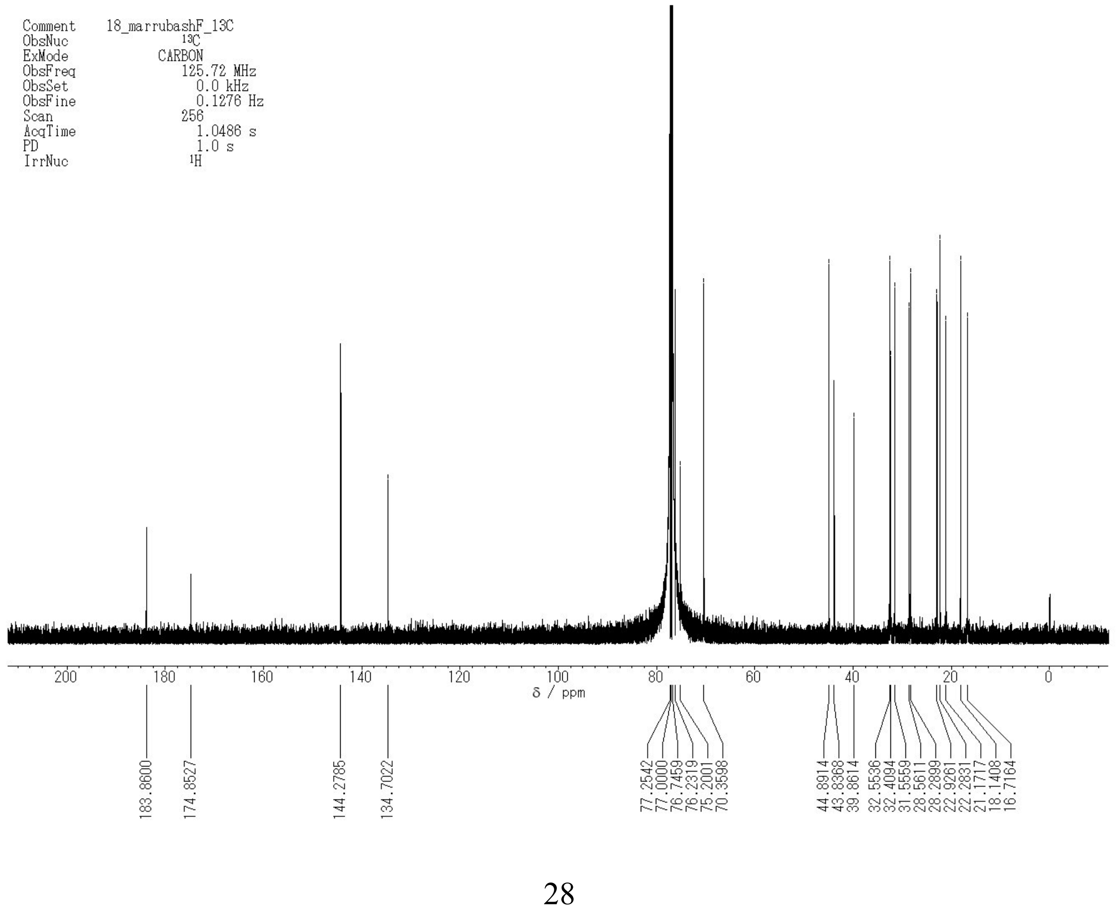

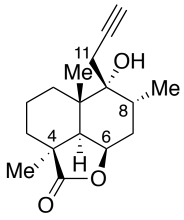

43

Comment 19_alkyne_1H
ObsNuc ^{1}H
ExMode PROTON
ObsFreq 499.68 MHz
ObsSet 0.0 kHz
ObsFine 10.3078 Hz
Scan 16
AcqTime 4.0894 s
PD 1.5 s
IrrNuc ^{13}C

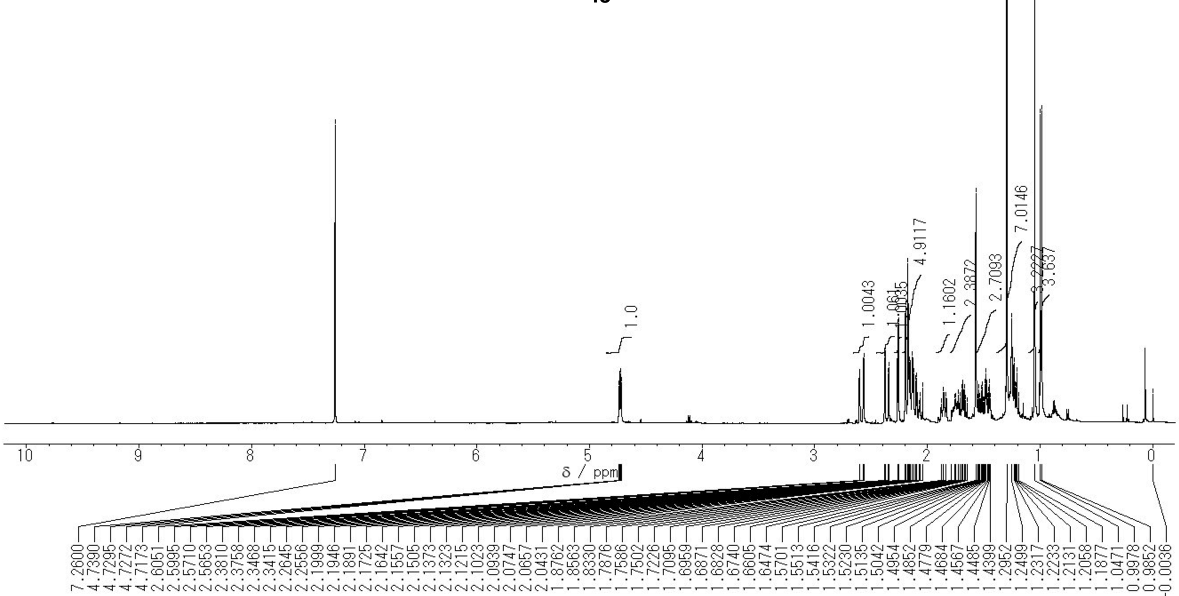

Comment 19_alkyne_13C
ObsNuc ^{13}C
ExMode CARBON
ObsFreq 125.66 MHz
ObsSet -1.0 kHz
ObsFine 997.2565 Hz
Scan 256
AcqTime 1.0486 s
PD 1.9514 s
IrrNuc ^{1}H

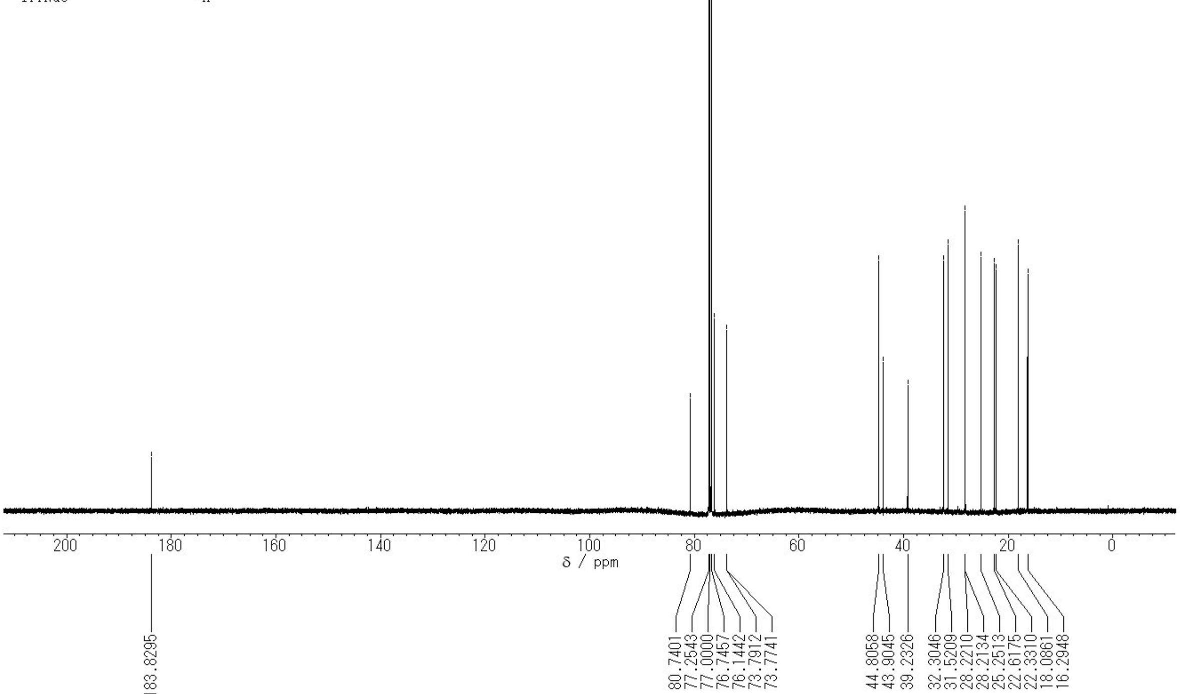

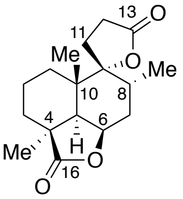

cyllenine C (**12**)

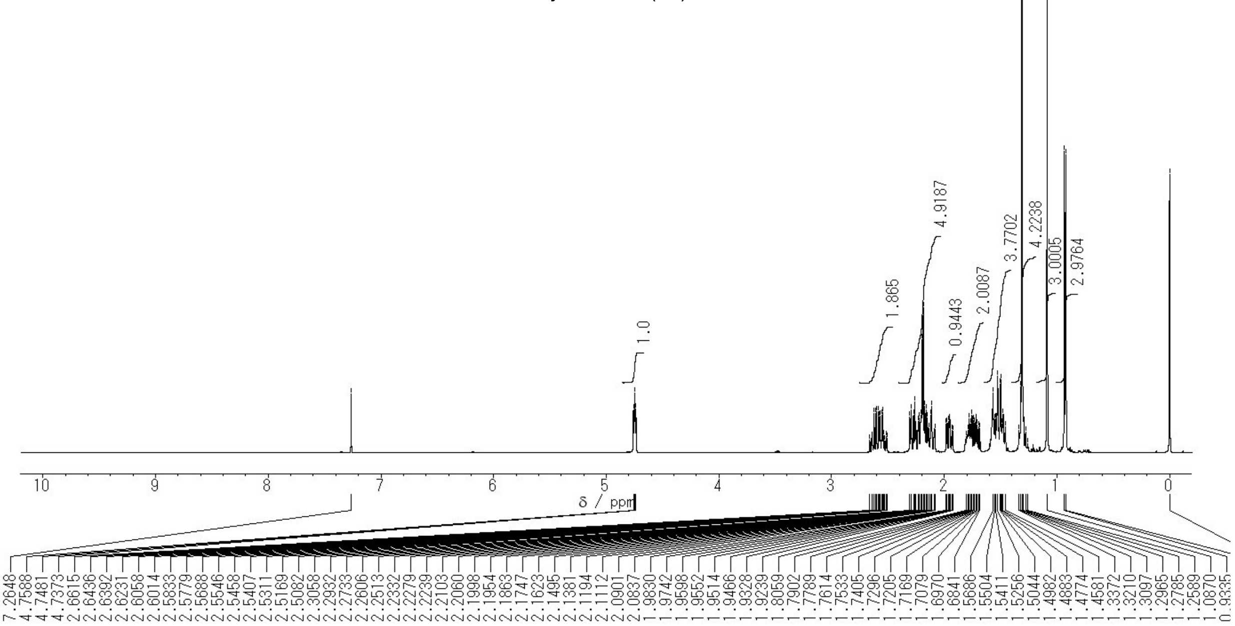

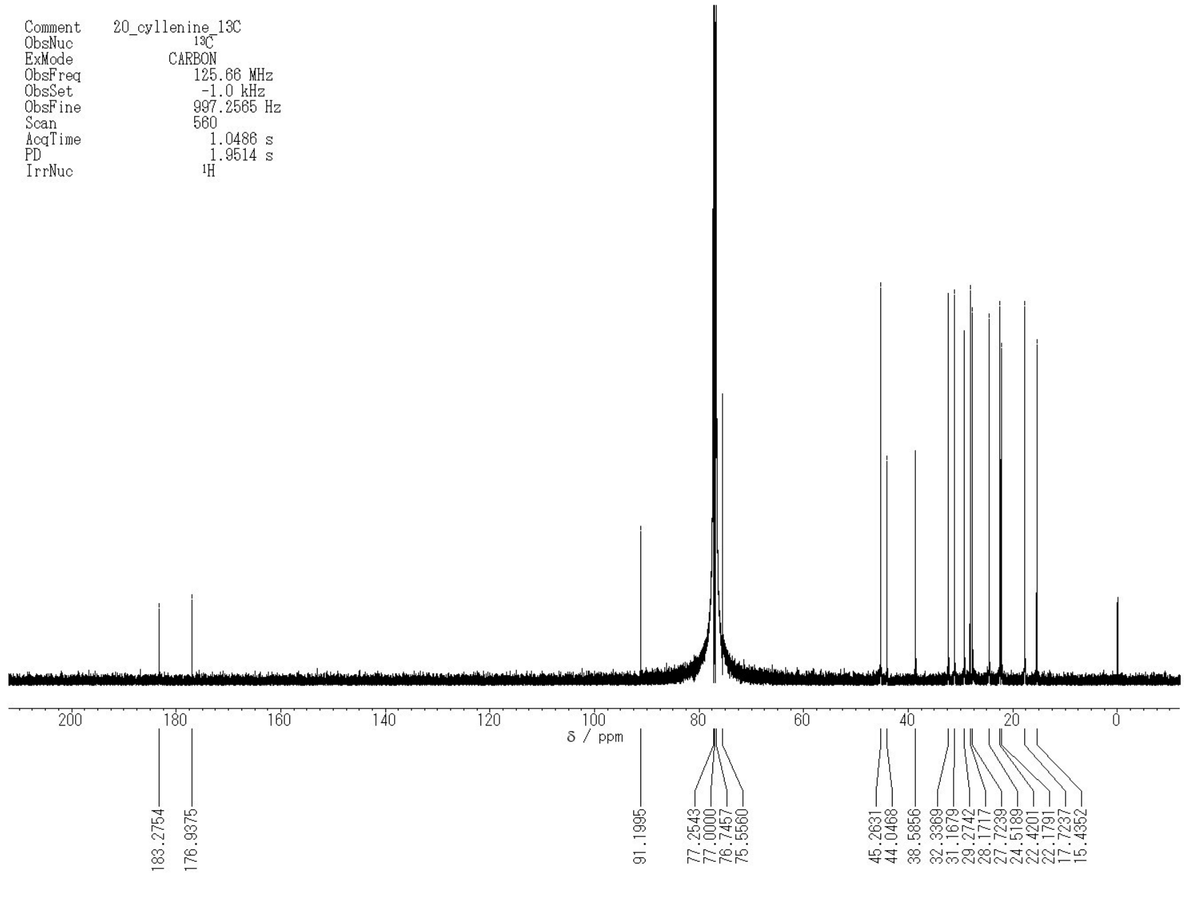

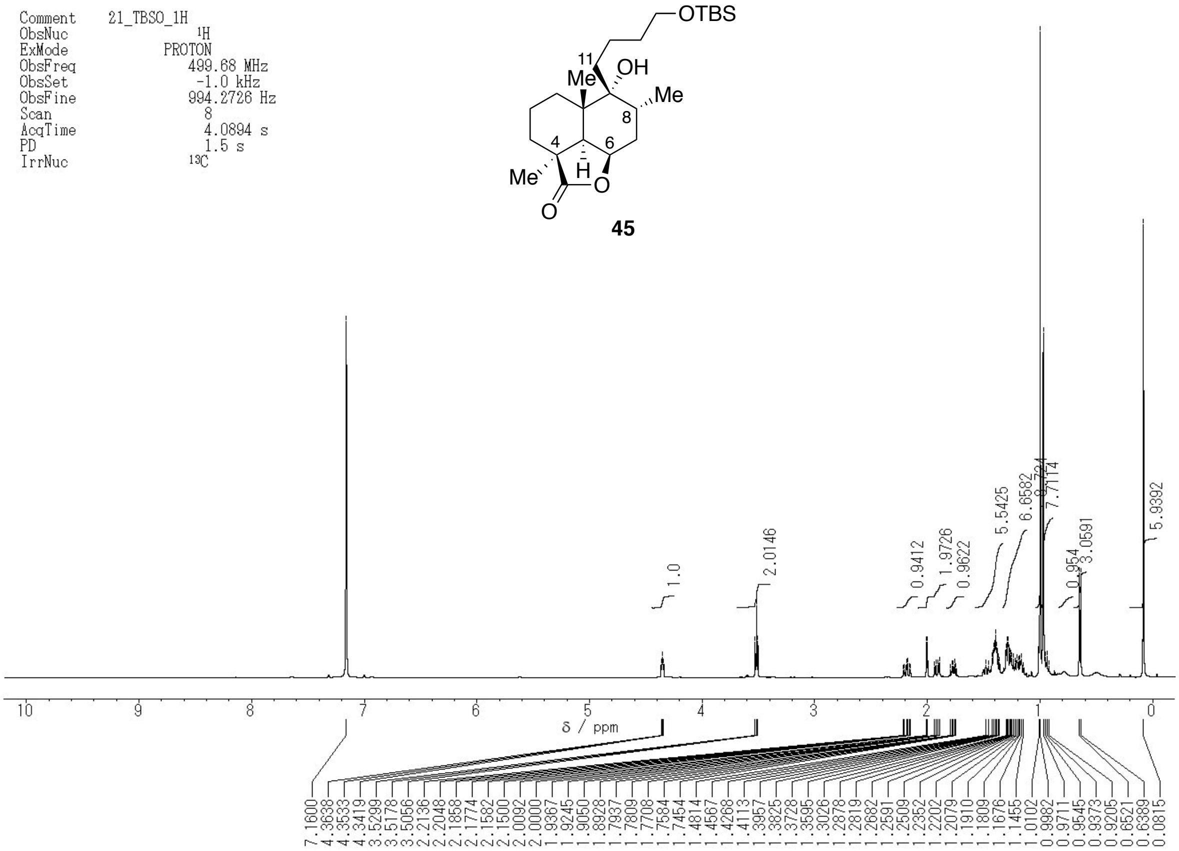

Comment 21_TBSO_1H
ObsNuc 1H
ExMode PROTON
ObsFreq 499.68 MHz
ObsSet -1.0 kHz
ObsFine 994.2726 Hz
Scan 8
AcqTime 4.0894 s
PD 1.5 s
IrrNuc 13C
OTBS
11
OH
Me
Me
8
4
6
Me
H
O
O
45
1.0
2.0146
0.9412
1.9726
0.9622
5.5425
6.6582
7.7114
0.954
3.0591
5.9392
δ / ppm
7.1600
4.3638
4.3533
4.3419
3.5299
3.5178
3.5056
2.2136
2.2048
2.1858
2.1774
2.1582
2.1500
2.0092
2.0000
1.9367
1.9245
1.9050
1.8928
1.7937
1.7809
1.7708
1.7584
1.7454
1.4814
1.4567
1.4268
1.4113
1.3957
1.3825
1.3728
1.3595
1.3026
1.2878
1.2819
1.2682
1.2591
1.2509
1.2352
1.2202
1.2079
1.1910
1.1809
1.1676
1.1455
1.0102
0.9982
0.9711
0.9545
0.9373
0.9205
0.6521
0.6389
0.0815

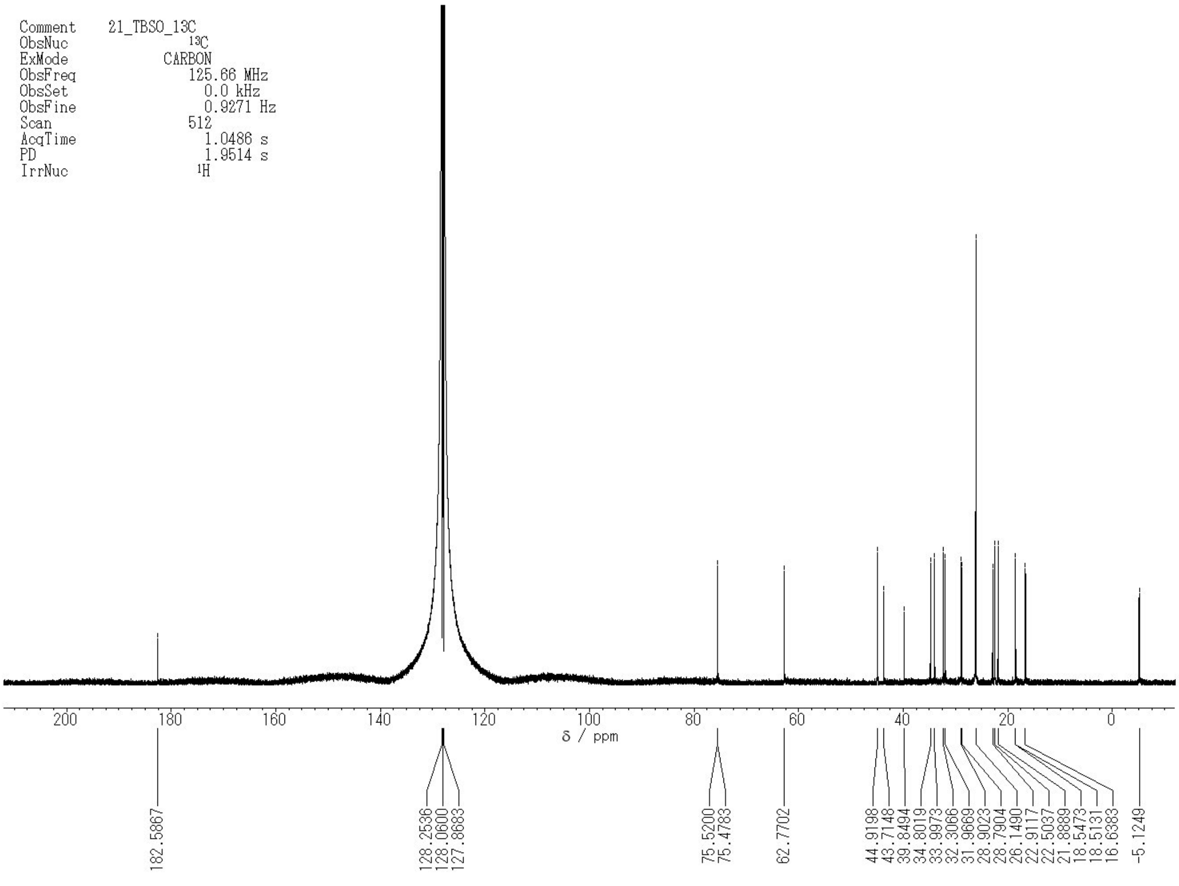

Comment 21_TBSO_13C
ObsNuc 13C
ExMode CARBON
ObsFreq 125.66 MHz
ObsSet 0.0 kHz
ObsFine 0.9271 Hz
Scan 512
AcqTime 1.0486 s
PD 1.9514 s
IrrNuc 1H
δ / ppm
182.5867
128.2536
128.0600
127.8683
75.5200
75.4783
62.7702
44.9198
43.7148
39.8494
34.8019
33.9973
32.3066
31.9669
28.9023
28.7904
26.1490
22.9117
22.5037
21.8889
18.5473
18.5131
16.6383
-5.1249

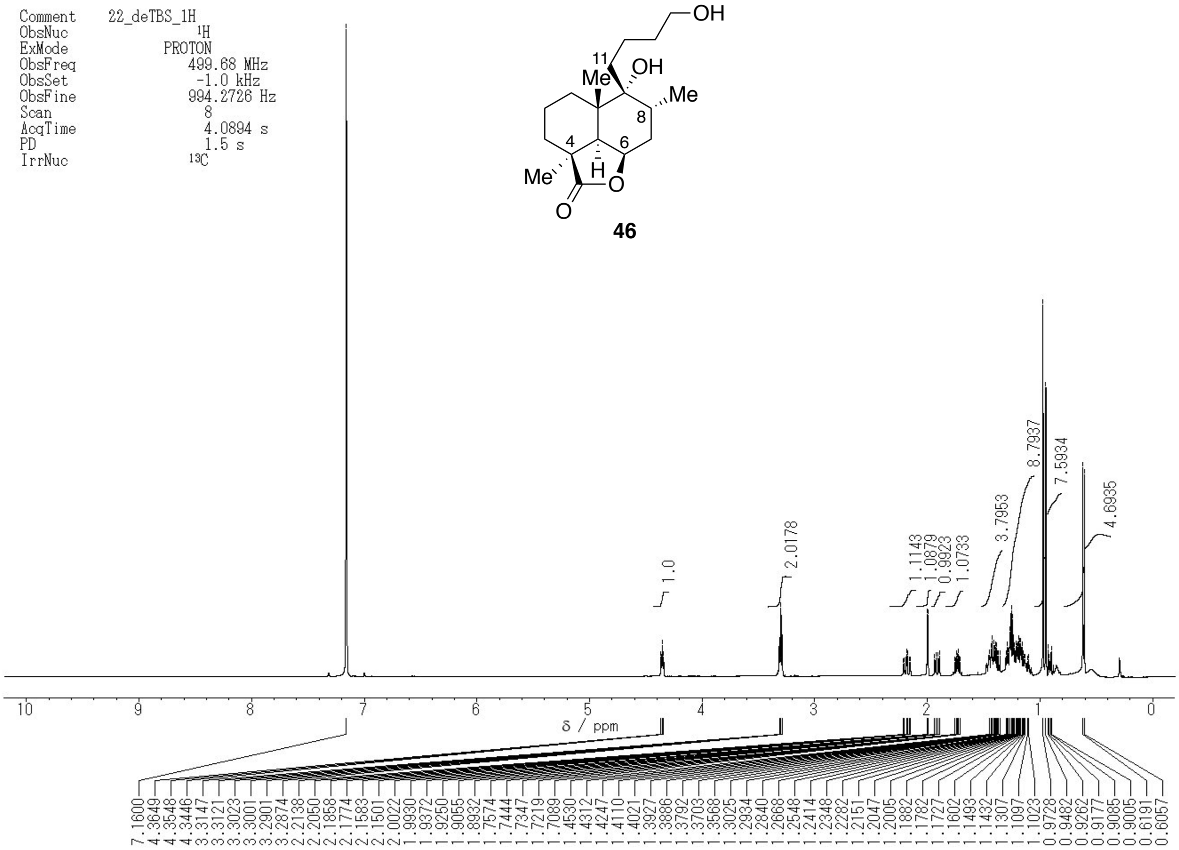
Comment 22_deTBS_1H
ObsNuc 1H
ExMode PROTON
ObsFreq 499.68 MHz
ObsSet -1.0 kHz
ObsFine 994.2726 Hz
Scan 8
AcqTime 4.0894 s
PD 1.5 s
IrrNuc 13C
OH
11
Me
OH
Me
8
4
6
Me
H
O
O
46
1.0
2.0178
1.1143
1.0879
0.9923
1.0733
3.7953
8.7937
7.5934
4.6935
δ / ppm
7.1600
4.3649
4.3548
4.3446
3.3147
3.3121
3.3023
3.3001
3.2901
3.2874
2.2138
2.2050
2.1858
2.1774
2.1583
2.1501
2.0022
1.9930
1.9372
1.9250
1.9055
1.8932
1.7574
1.7444
1.7347
1.7219
1.7089
1.4530
1.4312
1.4247
1.4110
1.4021
1.3927
1.3886
1.3792
1.3703
1.3568
1.3025
1.2934
1.2840
1.2668
1.2548
1.2414
1.2348
1.2282
1.2151
1.2047
1.2005
1.1882
1.1782
1.1727
1.1602
1.1493
1.1432
1.1307
1.1097
1.1023
0.9728
0.9482
0.9262
0.9177
0.9085
0.9005
0.6191
0.6057

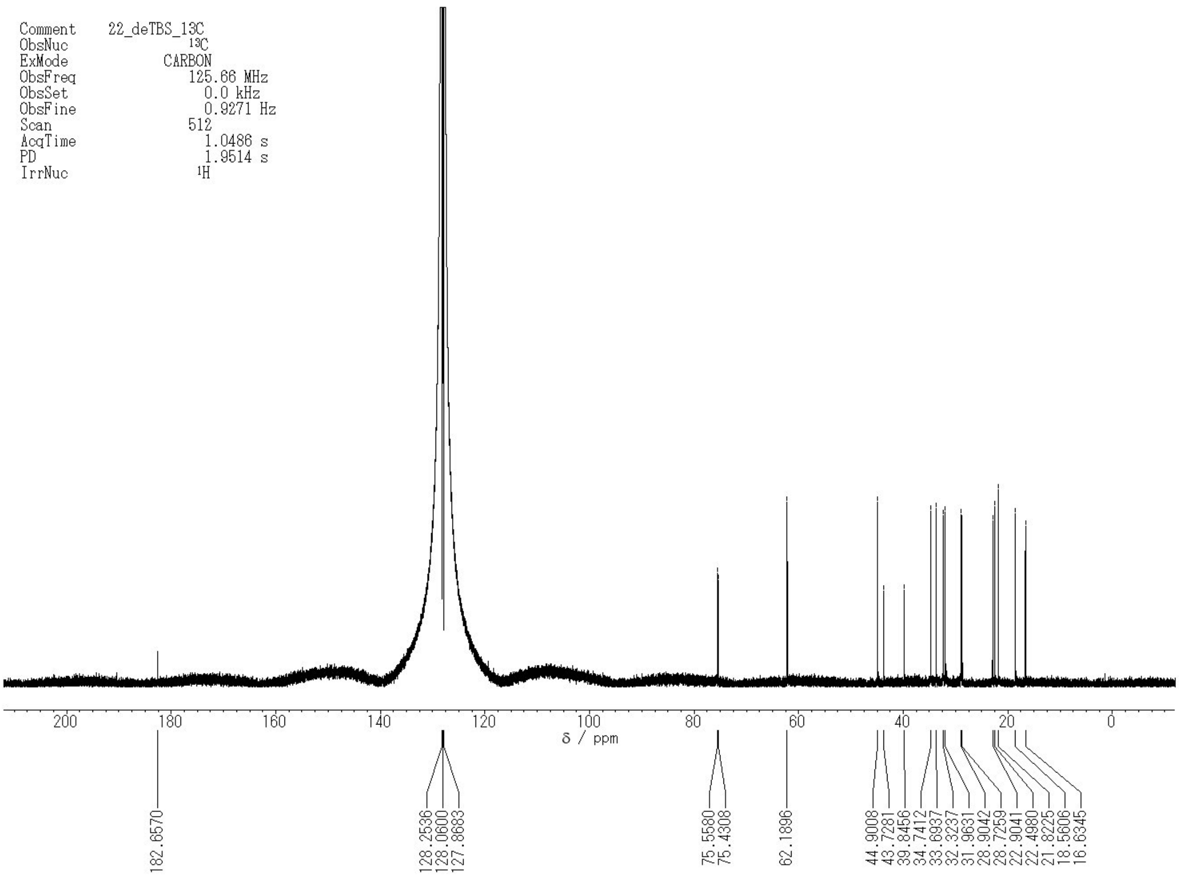
Comment 22_deTBS_13C
ObsNuc 13C
ExMode CARBON
ObsFreq 125.66 MHz
ObsSet 0.0 kHz
ObsFine 0.9271 Hz
Scan 512
AcqTime 1.0486 s
PD 1.9514 s
IrrNuc 1H
δ / ppm
182.6570
128.2536
128.0600
127.8683
75.5580
75.4308
62.1896
44.9008
43.7281
39.8456
34.7412
33.6937
32.3237
31.9631
28.9042
28.7259
22.9041
22.4980
21.8225
18.5606
16.6345

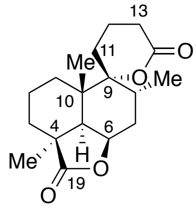

marrulactone (**14**)

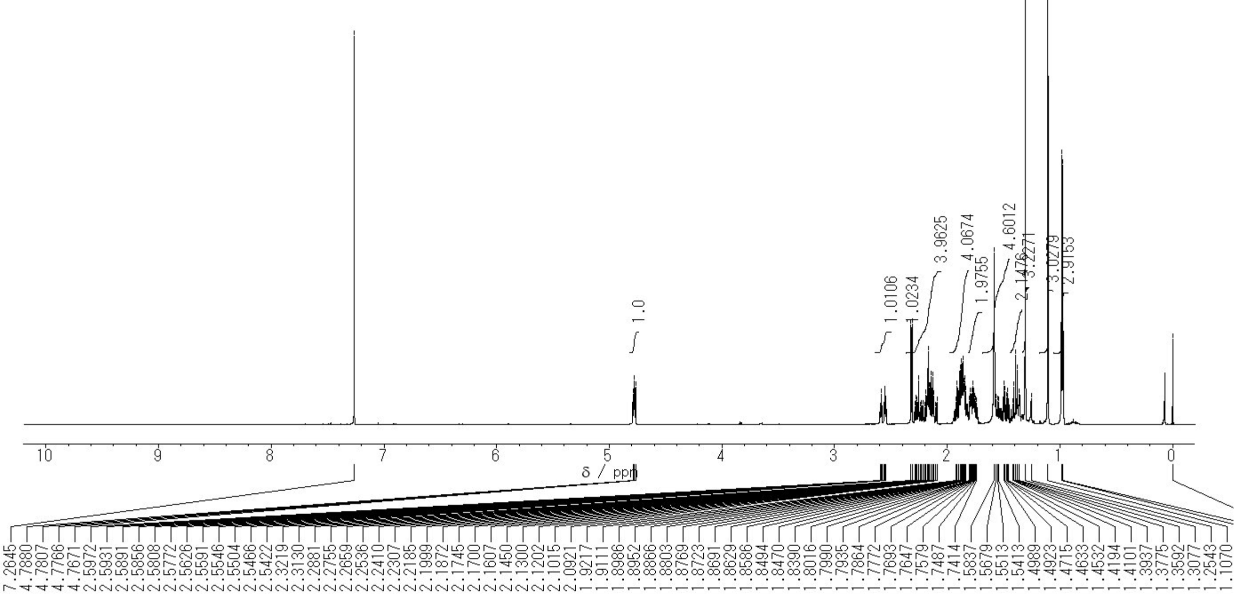

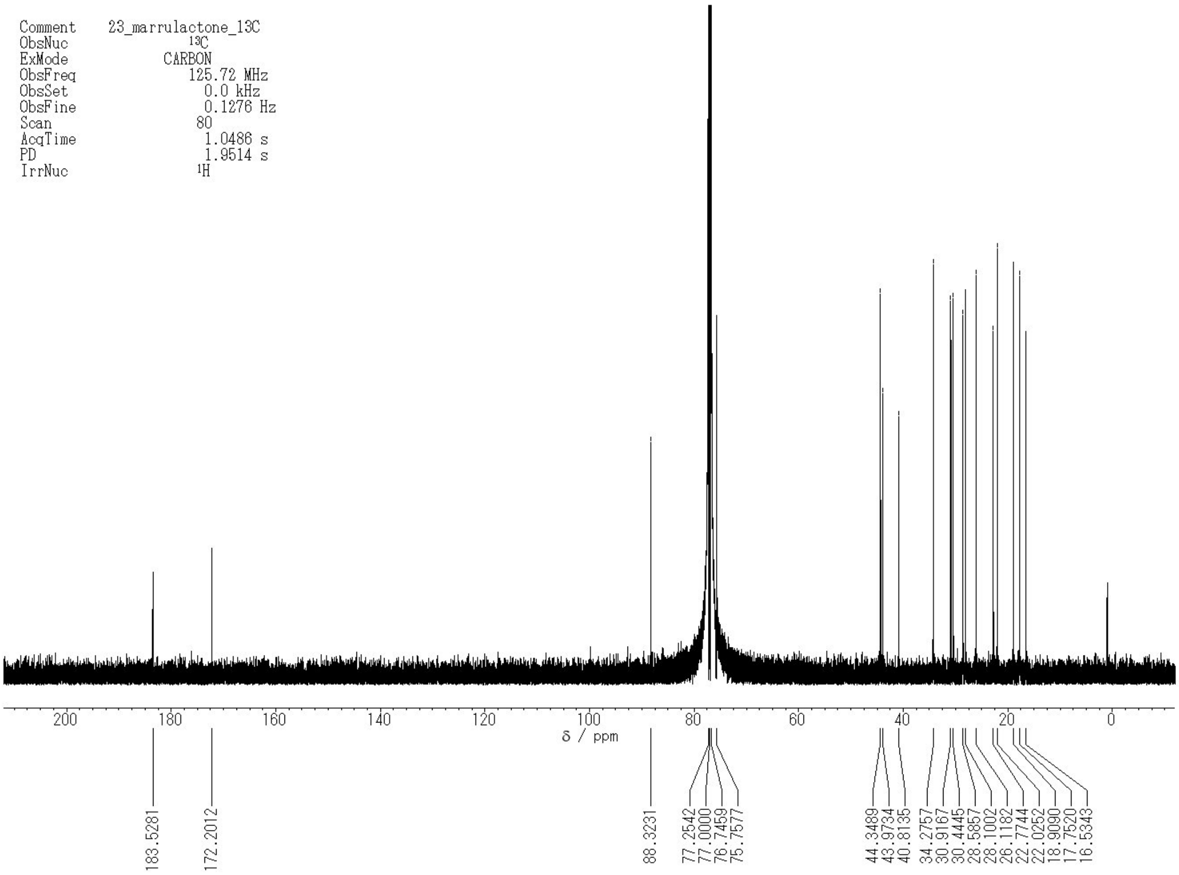

```
Comment    24_marrulanicacid_1H
ObsNuc             1H
ExMode    single_pulse.jxp
ObsFreq            500.0 MHz
ObsSet             162.0 kHz
ObsFine            416.009 Hz
Scan               8
AcqTime            1.7459 s
PD                 5.0 s
IrrNuc             NUL
```

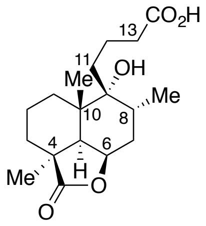

marrulanic acid (**10**)

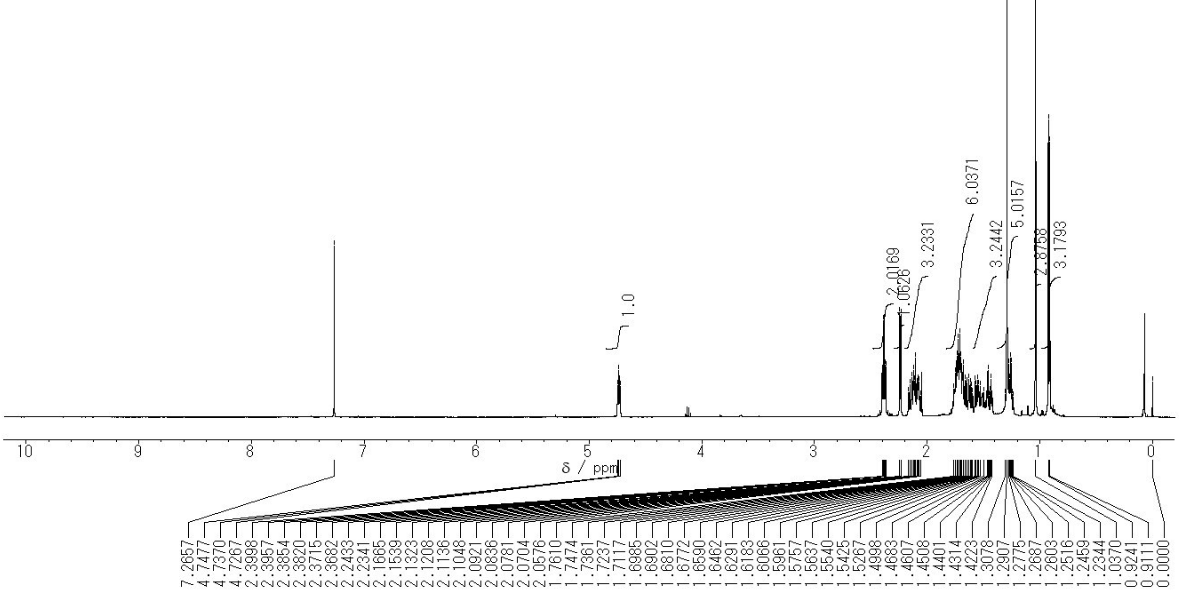


```
Comment    24_marrulanicacid_13C
ObsNuc             13C
ExMode    single_pulse_dec.jxp
ObsFreq            125.0 MHz
ObsSet             777.0 kHz
ObsFine            874.213 Hz
Scan               64
AcqTime            0.8284 s
PD                 2.0 s
IrrNuc             NUL
```

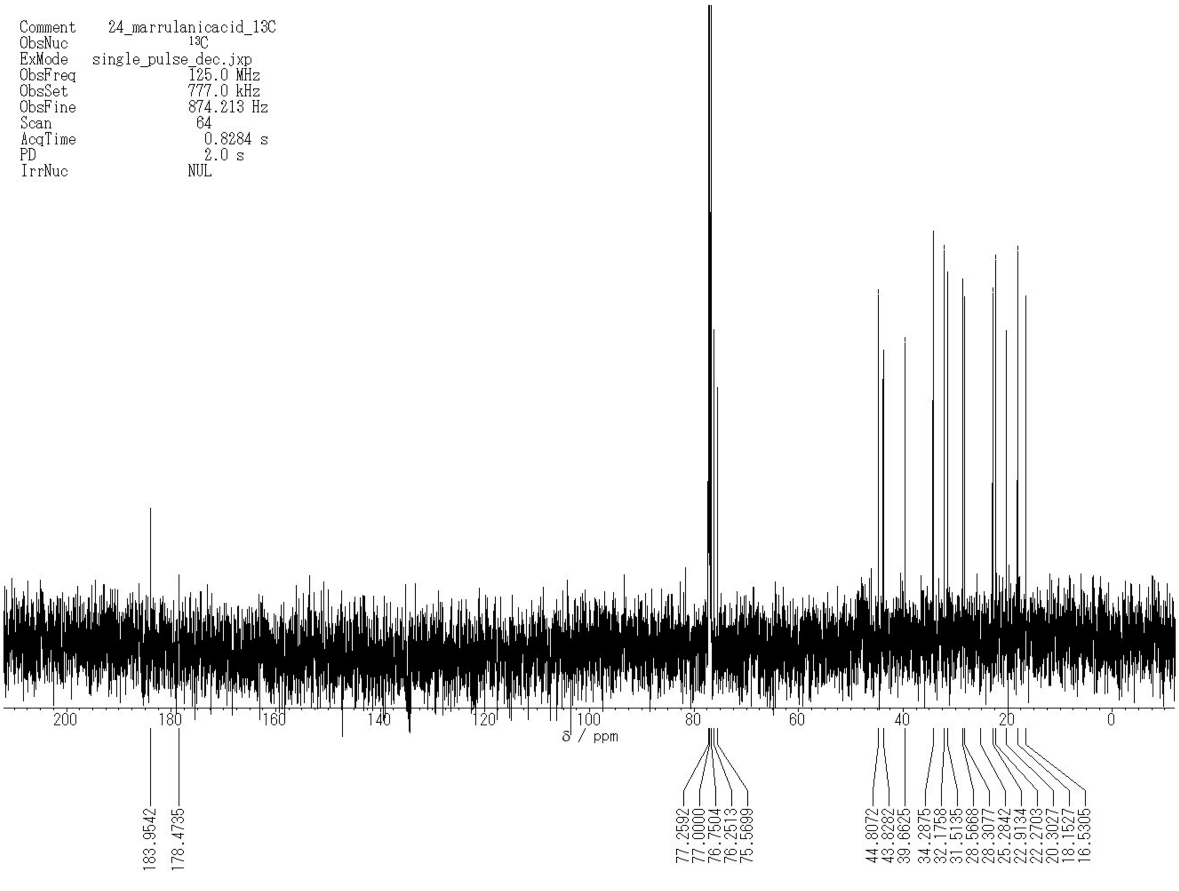

Comment 26_desertine_1H_
ObsNuc 1H
ExMode PROTON
ObsFreq 499.68 MHz
ObsSet 0.0 kHz
ObsFine 10.3078 Hz
Scan 32
AcqTime 4.0894 s
PD 1.5 s
IrrNuc 13C

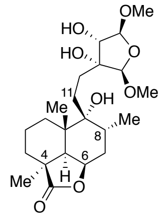

desertine (**18**)

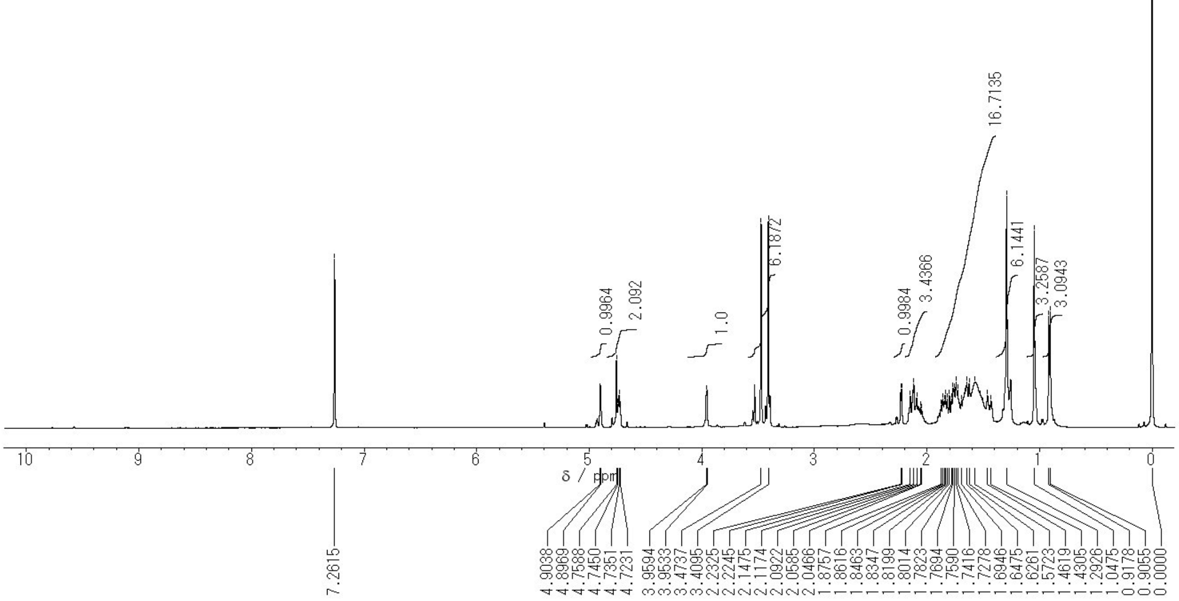

Comment 26_desertine_13C_
ObsNuc 13C
ExMode CARBON
ObsFreq 125.66 MHz
ObsSet -1.0 kHz
ObsFine 997.2565 Hz
Scan 1424
AcqTime 1.0486 s
PD 1.9514 s
IrrNuc 1H

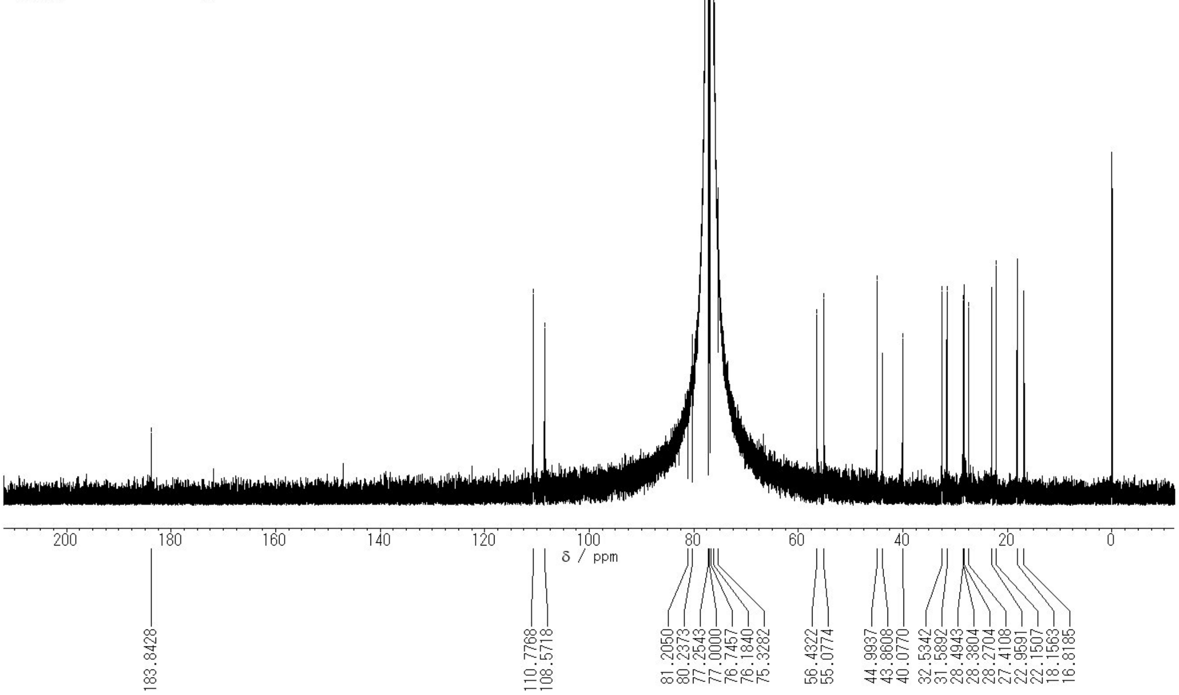

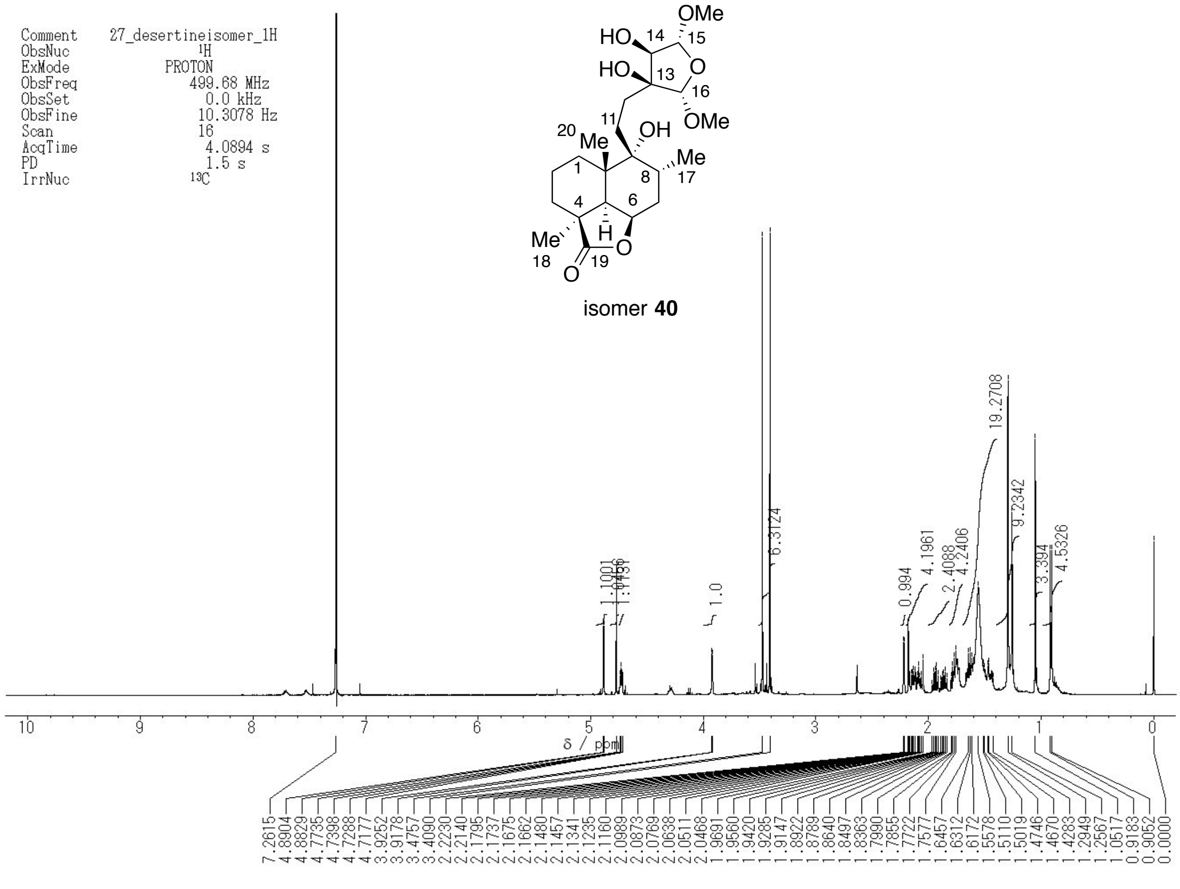
Comment 27_desertineisomer_1H
ObsNuc 1H
ExMode PROTON
ObsFreq 499.68 MHz
ObsSet 0.0 kHz
ObsFine 10.3078 Hz
Scan 16
AcqTime 4.0894 s
PD 1.5 s
IrrNuc 13C
OMe
HO 14 15
HO 13 O
16
11
OMe
20 Me OH
1 Me
8 17
4 6
H
Me
18 19 O
O
isomer 40
1.1001
1.0
6.3124
0.994
4.1961
2.4088
4.2406
19.2708
9.2342
3.394
4.5326
δ / ppm
7.2615
4.8904
4.8829
4.7735
4.7398
4.7288
4.7177
3.9252
3.9178
3.4757
3.4090
2.2230
2.2140
2.1795
2.1737
2.1675
2.1662
2.1480
2.1457
2.1341
2.1235
2.1160
2.0989
2.0873
2.0769
2.0638
2.0511
2.0468
1.9691
1.9560
1.9420
1.9285
1.9147
1.8922
1.8789
1.8640
1.8497
1.8363
1.7990
1.7855
1.7722
1.7577
1.6457
1.6312
1.6172
1.5578
1.5110
1.5019
1.4746
1.4670
1.4283
1.2949
1.2567
1.0517
0.9183
0.9052
0.0000

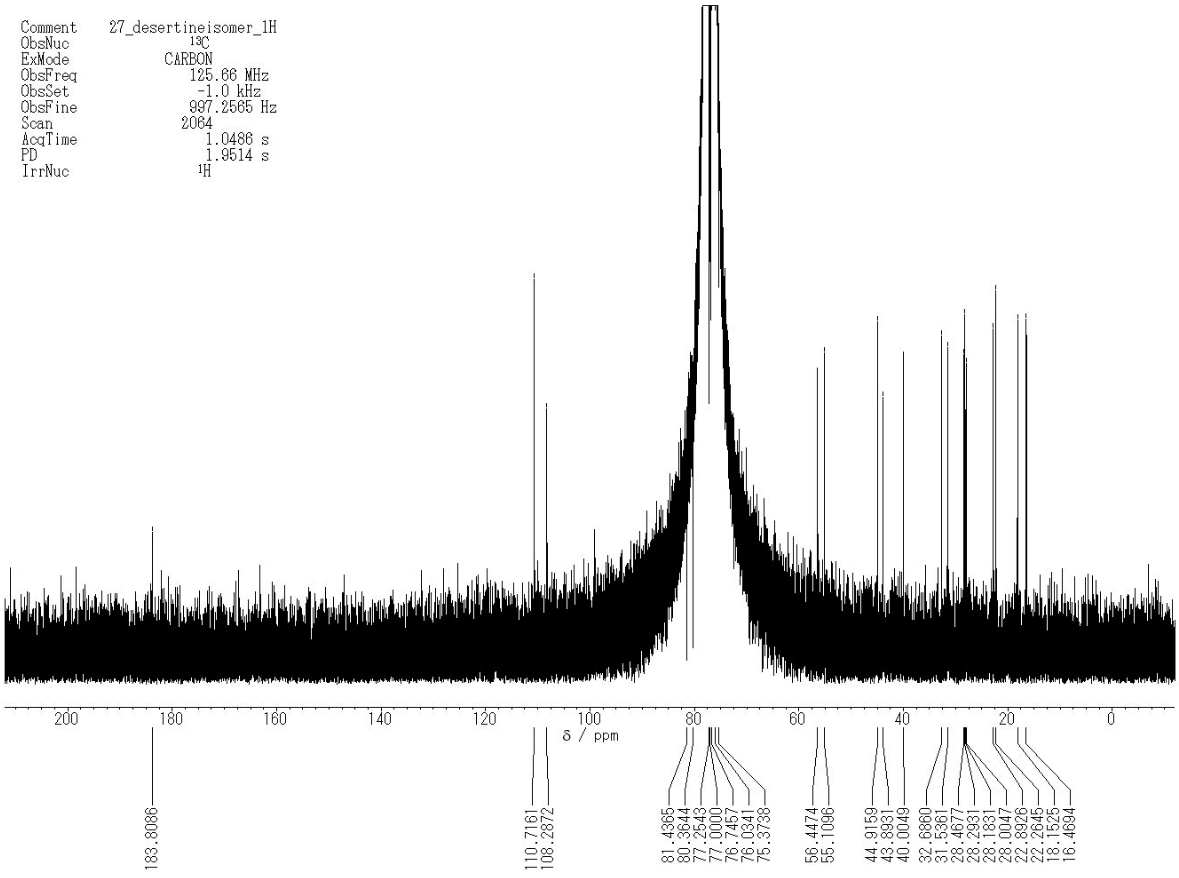
Comment 27_desertineisomer_1H
ObsNuc 13C
ExMode CARBON
ObsFreq 125.66 MHz
ObsSet -1.0 kHz
ObsFine 997.2565 Hz
Scan 2064
AcqTime 1.0486 s
PD 1.9514 s
IrrNuc 1H
δ / ppm
183.8086
110.7161
108.2872
81.4365
80.3644
77.2543
77.0000
76.7457
76.0341
75.3738
56.4474
55.1096
44.9159
43.8931
40.0049
32.6860
31.5361
28.4677
28.2931
28.1831
28.0047
22.8926
22.2645
18.1525
16.4694
